# The Atypical Calpains: Evolutionary Analyses and Roles in *Caenorhabditis elegans* Cellular Degeneration

**DOI:** 10.1371/journal.pgen.1002602

**Published:** 2012-03-29

**Authors:** Peter I. Joyce, Rahul Satija, Maozi Chen, Patricia E. Kuwabara

**Affiliations:** 1 School of Biochemistry, University of Bristol, Bristol, United Kingdom; 2 Medical Research Council Mammalian Genetics Unit, Harwell, United Kingdom; 3 Broad Institute, Massachusetts Institute of Technology, Cambridge, Massachusetts, United States of America; University of California San Diego, United States of America

## Abstract

The calpains are physiologically important Ca^2+^-activated regulatory proteases, which are divided into typical or atypical sub-families based on constituent domains. Both sub-families are present in mammals, but our understanding of calpain function is based primarily on typical sub-family members. Here, we take advantage of the model organism *Caenorhabditis elegans*, which expresses only atypical calpains, to extend our knowledge of the phylogenetic evolution and function of calpains. We provide evidence that a typical human calpain protein with a penta EF hand, detected using custom profile hidden Markov models, is conserved in ancient metazoans and a divergent clade. These analyses also provide evidence for the lineage-specific loss of typical calpain genes in *C. elegans* and Ciona, and they reveal that many calpain-like genes lack an intact catalytic triad. Given the association between the dysregulation of typical calpains and human degenerative pathologies, we explored the phenotypes, expression profiles, and consequences of inappropriate reduction or activation of *C. elegans* atypical calpains. These studies show that the atypical calpain gene, *clp-1*, contributes to muscle degeneration and reveal that *clp-1* activity is sensitive to genetic manipulation of [Ca^2+^]_i_. We show that CLP-1 localizes to sarcomeric sub-structures, but is excluded from dense bodies (Z-disks). We find that the muscle degeneration observed in a *C. elegans* model of dystrophin-based muscular dystrophy can be suppressed by *clp-1* inactivation and that nemadipine-A inhibition of the EGL-19 calcium channel reveals that Ca^2+^ dysfunction underlies the *C. elegans* MyoD model of myopathy. Taken together, our analyses highlight the roles of calcium dysregulation and CLP-1 in muscle myopathies and suggest that the atypical calpains could retain conserved roles in myofilament turnover.

## Author Summary

Calpains are calcium activated non-lysosomal proteases that cleave proteins with exquisite selectivity. Proteins can be activated by calpain cleavage, because they are released from inhibitory constraints, or they can be targeted for further degradation to facilitate their normal physiological turnover or to promote cellular remodelling. Inappropriate calpain activity can lead to degenerative pathologies and cancers. Our understanding of calpain function is based primarily on typical calpains, which carry EF hand motifs that bind Ca^2+^ or mediate dimerization; however, typical and atypical calpains, which lack EF hand motifs, are both present in mammals. Hence, any therapeutic intervention designed to suppress degenerative conditions, particularly those caused by elevated Ca^2+^ levels, should also consider the potential involvement of atypical calpains. We have taken advantage of the model organism *C. elegans*, which only encodes atypical calpain proteins, to gain an understanding of the evolution and activities of these proteins. We show that the CLP-1 atypical calpain is normally expressed in muscle and localizes to sarcomeric sub-structures. We find that CLP-1 contributes to the muscle degeneration observed in a model of Duchenne muscular dystrophy. Our studies also highlight the importance of calcium dysregulation in promoting CLP-1 activity and muscle degeneration.

## Introduction

Calpains are Ca^2+^-regulated neutral thiol proteases that perform a limited digestion of target substrates, and are thus considered to be regulatory, as opposed to strictly degradative [1]. Members of the calpain family are variously composed of discrete modular domains, numbered DI–DVI (Figure S1) [2], [3]; DI–DIV are domains associated with the large calpain catalytic subunit and DV–DVI are domains found in a common small regulatory subunit, named CAPNS1 (not shown) [4], [5]. All calpain large subunits share a signature domain DII, which contains the core catalytic triad of cysteine, histidine and asparagine [6], [7]. DII is further divided into subdomains, IIa and IIb, which change conformation to align the catalytic cleft upon binding of Ca^2+^ [8]. DIII has a C2-like domain, which is present in almost all calpains, whereas DI is usually composed of a short non-conserved sequence that can undergo autolysis [6], [7]. As discussed below, calpains differ in their domain architecture; however, a key feature that distinguishes the typical calpains from the atypical calpains is the presence of DIV. The most extensively studied calpains are the typical CAPN1 and CAPN2, which have a classic DIV composed of five EF hand motifs, also referred to as a penta EF hand (PEF) [4], [5]. A PEF domain is also present in DVI of CAPNS1, which heterodimerizes with CAPN1 and CAPN2 through DIV [4], [5], although not all typical calpains require CAPNS1 for activity [9]–[11]. Mammalian genomes also encode the endogenous inhibitor calpastatin, which is specific for typical calpains [12], [13].

By contrast to typical calpains, there is no evidence indicating that atypical calpains lacking DIV and EF hand motifs form heterodimers. This absence of EF hands was also responsible for the initial belief that atypical calpains were insensitive to Ca^2+^ regulation [1]. Both typical and atypical calpains can also carry a variety of alternative domains, including an additional C2-like domain, zinc fingers, glycine rich regions and microtubule interacting and transport (MIT) domains (Figure S1).

Genes encoding predicted calpain proteins have been identified in many organisms ranging from single-celled yeasts to higher vertebrates through the sequencing of whole genomes. Mammalian genomes encode nine typical and five atypical calpains [1], [14], [15]. The Drosophila genome encodes only three typical and one atypical calpain (CALPD), also known as small optic lobes (SOL) [16]. By contrast, the genome of the nematode *C. elegans* encodes multiple atypical calpains, but no typical calpains. Given the paucity of typical calpains in non-mammals, phylogenetic arguments have been presented to suggest that the EF hand motifs of typical calpains were late evolutionary additions [14]. Homologs of CAPNS1 and calpastatin, proteins that interact with typical calpain subunits, are also absent in *C. elegans* and *Drosophila* [16], [17].

Mammalian calpains participate in many cellular processes, including, but not limited to, cytoskeletal remodelling [18], cell mobility [19], myofibril maintenance [20], signal transduction [21], cell cycle progression [22], regulation of gene expression [15], apoptosis [23] and long term potentiation [24]. Genetic association studies have implicated calpains in disease pathologies including limb-girdle muscular dystrophy type 2A (LGMD2A, *CAPN3*) [25], susceptibility to type II diabetes (*CAPN10*) [26] and gastric cancer (*CAPN8/9*) [27], [28].

Despite their involvement in cell maintenance and disease, it has been difficult to predict the targets of calpain proteolysis with any precision, because substrate specificity is only weakly determined by primary sequence [29], [30]. In addition, a large number of in vitro substrates have been identified, but many await in vivo validation. Chemical calpain inhibitors have also been developed, including active site peptidomimetics and domain IV and VI non-peptidyl inhibitors [31]. Unfortunately, active site inhibitors are generally not specific for calpains, as other cysteine proteases, such as members of the cysteine cathepsin family, are also subject to inhibition [32]; in addition, DIV and DVI inhibitors would fail to inhibit atypical calpains.

By contrast to the many studies focused on the activity of the typical calpains, it is clear that the specific involvement of atypical calpains in development and disease pathologies remains underexplored. We are using *C. elegans* as a model for exploring the function of atypical calpains. Previous work in *C. elegans* has highlighted the importance of the *tra-3/clp-5* calpain gene in sex determination [33], [34]; other studies have shown that the *C. elegans clp-1* and *tra-3/clp-5* genes are involved in neurodegeneration and necrosis of a subset of vulval cells and in the intestine [35]–[37]. In this paper, we have examined the evolution of atypical and typical calpains in metazoa and analyzed the effects of reducing or enhancing the activities of atypical calpains on *C. elegans* development. To gain information about the potential site of action of a subset of atypical calpain genes, we have examined their in vivo expression patterns. Given the association between calpain activity and degenerative pathologies, we have investigated factors that influence the activity of *clp-1* in a *C. elegans* model of muscular dystrophy and reveal the importance of both sustained calpain expression and intracellular Ca^2+^ levels ([Ca^2+^]_i_). Taken together, we hypothesize that the muscle degenerative phenotype caused by ectopic *clp-1* stems from a conserved physiological role for calpain in the turnover of sarcomeric muscle proteins.

## Results

### The *C. elegans* atypical calpain family

We searched the *C. elegans* genome sequence using the typical human CAPN1 sequence and identified 14 atypical calpain-like sequences. Seven of these genes had previously been named *clp-1* to *clp-7*, so we named the remaining seven genes *clp-8* to *clp-10* and *clpr-1* to *clpr-4* (for *clp-related*) for reasons explained below (Figure S1). An earlier analysis predicted the existence of 17 *C. elegans* calpain-like sequences [35]; however, three of these genes are not valid family members. F44F1.1 is now considered to be a pseudogene (Wormbase, release WS225), and both M04F3.4 and T21H3.3 lack a catalytic domain II, although they carry EF hand motifs. The domain architecture of the *C. elegans* atypical calpain proteins and the typical and atypical calpain proteins found in humans and *Drosophila* is shown in Figure S1 for comparison; a multisequence alignment is also provided to highlight the conservation of catalytic domain II and the divergence of DI and DIII among members of the calpain superfamily (Figure S2).

The genome of *C. briggsae*, a nematode closely related to *C. elegans* [38], only encodes nine predicted calpain sequences. To explain this difference, we compared the sequences of calpain proteins from *C. elegans*, *C. briggsae*, *Drosophila* and also human, and generated a cladogram (Figure S3). By comparison to *C. briggsae*, it appears that a recent gene expansion specific to *C. elegans* created two paralogous gene clusters. The first cluster consists of the *C. elegans* CLP-9 (*T11A5.6*) and CLP-10 (*W05G11.4*) proteins, which are homologous to *C. briggsae* Cbr-G19393; the proteins in this cluster are each predicted to have a SolH domain. The second cluster includes *clp-8* (*F44F1.3*) and four predicted *clpr* genes, which are missing a critical cysteine residue of the catalytic triad; together these genes are paralogous to Cbr-G04776 and Cbr-G00485, which encode proteins with an intact catalytic triad. The existence of *clpr* genes raises the possibility that these predicted calpains are inactive, or that they have possibly gained novel non-proteolytic activities (Figure S1).

The cladogram in Figure S3 highlights the phylogenetic relationships between the Caenorhabditis, Drosophila and human calpain proteins. Although typical calpains are absent in *C. elegans*, the CLP-2, TRA-3/CLP-5 and the paralogous CLP-9 and CLP-10 proteins share extensive homology (E-values<1e-94) with human CAPN7, CAPN5/CAPN6 and CAPN15, respectively (Figure S3). Little is known about the biological functions of these human atypical calpains; hence, an analysis of their *C. elegans* homologs would provide potential insights into their function.

### Ancient metazoan origins of typical and atypical calpains

**Figure 1:**
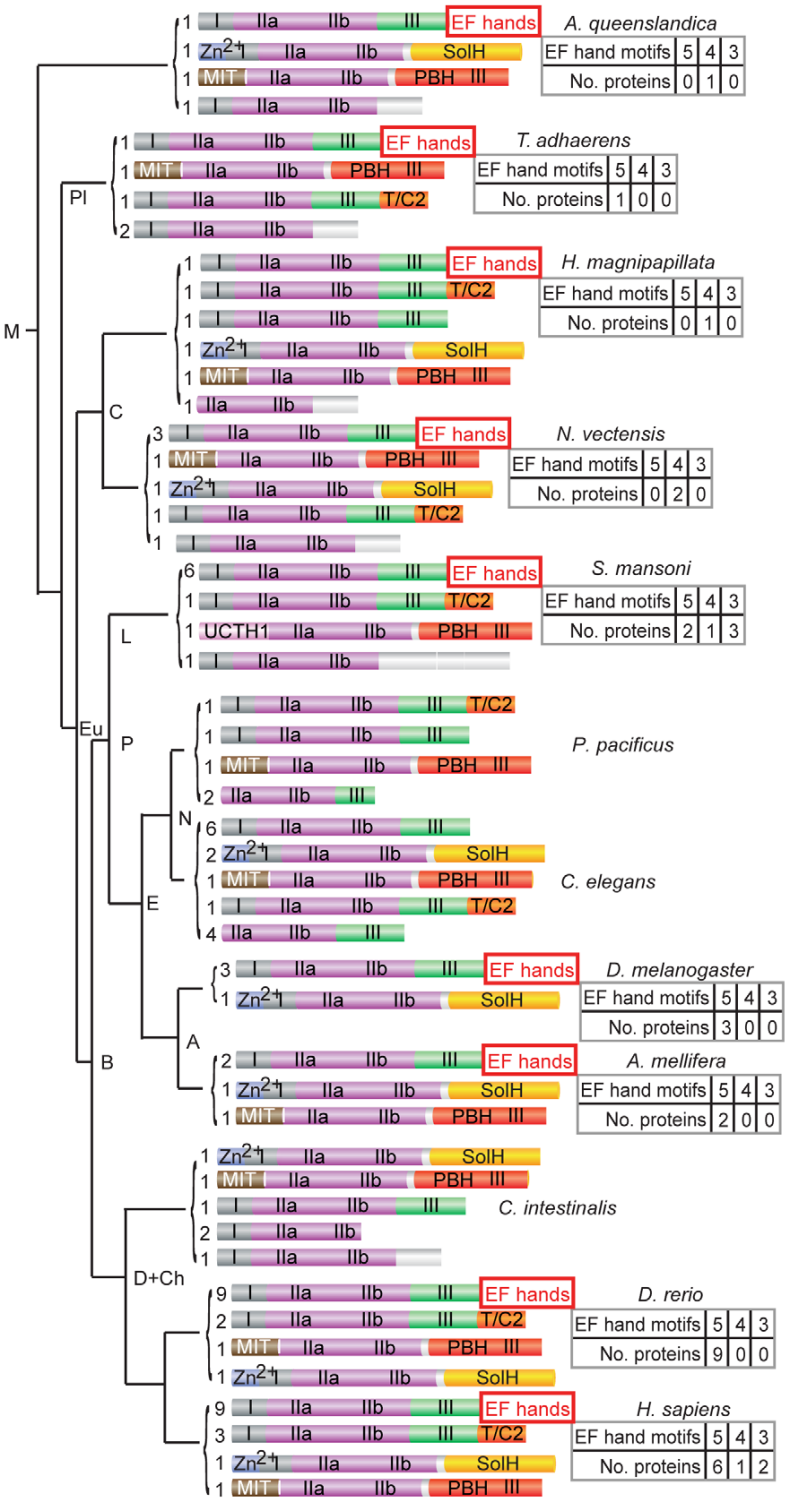
Phylogenetic analyses of typical calpains in metazoa support a model of lineage-specific loss. Typical calpain EF hand motifs in DIV are highlighted; C-terminal EF hand motifs are absent in Nematoda and *Ciona intestinalis*. Other domains associated with calpain proteins include: PBH, PalB homology domain with some domain III homology; T/C2, C2 domain originally identified in TRA-3 [33]; Zn^2+^, zinc finger motif-containing; SolH, small optic lobes (SOL) homology domain; MIT 1, microtubule interacting and transport domain; and UCTH1, ubiquitin carboxyl-terminal hydrolase domain. The number of genes encoding proteins with the indicated modular arrangement of domains is indicated, left; the number of EF hands in DIV predicted by custom profile hidden Markov models are shown, right. Clades abbreviations: M, Metazoa; Pl, Placozoa; Eu, Eumetezoa; C, Cnidaria; B, Bilateria; P, Protostomia; D+Ch, Deuterostomia and Chordata; L, Lophotrochozoa; E, Ecdysozoa; N, Nematoda and A, Arthropoda. The tree is not drawn to scale. Protein accession numbers are available in Materials and Methods.

We examined the evolutionary history of the typical calpains in more depth to seek evidence that their absence in *C. elegans* might be due to a lineage specific loss. We restricted our analyses to genes in metazoan phyla, as an earlier study had shown that calpain sequences containing C-terminal EF hand motifs are absent in plants and fungi [14]. We were further aided by the availability of whole genome sequences, particularly those representative of more ancient phyla, such as the placozoan *Trichoplax adhaerens*, the cnidarians *Nematostella vectensis* and *Hydra magnipapillata* [39]–[41], and also the sponge *Amphimedon queenslandica*, which diverged from early metazoans over 600 million years ago [42]. We found that homologs of typical calpains with EF-hand domains were likely to be present not only in early metazoa, but also in sponge (Figure 1). We also noted that typical calpain genes were absent from the genomes of other nematodes, including *P. pacificus*, *B. malayi* and *C. remanei*. Surprisingly, typical calpain genes were also absent in the tunicate *C. intestinalis*, a primitive branching clade of chordates [43], further supporting the notion of lineage-specific loss of typical calpains. Genes encoding atypical calpain proteins, including those carrying SolH or PBH domains, were also found in all metazoan phyla examined and in sponge (Figure 1).

### A profile hidden Markov model detects penta EF hand domains

When analysing the phylogeny of the typical calpains, we found that it was difficult to count EF-hand motifs in order to identify penta EF-hand (PEF) domains using existing models [44]; for example, PROSITE and pfam counted only four EF-hand motifs and thus failed to identify PEF domains in CAPN1, 2 and 3 and sorcin. The number of EF hand motifs is likely to affect the ability of these proteins to dimerize, so we developed 5 separate custom profile hidden Markov models (profile HMMs) based on the individual EF hand motifs present within a penta EF hand domain to improve the ability to count EF hand motifs [45] (Text S1, see Materials and Methods). These profiles were tested by showing that together they detected the presence of penta EF hand domains in human CAPN1, CAPN2, CAPNS1 and sorcin proteins (true positives), and their absence in human CAPN10 or the *C. elegans* atypical calpains (true negatives). Application of these profiles to other typical calpain proteins showed that the calpain from Trichoplax is predicted to have a penta EF hand whereas the sponge has only four predicted motifs (Figure 1).

### An abundance of calpain sequences with an incomplete catalytic triad

As indicated above, many *C. elegans* calpain-like proteins are predicted to lack proteolytic activity because the catalytic triad has not been conserved. Such inactive calpains have also been identified in protozoa [14]. In humans, the atypical CAPN6 promotes microtubule stability despite lacking a key residue within the catalytic triad [46]. To examine the prevalence of calpain-like proteins, we selected proteins that were missing at least one catalytic residue by using Fast Statistical Alignment (FSA) to align 1234 proteins from the Uniprot database, which carry the signature calpain catalytic domain SAAS022684_004_001783 [47], [48]. After removing protein fragments and splice variants from this list, a total of 344 calpain-like proteins remained (Table S1). As might be predicted, this analysis successfully identified the *C. elegans*, CLP-3 and CLPR-1 to CLPR-4, Drosophila CALPC and mammalian CAPN6 proteins, which have incomplete catalytic triads. Surprisingly, putative inactive calpain proteins were conserved across the plant, animal and fungus kingdoms (Figure S4A), and were also detected in protists (Figure S4B). Thus, taken together, the abundance and retention of catalytically inactive calpains combined with the finding that CAPN6 is functionally active might argue that these proteins might have hitherto undiscovered functional roles.

### Characterisation of *C. elegans* calpain mutants

Human pathologies, such as LGMD2A, an inherited autosomal-recessive pathology caused by mutations in the typical calpain *CAPN3* gene [25], are associated with calpain dysregulation. To determine whether the atypical calpain proteases participate in physiological and degenerative processes similar to those attributed to typical calpains, we characterized *C. elegans* homozygous mutants carrying deletions within the calpain genes *clp-1*, *-4*, *-6*, *-7*, *-8*, *-9*, *-10* and *clpr-1*; most of the deletions are predicted to disrupt the catalytic domain and to reduce relative mRNA steady-state levels by at least 70% (Table S2). In addition, we examined a *clp-2* mutant carrying a Tc1 transposon inserted within an exon, and performed *clp-3* (*RNAi*) [49]. Phenotypic analysis revealed that brood sizes were not significantly different from wild type for any of the calpain mutants or *clp-3* (*RNAi*) treated animals, except for *clp-10* (*ok2713*) mutants in which the average brood size was reduced by approximately 50% (144±8; n = 4) without a corresponding increase in embryonic lethality. Embryonic lethality was slightly elevated in mutants carrying deletions in calpain genes, but gross developmental, mobility or morphological defects were not observed. For reasons discussed below, we also stained the *clp-1*, *-4*, *-6* and *-7* deletion mutants with phalloidin, but failed to detect disruptions to the sarcomeric structure of adult body wall muscle (Figure S5). These results indicate that most calpain genes, except for *clp-10* and the previously characterized *tra-3/clp-5* sex determining gene [50], play non-essential roles in otherwise wild type animals, although we have not addressed whether these genes could be functionally redundant (Table S2).

### Transcriptional expression profiles of *clp-1* to *clp-7*

The typical *CAPN1* and *CAPN2* calpains are ubiquitously expressed in cells (for review, [1]); however, a temporal elevation in mouse CAPN2 mRNA levels was detected during an essential period of embryonic development [51], [52]. Other typical calpains are capable of displaying more restricted patterns of expression; for example, transcripts corresponding to *CAPN3*, the gene affected in LGMD2A muscular dystrophy, are detected only in skeletal muscle [25]. To gain insights into the potential roles of the *C. elegans* atypical calpain genes based on their expression patterns, we examined transgenic *C. elegans* carrying nuclear-localized mRFP transcriptional reporters driven from promoter regions corresponding to *clp-1* to *-7*. A nuclear localization signal was included to facilitate tissue-specific localization. mRFP was expressed from all of the reporters, except *clp-3* and *clp-6* (Figure S6); only the *clp-2* reporter showed limited expression confined to the intestine (Figure S6). These expression patterns remained unchanged over the course of larval development through to adulthood (Figure S7).

**Figure 2:**
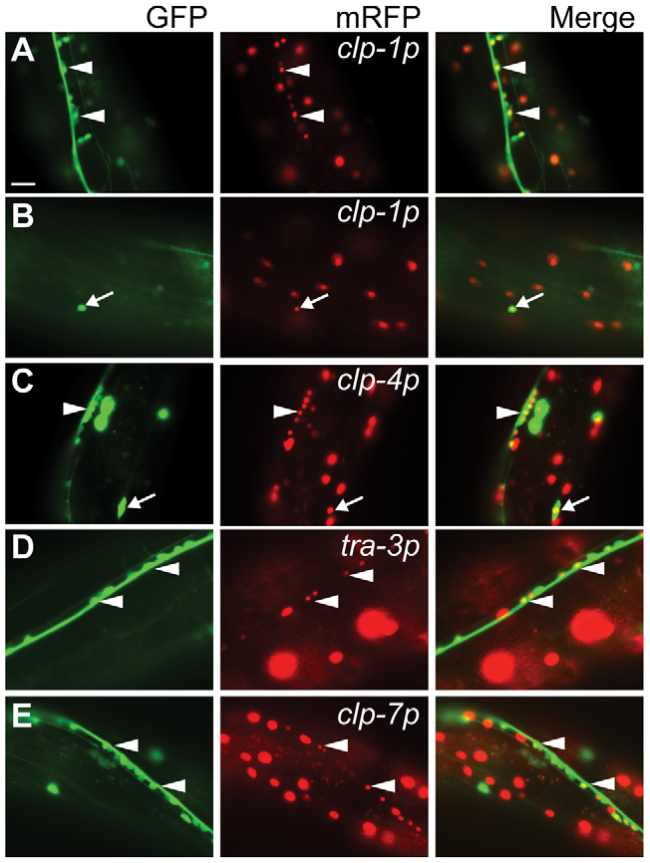
Co-localization of the pan-neuronal *unc-119::gfp* reporter with calpain *nls*-*mrfp* transcriptional reporters. (A) *clp-1p::nls-mrfp* (*crEx65*) is expressed in the ventral and dorsal (B) nerve cord. (C) *clp-4p::nls-mrfp* (*crEx74*) is expressed in the ventral and dorsal nerve cord. (D) *tra-3p::nls-mrfp* (*crEx78*) and (E) *clp-7p::nls-mrfp* (*crEx79*) are each expressed in the ventral nerve cord. Sites of co-localisation between *clp-1p::nls-mrfp*, *clp-4p::nls-mrfp*, *tra-3p::nls-mrfp* or *clp-7p::nls-mrfp* with *unc-119::gfp* expressed in ventral neuronal cell bodies are highlighted with white arrowheads; similarly, sites of co-localisation between *clp-1p::nls-mrfp* or *clp-4p::nls-mrfp* with *unc-119::gfp* expressed in dorsal neuronal cell bodies are highlighted with white arrows. Each micrograph is typical of the pattern observed with at least two other independent transgenic strains generated with the same reporter construct. *unc-119::gfp*, left (green); calpain promoter *nls*-*mrfp*, middle (red); and co-localization, right (yellow). Scale bar is 10 µM.

**Figure 3:**
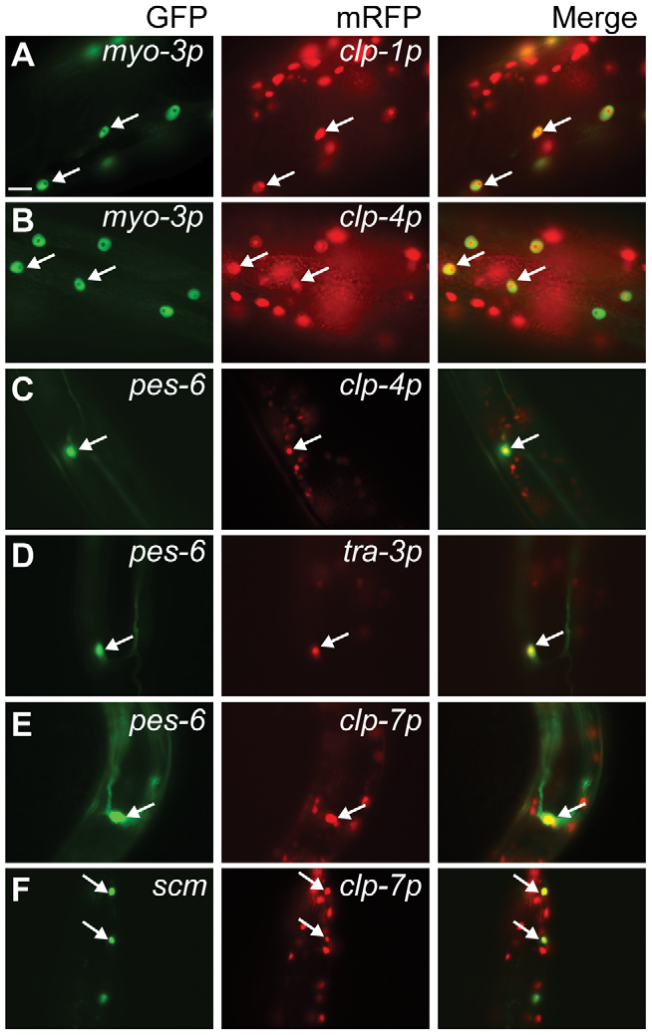
Calpain *nls::mrfp* expression in non-neuronal tissues. Co-localization of the *myo-3p::gfp::nls* body wall muscle reporter with (A) *clp-1p::nls::mrfp* (*crEx65*) and (B) *clp-4p::mrfp* (*crEx74*). Co-localization of the *pes-6::gfp* excretory cell reporter with (C) *clp-4p::nls::mrfp* (*crEx74*), (D) *tra-3p::nls::mrfp* (*crEx78*) and (E) *clp-7p::nls::mrfp* (*crEx79*). (F) Co-localization of the *scm::gfp* seam cell reporter with *clp-7p::nls::mrfp* (*crEx79*). Tissue-specific GFP reporter, left (green), calpain promoter driven *nls::mrfp* expression, middle (red) and co-localization, right (yellow) highlighted with arrows. Each micrograph is typical of the pattern observed with at least two other independent transgenic strains generated with the same reporter construct. Scale bar, 10 µM.

To aid in the identification of tissues displaying calpain gene expression, *clp(p)::nls::mRFP* transcriptional reporters were co-expressed in animals carrying one of five different tissue-specific GFP reporters (for details, see Materials and Methods). We found that the *clp-1*, *-4* and *-7* reporters were active in neurons and co-localized with both the pan-neural *unc-119::gfp* and the GABAergic *unc-47::gfp* reporters, whereas the *tra-3/clp-5* reporter was only detected in non-GABAergic neurons (Figure 2 and Figure S8). In addition, the *clp-1* and *clp-4* reporters were expressed in cells of the ventral and dorsal nerve cords, whereas the *tra-3/clp-5* and *clp-7* reporters were only expressed in cells of the ventral nerve cord (Figure 2 and Figure S8). Given the association between human *CAPN3* and LGMD2A [25], we next co-expressed the *clp* reporters with the body wall muscle marker *myo-3p::gfp::nls* and found that only the *clp-1* and *clp-4* promoters were active in muscle (Figure 3A, 3B).

mRFP expressed from the *clp-4*, *tra-3/clp-5* and *clp-7* gene promoters also co-localized with a *pes-6::gfp* reporter, a marker for the excretory cell, which serves as the renal system of the worm (Figure 3C–3E). In addition, only the *clp-7p::nls::mrfp* transcriptional reporter co-localized with the seam cell reporter *scm::gfp* (Figure 3F). Calpain transcriptional reporters were also detected in other tissues, including the intestine (*clp-1*, *-2*, *-7* and *tra-3/clp-5*), vulva (*clp-1*, *tra-3/clp-5* and *clp-7*) and hypodermis (*tra-3/clp-6* and *clp-7*) (Figure S9). The expression profiles of the *clp-1* to *clp-7* mRFP transcriptional reporters are summarized in Table 1.

**Table 1:** Summary of *C. elegans* calpain transcriptional reporter expression patterns.

	Cell type					
Promoter	Muscle^a^	Neuronal	Intestinal	Hypodermal	Excretory	Seam
*clp-1* b	+	+^c^^,^ d	+	+	−	−
*clp-2*	−	−	+^d^	−	−	−
*clp-3* f	−	−	−	−	−	−
*clp-4*	+	+^c^^,^ d	−	+	+	−
*tra-3/clp-5*	−	+^c^^,^ g	+^h^	+	+	−
*clp-6* f	−	−	−	−	−	−
*clp-7*	−	+^c^	+	+	+	+

a Body wall muscle.

b Expression detected with both transcriptional and translational fusions.

c Ventral nerve cord.

d Dorsal nerve cord.

e Expression is restricted to the intestine.

f No expression detected throughout animal.

g Absent in GABAergic neurons highlighted with *unc-47p*::*gfp* (EG1285).

h Enhanced expression detected in pair of anterior-most intestinal nuclei.

### Ectopic expression of CLP-1 in muscle causes paralysis

Increased calpain activity is associated with degenerative pathologies, such as cataract, neuronal degeneration and muscular dystrophy [25], [53]–[55]. Since a reduction in calpain activity failed to produce readily observable degenerative phenotypes in *C. elegans*, we next investigated the consequences of increasing calpain activity. This was first achieved by ectopically expressing full-length *clp-1*, *-2*, *-4*, *-7* and *tra-3/clp-5* cDNAs under the control of the inducible *hsp-16.41* promoter, which is activated by heat shock in almost all tissues, except the germ line [56]. At least three independent transgenic strains were generated and tested for each construct; however, despite subjecting transgenic animals to daily doses of heat-shock driven calpain expression, we failed to detect abnormal phenotypes in any of the transgenic lines (n>1000 for each strain).

We considered the possibility that the transient nature of heat shock induced gene expression was insufficient for the purpose of eliciting ectopic phenotypes. To test this hypothesis, the constitutively active *unc-54* promoter was used to drive ectopic expression of calpain cDNAs in body wall muscle [57]. Strikingly, we observed that the *unc-54p::clp-1::myc* transgene *crEx325* caused 1.5%±0.4% of adult animals to develop paralysis (n>1000); similar results were obtained for two other independent transgenic lines (data not shown). Affected animals displayed an uncoordinated (Unc) phenotype at the L4/early adult stage, which progressed to paralysis and finally to premature death as animals matured to day 2 adults (Figure S10). This effect was not observed when the other muscle-associated gene, *clp-4*, was constitutively expressed from the *unc-54* promoter, nor when *clp-2*, *-7* or *tra-3/clp-5* cDNAs were similarly expressed.

### CLP-1–induced paralysis is dependent on expression levels and an intact catalytic triad

To understand why the *hsp-16.41p::clp-1* transgene failed to cause paralysis, we compared the levels of CLP-1::MYC expressed from either the muscle-constitutive *crEx325* [*unc-54p::clp-1::myc*] or the heat shock activated *crEx329* [*hsp-16.41p::clp-1::myc*] transgene by western blot analysis (Figure S11A). We also observed that the level of CLP-1::MYC expressed from *crEx329* peaked between 4 to 12 hours post-heat shock before declining (Figure S11B). By comparison, the level of CLP-1::MYC expressed from the *crEx325* transgene was four-fold higher than that observed during the peak of *crEx329* heat shock driven expression. These results indicate that sustained levels of elevated CLP-1 protein promote the development of paralysis.

To establish that an intact catalytic triad was required for CLP-1 to cause paralysis, four independent lines were established that were predicted to express a catalytically inactive CLP-1(C371A) from an *unc-54p::clp-1(C371A)* transgene [58], but none showed mobility defects or paralysis (n>1200). Additional lines expressing mRFP tagged CLP-1 proteins were generated to facilitate western blot analysis. In these lines, paralysis was not detected in *crEx336* [*unc-54p::clp-1(C371A)::mrfp*] animals (n>1200), whereas paralysis developed in 1.9%±0.6% (n>300) of *crEx335* [*unc-54p::clp-1::mrfp*] animals. We also observed that the level of CLP-1::mRFP expressed from *crEx336*, as measured by western blot analysis, was not reduced when compared to *crEx335*, and so could not account for the difference in their activities (Figure S11C). Attempts were also made to demonstrate CLP-1 proteolytic activity using casein zymography and the calpain-GLO protease™ assay (Promega), which are used to measure typical calpain activity [59]. Unfortunately, neither casein nor suc-LLVY-aminoluciferin was found to be a suitable substrate for CLP-1, although a similarly prepared recombinant rat CAPN2 was active in both assays.

### Ectopic expression of *clp-1* in neurons fails to elicit neurodegenerative or neuromuscular defects

We next examined *clp-1* activity in neurons. Previous studies have shown that RNAi inactivation of *clp-1* partially suppressed the degeneration of touch receptor neurons in animals carrying gain-of-function mutations in the *mec-4* or *deg-1* Na^+^ channel subunits [35], although degeneration was not observed when *clp-1* was overexpressed in touch receptor neurons [35]. We also tested and failed to observe neurodegenerative phenotypes when either the *unc-119p::clp-1* or *unc-47::clp-1* reporter was expressed in other neurons.

### Ectopic expression of *clp-1* causes muscle cell abnormalities

**Figure 4:**
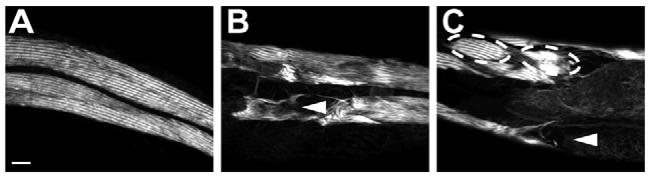
Ectopic *crIs4* [*unc-54p::clp-1*] expression disrupts body wall muscle. (A) Typical pattern of muscle striations in phenotypically wildtype *crIs4* adults. (B) Unc *crIs4* animal showing loss of actin striations (arrowhead). (C) Paralyzed *crIs4* animal with bundled muscle cells (dashed ring) and missing muscle cells (arrowhead). Scale bar, 10 µm.

In N2 wild type animals, body wall muscle cells form striated diamond shaped bundles that are arranged into four quadrants running down the length of the animal [60]. We hypothesized that constitutively elevated expression of CLP-1 in body wall muscle cells was causing extensive myofibrillar damage, which in turn was leading to paralysis. Before examining muscle morphology, we chromosomally integrated the *crEx325* [*unc-54p::clp-1*] array to generate *crIs4*, in order to circumvent potential problems associated with mosaic expression [61]. We found that the integrated *crIs4* array retained the same phenotypic characteristics as *crEx325*; 1.69±0.34 (n = 352) of *crIs4* animals displayed paralysis. We next synchronized the growth of *crIs4* worms and separated *crIs4* adults into three distinct classes: 1) phenotypically wildtype with normal sinusoidal movement 2) Unc and 3) paralyzed, and examined the integrity of body wall muscles by staining actin thin filaments with phalloidin. We found that phenotypically wildtype *crIs4* animals exhibited only occasional muscle cell abnormalities (Figure 4A, Table 2), whereas Unc *crIs4* animals displayed disorganized bundles of actin fibers and were also missing body wall muscle cells (Figure 4B, Table 2). These abnormalities were even more extensive in paralyzed *crIs4* animals (Figure 4C, Table 2). Thus, the paralysis observed in *crIs4* animals can be attributed to the loss of sarcomere integrity.

**Table 2:** Deletion of *C. elegans clp-1* partially suppresses dystrophin-based muscle degeneration.

Genotype	Abnormal body wall muscle cells^a^
*crIs4* wildtype-like	0.2±0.1
*crIs4* Unc	11.0±1.0
*crIs4* paralysed	33.8±0.6
*clp-1(tm690)*	0.0±0
*dys-1(cx18);hlh-1(cc561ts)*	10.9±0.4
*dys-1(cx18);hlh-1(cc561ts); clp-1(tm690)*	5.9±0.6^b^

a Worms were grown at 15°C, and scored as Day 2 adults. Following phalloidin staining, the 20 most central muscle cells from each of the two most visible body wall muscle quadrants were scored (40 cells per animal), as described in Gieseler et al. (2000). Number represents the mean ± standard deviation from 3 independent experiments involving at least 30 animals per experiment.

b Reduction is significantly different based from *dys-1(cx18); hlh-1(cc561ts)* based on Student's t-test (P<0.001).

### CLP-1 localizes to muscle M-lines and adhesion plaques

**Figure 5:**
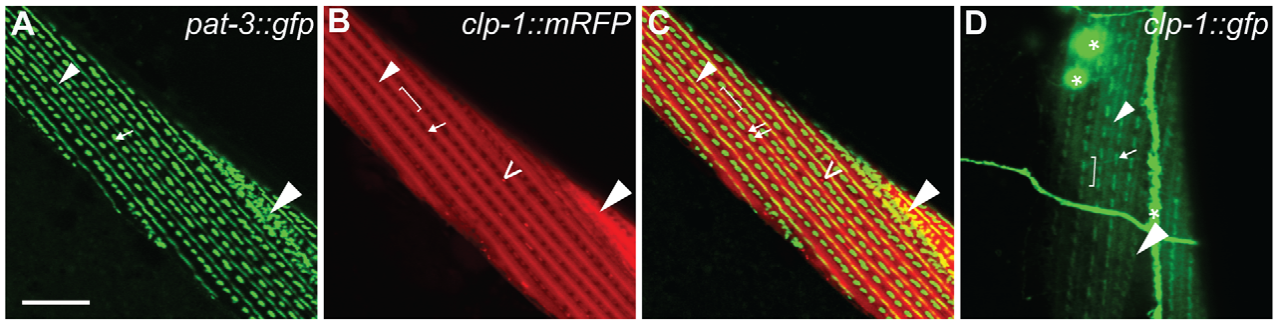
Ectopic *unc-54p::clp-1::mrfp* localizes to M-lines and adhesions plaques in body wall muscle. (A) *pat-3p::gfp* integrin reporter (green) localizes to the M-lines, dense bodies and adhesion plaques. (B) *unc-54::clp-1::mrfp* (red) is expressed in sarcomeres and forms aggregates. (C) Co-localization of *unc-54::clp-1::mrfp* (red) (B) with the *pat-3p::gfp* integrin reporter (green) (A) at the M-line extending over the H-zone and adhesion plaques. *unc-54::clp-1::mrfp* does not co-localize at dense bodies. (D) *clp-1::gfp* shows a similar pattern of expression as (B), but is also expressed in neuronal structures marked with an asterisk. M-line, small arrowhead; H-zone, bracket; dense body (Z-disk), arrow; aggregates, open arrowhead; adhesion plaques, large arrowhead. Scale bar is 10 µm.

Given that CLP-1 promotes the degeneration of body wall muscle, we examined the intracellular localisation of CLP-1::mRFP in *qyIs43* animals co-expressing a beta-integrin subunit [*pat-3::gfp*] (Figure 5) [62]; in muscle cells, PAT-3 is found at the base of thick filament M-lines, dense bodies (Z-disks) and in adhesion plaques that are formed between adjacent cells (Figure 5A). We found that CLP-1::mRFP was excluded from the nucleus (not shown) and dense bodies, but was present at structures immediately adjacent to dense bodies (Figure 5B). More specifically, CLP-1::mRFP co-localized with PAT-3::GFP at M-lines extending over the H-zone and at adhesion plaques (Figure 5C). We also generated a native *clp-1::gfp* translational reporter, which confirmed that CLP-1::GFP displayed the same pattern of sarcomeric localization, as CLP-1::mRFP driven from the *unc-54* promoter (Figure 5D and Figure S12); CLP-1::GFP was also detected in other non-muscle tissues. Aggregates of CLP-1::mRFP were also observed, which might contribute to muscle degeneration and paralysis, although we cannot exclude the possibility that aggregation is an artifact of overexpression (Figure 5B). Thus, we speculate that sarcomeric proteins enriched at the sites of CLP-1 localization are potential targets for degradation.

### Reduction of CLP-1 activity suppresses muscle degeneration in a *C. elegans* model of muscular dystrophy

In mammals, the absence of the large structural muscle protein dystrophin underlies the muscle degenerative disorder Duchenne muscular dystrophy (DMD) [63]. Similarly, a *C. elegans* DMD model is based on the progressive muscle degeneration observed in *dys-1*(*cx18*); *hlh-1*(*cc561ts*) animals [60]. In *C. elegans*, a null mutation in the only dystrophin-like protein gene, *dys-1*(*cx18*), caused only occasional muscle degeneration [64]. However, inclusion of a temperature sensitive allele of the MyoD transcription factor homolog, *hlh-1(cc561ts)*, sensitized *dys-1*(*cx18*); *hlh-1*(*cc561ts*) mutants to become uncoordinated (Unc), but not paralyzed, and to display increased muscle degeneration [60].

We investigated whether CLP-1 contributed to the muscle degeneration associated with *C. elegans* DMD by constructing the *dys-1*(*cx18*); *hlh-1*(*cc561ts*); *clp-1*(*tm690*) strain. The *clp-1*(*tm690*) mutation deletes a 624 bp region of the *clp-1* gene and introduces a translational frame-shift that is predicted to produce a 493 amino acid truncated protein, which lacks two of the three critical catalytic residues required for proteolytic activity. *clp-1* (*tm690*) mutants are phenotypically wild-type and do not display any obvious defects in their muscle structure (Table 2, Figure S5). When the number of abnormal muscle cells present in *dys-1*(*cx18*); *hlh-1*(*cc561ts*); *clp-1*(*tm690*) was compared to that found in *dys-1*(*cx18*); *hlh-1*(*cc561ts*) animals after phalloidin staining, we found that the absence of *clp-1* reduced the number of abnormal muscle cells by almost 50% (p<0.001). A body wall muscle cell was scored as abnormal when: 1) the classic striated pattern of actin filaments was disrupted 2) actin bundles were visible as puncta or 3) muscle cells were missing due to cell death. Thus, *clp-1* is normally active in muscle and contributes to the muscle degeneration observed in *dys-1*(*cx18*); *hlh-1*(*cc561ts*) animals (Table 2).

### Genetic manipulation of [Ca^2+^]_i_ levels exacerbates *clp-1*–induced paralysis

Although ectopic expression of CLP-1 led to a degradative muscle pathology, we were surprised that the penetrance of the effect was not higher. To ask what other factors might modulate *clp-1* activity in muscle, we first took a genetic approach to investigate the effect of intracellular calcium [Ca^2+^]_i_. Although calpains are referred to as Ca^2+^-activated proteases, little is known about the effect of physiological calcium levels on the activity of atypical calpains. In *C. elegans*, four allelic modifiers have been identified that are understood to increase [Ca^2+^]_i_: *egl-19*(*ad695gf*), *itr-1*(*sy290gf*), *slo-1*(*js379*) and *unc-24*(*e138*) (for descriptions of mutants, see Materials and Methods). We generated strains carrying each of these mutations in combination with *crIs4* and scored adults for paralysis.

**Figure 6:**
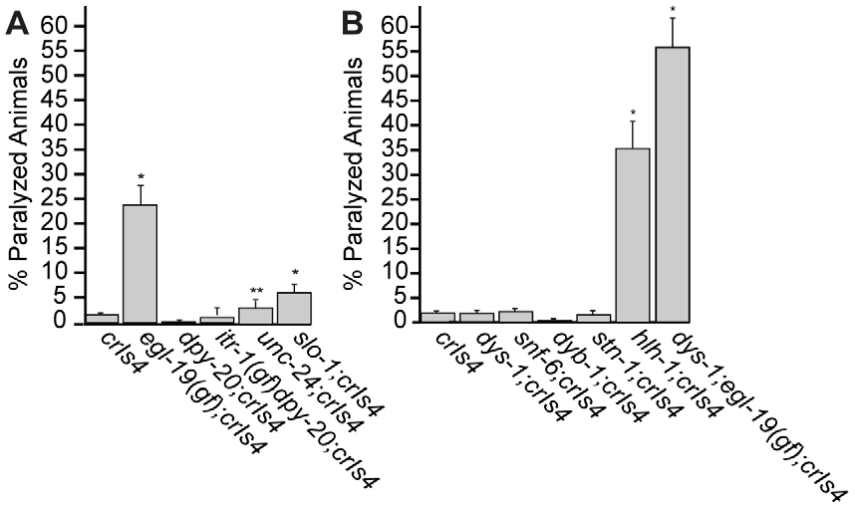
Genetic enhancers of *crIs4* [*unc-54p::clp-1*] induced paralysis. (A) Genetic mutations in calcium channel genes that are predicted to increase [Ca^2+^]_i_ enhance *crIs4* [*unc-54p::clp-1*] induced paralysis. (B) DAPC mutants do not enhance *crIs4* [*unc-54p::clp-1*] induced paralysis unless combined with a mutation that also increases [Ca^2+^]_i_. Percentages represent the mean ± SEM from at least 4 independent experiments, which involved counting day 2 adults from synchronized worm populations (see Materials and Methods). For each experiment an n>120 was used, except for the strains *crIs4* and *dys-1(cx18); egl-19(ad695); crIs4* where an n>60 was used. In the absence of *crIs4*, none of the mutant genotypes led to paralysis (n>100, for each strain). *unc-24* mutants are Unc, but not paralyzed. Significance of difference by comparison to *crIs4* is based on Student's T-test (*, P<0.001) and (**, P = 0.043).

We found that the number of animals displaying paralysis was significantly increased when *crIs4* was combined with mutations in *egl-19*(*ad695*), *unc-24*(*e138*) *or slo-1*(*js379*) (Figure 6A). Inclusion of the *egl-19*(*ad695gf*) mutation produced the most dramatic increase in paralysis (p<0.001), whereas the *unc-24*(*e138*) mutation caused a mild, but significant increase (p = 0.043) (Figure 6A). Although the *slo-1*(*js379*) mutation also increased the level of *crIs4* paralysis, this effect could be attributed to either its role in Ca^2+^ regulation or to its participation in the Dystrophin Associated Protein Complex (DAPC), discussed below [65]. The *itr-1(sy290gf)* mutation did not significantly affect the number of paralyzed *crIs4* animals; however, ITR-1 expression has not been detected in muscle [66].

### Genetic defects in the Dystrophin Associated Protein Complex (DAPC) do not enhance *clp-1*–induced paralysis

We next constructed a set of double mutants between *crIs4* and members of a second group of allelic modifiers that form the Dystrophin Associated Protein Complex (DAPC): *dys-1*(*cx18*), *snf-6*(*ok720*), *dyb-1*(*cx36*) and *stn-1*(*ok292*), which encode a dystrophin-like protein, acetylcholine transporter, dystrobrevin and syntrophin, respectively [64], [67]–[69]. Constituents of the DAPC provide structural support to muscle by linking the cytoskeleton to the sarcolemma and extracellular matrix [70]. We hypothesized that mutations capable of destabilizing the integrity of the DAPC could sensitize muscle cells to CLP-1 induced damage. The four mutants listed above and *slo-1*(*js379*) share a similar phenotype marked by hyperactivity and exaggerated head bending [64], [65], [67]–[69]. An *hlh-1*(*cc561ts*) temperature sensitive allele of a MyoD homolog was also examined because this mutation sensitizes dystrophin *dys-1*(*cx18*) mutants to muscle damage [60].

We found that disrupting the structural proteins of the DAPC complex did not significantly sensitize animals to the effects of ectopic *clp-1* provided by *crIs4* (Figure 6B). By contrast, inclusion of the *hlh-1*(*cc561ts*) allele substantially increased the proportion of paralyzed *crIs4* animals. We next tested whether the effects of *crIs4* could be further enhanced in a *dys-1*(*cx18*); *egl-19*(*ad695gf*) genetic background by increasing [Ca^2+^]_i_. Adult *dys-1*(*cx18*); *egl-19*(*ad695gf*) double mutants in the absence of *crIs4* are Unc, but not paralyzed, and exhibit moderate muscle degeneration [71]. We found that the percentage of *dys-1*(*cx18*); *egl-19*(*ad695gf*); *crIs4* adults developing paralysis was vastly elevated (56.3±6.4%) when compared to animals expressing *crIs4* in combination with a mutation in either *dys-1*(*cx18*) or *egl-19*(*ad695gf*) alone (Figure 6B). Moreover, the brood size of *dys-1*(*cx18*); *egl-19*(*ad695gf*); *crIs4* animals was dramatically reduced and embryonic lethality elevated (Table S3).

The heightened sensitivity of *dys-1*(*cx18*); *egl-19*(*ad695gf*) mutants to *crIs4* induced paralysis led us to investigate whether *dys-1*(*cx18*); *egl-19*(*ad695gf*) mutants might also be sensitized to the effects of ectopic *clp-2*, *-4*, *-7* or *tra-3/clp-*5 expression in muscle under control of the *unc-54* promoter. We examined over 1000 animals from at least three independent transgenic lines for each calpain, however, we failed to detect any exacerbated defects in movement aside from those already reported for *dys-1*(*cx18*); *egl-19*(*ad695gf*) mutants [71]. Hence, of the calpains tested, only *clp-1* was found to induce paralysis when overexpressed in muscle under conditions of elevated [Ca^2+^]_i_.

### Inhibition of the *egl-19* channel reduces *clp-1*–induced paralysis

**Figure 7:**
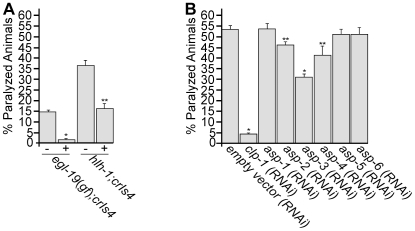
Small molecule and genetic inhibition of *crIs4* [*unc-54p::clp-1*] induced paralysis. (A) Treatment with 5 µM nemadipine-A reduced *crIs4* [*unc-54p::clp-1*] induced paralysis in *egl-19*(*ad695*); *crIs4* and *hlh-1*(*cc561ts*); *crIs4* animals. % paralyzed animals for each strain are indicated with (+) or without (−) nemadipine-A treatment. Significance of difference based on Student's T-test compared to *egl-19*(*ad695*); *crIs4* (*, P<0.001) or *hlh-1*(*cc561ts*); *crIs4* (**, P = 0.001) without nemadipine-A treatment. (B) Treatment of *hlh-1*(*cc561ts*); *crIs4* animals with empty RNAi feeding vector (L4440), *clp-1*, or *asp-1* to *asp-6* (*RNAi*). Significance of difference compared to *hlh-1*(*cc561ts*); *crIs4* fed on empty vector (*RNAi*) is based on Student's T-test (*, P<0.001; **, P<0.05). Numbers shown represent the mean ± SEM from at least 5 independent experiments, which involved counting day 2 adults from synchronized worm populations (see Methods). n>60 per experiment.

The EGL-19 L-type voltage gated Ca^2+^ channel is located along the basal membrane of muscle. To demonstrate that *egl-19(gf)* was exerting an effect on *clp-1* activity specifically by altering [Ca^2+^]_I_, we asked if the small molecule antagonist, nemadipine-A, could suppress CLP-1 mediated muscle degeneration; nemadipine-A has been shown to be a specific and highly effective inhibitor of EGL-19 [72]. We found that a dose of 5 µM nemadipine-A was sufficient to abolish the enhanced level of paralysis observed in *egl-19*(*ad695*); *crIs4* mutants (Figure 7A). We next treated *hlh-1*(*cc561ts*); *crIs4* mutants with nemadipine-A and found that paralysis was also reduced by over 50% compared to untreated animals (Figure 7A). This result indicates that the *hlh-1*(*cc561ts*) mutation is likely to sensitize muscle cells to the effects of *crIs4* indirectly by causing an increase in [Ca^2+^]_I_, which can be suppressed by inhibiting the activity of the EGL-19 Ca^2+^ channel.

### RNAi inactivation of aspartyl proteases suppresses *clp-1*–induced paralysis

The aspartyl proteases, *asp-1*, *asp-3* and *asp-4*, have previously been shown to be constituents of a neural degenerative pathway involving the calpains *clp-1* and *tra-3*/*clp-5* [35]. To ask if the *asp* genes also promote muscle degeneration, we performed RNAi with the genes *asp-1* to *asp-6* on *hlh-1*(*cc561ts*); *crIs4* animals. The effectiveness of RNAi knockdown was measured by quantitative PCR (qPCR) (Table S4). We found that *asp-3*(*RNAi*) reduced paralysis levels by over 40% and that *asp-2(RNAi)* and *asp-4*(*RNAi*) each reduced paralysis by ∼15%, whereas *asp-1*(*RNAi*) had no measurable effect in muscle. We further showed that *clp-1*(*RNAi*) almost completely abolished *crIs4* induced paralysis in *hlh-1*(*cc561ts*); *crIs4* animals, as might be expected (Figure 7B).

## Discussion

### Typical and atypical calpains are both detected in ancient metazoan phyla

CAPN1 and CAPN2 are still referred to as the main or major calpains despite the rich variety and abundance of genes encoding atypical calpain proteins (Figure 1). However, phylogenetic analyses performed here and by others indicate that the C-terminal EF hand motifs in typical calpains are unlikely to represent embellishments acquired late in metazoan evolution, as might be inferred by the greater importance attached to typical calpains [1], [14]. We detected typical calpain homologs in basal metazoan phyla, such as Nematostella, Trichoplax and Hydra [39]–[41], as well as in sponge, a representative of an early divergent metazoan clade [42], and found that the EF hand domain in early metazoan typical calpain proteins was already composed of 5 motifs (PEF) (Figure 1). The absence of typical calpain genes in *C. elegans* and the presence of both typical and atypical calpain genes in Schistosomes and Drosophila further suggests that *C. elegans* is likely to have undergone a lineage-specific loss of typical calpain genes; it is noteworthy that Drosophila and *C. elegans* have both been assigned to the clade Ecdysozoa [73]. A similar line of reasoning could also be used to explain the absence of typical calpain genes in the genome of the ascidian Ciona [43].

Studies directed toward understanding the function and regulation of atypical calpains have lagged, or have possibly been confounded by the presence of typical calpains. *C. elegans* presents an ideal model in which to examine the function of atypical calpains, because their genome encodes a range of variants that are representative of those found across phyla, including ancient metazoan lineages, and because of the availability of mutant alleles (Figure S1). Our analyses of these mutants have shown that *clp-10(ok2713)* mutants have a reduced brood size, but that the remaining mutants, with the exception of *tra-3/clp-5*, do not display obvious phenotypes affecting viability, motility or fertility [50]. Seven of the nine characterized mutants are represented by null or strong loss-of-function alleles that have eliminated or disrupted the catalytic triad; qPCR also shows that levels of affected transcripts were reduced in mutants by at least 70% (Table S2). Despite our inability to detect single mutant phenotypes, our results demonstrated that deletion of *clp-1* suppressed muscle degeneration in *dys-1*(*cx18*); *hlh-1*(*cc561ts*) mutants (Table 2). In neurons, it was similarly shown that RNAi knockdown of either *clp-1* or *tra-3/clp-5* suppressed touch cell degeneration in mutants carrying a dominant-gain-of-function *mec-4* allele [35]. These results indicate that the ability to detect phenotypes in atypical calpains might be dependent on the genetic background or physiological state of the animal. It also remains possible that the expansion in the family of *C. elegans* atypical calpain genes has made it difficult to detect single mutant phenotypes because of functional redundancy.

### Regulation and specificity of atypical calpain activity

To gain insights into the physiological roles of atypical calpain proteins, we examined the consequences of calpain overexpression, but were unable to detect any obvious phenotypic changes resulting from heat-shock driven overexpression. CLP-1 protein expressed under these conditions peaked 4–8 hours post heat-shock before declining; by contrast, the level of CLP-1 protein resulting from *unc-54* promoter activity was shown to be comparable to that detected during the peak period of expression after heat shock (Figure S11). The near-absence of phenotypes resulting from calpain overexpression could also indicate that enhanced atypical calpain activity is not necessarily detrimental in a healthy cell. One could speculate that if a given atypical calpain were constitutively active, then overexpression might have limited effect. For example, although heat shock driven expression of a wildtype *tra-3/clp-5* transgene was clearly sufficient to prevent the sexual transformation of a *tra-3/clp-5* null mutant, rescued animals did not display any degenerative phenotypes [34].

The substrate specificity of typical calpains is determined not only by primary sequence, but also by higher order structural features [30]. Very little is known about the substrate requirements for atypical calpains, but in our hands, CLP-1 and CLP-7 failed to cleave standard typical calpain substrates in vitro (data not shown). Our experiments also revealed that of all the *clp* genes tested only *clp-1* driven from the *unc-54* promoter led to muscle degeneration and paralysis (Figure S10). The inability of the other atypical calpains to cause paralysis would indicate that sarcomeric proteins are either not general substrates for atypical calpains or that they are inaccessible unless damaged. A third possibility is that an intracellular inhibitor regulates the activities of atypical calpains. In mammals, calpastatin directly inhibits CAPN1 and CAPN2 under pathological conditions [74]. The *C. elegans* genome lacks a calpastatin gene, but an intracellular serpin protease inhibitor, SRP-6, has been hypothesized to inhibit TRA-3/CLP-5 and CLP-10 in the intestine in response to hypo-osmotic shock [37]. A family of *srp* genes has been identified, so interactions with other *clp* genes might remain to be uncovered.

### Calcium activation of atypical calpains

Typical calpain proteases require Ca^2+^ for activity, but under conditions of reduced [Ca^2+^]_i_, they can be stimulated through the interaction of the C2 domain with membrane phospholipids and through the autolysis of DI [6], [7], [15]. So, how does calcium influence atypical calpain activity? We previously showed that TRA-3/CLP-5 undergoes calcium-dependent autolysis [34], but it remains unknown if any of the other atypical calpains can undergo this process, or if autolysis enhances proteolytic activity. Structural analyses of a mini-calpain identified two Ca^2+^ binding sites within IIa and IIb of CAPN2 DII, which help to align the catalytic triad when Ca^2+^ is bound [8]. Extrapolating from this model, the conservation of key residues in DII would predict that atypical calpains would display a similar Ca^2+^ dependency (Figure S2). The potential influence of phospholipids on the activity of atypical calpains has yet to be examined, although DIII forms a C2 fold that could interact with Ca^2+^ and phospholipids [75].

It is clear that the physiological rise in [Ca^2+^]_i_ achieved through the use of genetic mutants profoundly affected CLP-1 activity (Figure 6A). However, it is important to emphasize that it was the synergistic increase of both CLP-1 and [Ca^2+^]_i_ levels that contributed to paralysis. None of the Ca^2+^ channel mutants examined developed paralysis, indicating that an increase in [Ca^2+^]_i_ by itself is unable to pathologically activate *clp-1*. Nonetheless, removal of *clp-1* substantially suppressed the muscle degeneration associated with *C. elegans dys-1*; *hlh-1*(*cc561ts*) DMD, which is based on a mouse model of myopathy involving the combined mutation of MyoD and dystrophin (Table 2) [60], [76]. In muscle degenerative conditions, such as DMD, a rise in [Ca^2+^]_i_ is also hypothesized to activate calpain proteolysis [77]–[79].

We have also shown that the *C. elegans hlh-1*(*cc561ts*) allele of a MyoD homolog, sensitizes *C. elegans* to the effects of *crIs4*, as measured by the increase in paralysis, and that treatment of these worms with nemadine-A suppressed paralysis (Figure 7A). As nemadipine-A is a specific inhibitor of the EGL-19 L-type Ca^2+^ channel [72], it was not surprising to find that this drug also suppressed the paralysis associated with *egl-19*(*gf*); *crIs4* mutants. Based on these results, we propose that the *hlh-1*(*cc561ts*) mutation creates a sensitized background for calpain activity by indirectly elevating [Ca^2+^]_I_, although it remains unclear if EGL-19 activity is disrupted. Following this logic, we speculate that inappropriate CLP-1 activity in a sensitized background could generate a positive feedback loop whereby calpain disrupts Ca^2+^ channel activity, leading to increased [Ca^2+^]_i_, and further activation of CLP-1. In support of this model, several studies have shown that typical calpains can cleave Ca^2+^ channels and disrupt their activities [80]–[82].

### Physiological regulation of calpains in muscle

In mammals, inappropriate elevation of either CAPN1 or CAPN2 is associated with muscle degeneration [54]. Indirect evidence has also accumulated pointing to the involvement of these calpains and the skeletal muscle specific CAPN3 isoform in myofibrillar protein turnover, which promotes the replacement of damaged sarcomeric components in order to maintain efficient muscle contraction [7], [25], [83]–[88]. What roles do atypical calpains normally play in muscle? In our study and in reports by others, only the *clp-1* and *clp-4* genes appear to be expressed in muscle (Figure 3) [35], [89], and it was further found that a rescuing *tra-3/clp-5::gfp* translational fusion was not expressed in muscle [90]. Surprisingly, it has recently been reported that chronic RNAi knockdown of *clp-1*, *clp-4*, *tra-3*/*clp-5*, *clp-6* or *clp-7* was responsible for causing myofilament disruption [91], suggesting that calpains are involved in myofilament maintenance. By contrast, we failed to detect sarcomeric abnormalities in similarly staged adults when phalloidin was used to examine the body wall muscle of animals carrying deletion alleles in *clp-1*, *clp-4*, *clp-6* or *clp-7* (Figure S5). We are unable to account for these differences, although it has been reported elsewhere that a GFP-tagged myosin heavy chain reporter [*myo-3::gfp*] can independently cause an age-dependent sarcopenia in adults [92]. Nonetheless, our study independently supports a role for CLP-1 in the maintenance of muscle adhesion complexes and turnover of myofibrillar proteins.

We have obtained evidence that CLP-1 has the potential to disrupt sarcomeric integrity, indicating that CLP-1 is likely to target components of muscle adhesion complexes for destruction (Figure 4 and Figure 6). The localization of CLP-1 to M-lines and to structures immediately adjacent to Z-disks shows that CLP-1 is well positioned to participate in myofibrillar turnover or possibly remodelling of integrin-based muscle attachment assemblies (Figure 5); however, it is interesting that CLP-1 is excluded from dense bodies (Z-disks). CLP-1 could also regulate muscle cell-muscle cell interactions through its localization to adhesion plaques. In *C. elegans*, studies based on the use of FRAP show that *C. elegans* sarcomeric proteins undergo dynamic exchange suggestive of protein turnover [93]. Taken together, these observations suggest that CLP-1 might normally promote myofibrillar protein turnover and help to maintain the ordered alignment of adhesion complexes, or to accommodate changes to the sarcomere due to growth or cell damage. Evidence has also been obtained from mammalian systems indicating that the typical CAPN1 and CAPN2 calpains are able to regulate the dynamics of cell adhesion complexes [18], which are similar in composition to those found in *C. elegans* muscle [19].

Our data did not support the expectation that disruptions to the DAPC, an important complex that maintains muscle structural integrity in humans, would synergize with CLP-1 overexpression and lead to increased sarcomeric damage and paralysis in worms (Figure 6B) [70]. However, it has been reported that the *C. elegans* DAPC promotes, but is not essential for muscle integrity [64], [69], [94], [95], so destabilisation of the complex might not be sufficient to induce damage and heightened myofibril turnover, despite the increased availability of CLP-1.

### A conserved calpain–cathepsin pathway of muscle degeneration

We propose that the death of muscle cells observed in paralyzed *crIs4* animals is caused by activation of a pathway promoting necrosis. In mammalian neurons, a model has been proposed whereby inappropriate increases in [Ca^2+^]_i_ caused by insults such as ischemia/reperfusion injury contribute to the activation of calpains and lead to the permeabilization of lysosomes. In turn, lysosomal rupture allows cathepsin proteases to leak into the cell and cause widespread degradation and necrotic cell death [55]. In *C. elegans*, support for this model was obtained when it was shown that *clp-1*, the aspartyl proteases *asp-3* and *asp-4* and to a lesser extent *asp-1* were required for the necrotic death of neuronal touch receptors and vulval uv1 cells [35], [36]. Similarly, our data also revealed the importance of *asp-3* in promoting *clp-1* mediated paralysis in muscle (Figure 7B). By contrast to studies in neurons, we found that *asp-4* has a reduced role in muscle degeneration and that *asp-1* doesn't appear to be active in muscle, although we obtained evidence that *asp-2* participates in this process. We, therefore, propose that a calpain-cathepsin pathway of degeneration has been conserved in *C. elegans* muscle.

Finally, the ability of nemadipine-A to reduce the level of paralysis in *hlh-1; crIs4* worms shows that physiological conditions that lead to an indirect increase in [Ca^2+^]_i_ can have a destructive and possibly necrotic outcome in muscle cells by activating atypical calpain activity (Figure 7A) [96]. Therefore, it will be of further interest to identify physiological conditions that could lead to elevated calpain expression or [Ca^2+^]_i_ as causative factors in sarcopenia, a loss in muscle contractility observed in ageing cells [97], [98]. We propose that the identification of atypical calpain inhibitors would represent a fruitful area for pharmacological investigation. It should be possible to take advantage of the paralytic phenotype displayed by *hlh-1(cc561ts)*; *crIs4* mutants and to perform a chemical screen to identify compounds that ameliorate muscle damage. Such a screen could potentially identify calcium channel blockers or specific inhibitors of atypical calpains. Given that atypical calpain genes are also present in mammals, our results emphasize the importance of investigating the contribution of atypical calpains to degenerative disorders, especially under conditions associated with inappropriately elevated [Ca]_I_, such as muscular dystrophies, neurodegeneration and cataract.

## Materials and Methods

### Blast analyses and protein phylogeny

See Protocol S1 for sequence accession numbers and details about sequence similarity searches and protein alignments.

A custom profile hidden Markov model was created for each of the five EF-hand motifs of the penta EF hand, using the following proteins as a training set: HsCAPN1 (AAH75862.1), HsCAPN2 (NP_001739.2), HsCAPN3/p94 (AAI46650.1), HsCAPN8 (NP_001137434.1), HsCAPN9 (NP_006606.1), HsCAPN11 (EAX04252.1), DmCALPA (NP_001097378.1), DmCALPB (NP_524016.4), DmCALPC (AAF48591.2) and HsGRAN (P28676) [45]. These full-length proteins were aligned using FSA [48], and each of the EF hand motifs was sliced out of the alignment, based on the coordinates provided for the EF hand motifs of HsCAPN1, to generate five multiple sequence alignment files (MSF) [99]. The hmmbuild program in the HMMER software package was then used to create a profile HMM for each of the five EF-hand motifs [45]; pfam HMM files are available as Text S1. The EF-hand motif model was validated using true positive penta-EF hand domain containing proteins CAPNS1 (AAH64998.1) and Sorcin (AAA92155.1); HsCAPN10 and the *C. elegans* atypical calpains were shown to be true negatives. An E-value cutoff level of 0.01 was used.

### *C. elegans* methods

Worms were grown and maintained at 20°C as described [100], except strains containing *hlh-1(cc561ts)* II, which were grown at 15°C [101]. The following strains were used: wild-type Bristol N2, HC46: *ccIs4215* [*myo-3::gfp::nls*] I, LS292: *dys-1*(*cx18*) I, *clp-8* [*F44F1.3*] (*ok1878*) I, *clp-9* [*T11A5.6*] (*ok1866*) I, *clpr-1* [*W04A4.4*] (*ok2601*) I, LS505: *dyb-1*(*cx36*) I, LS721: *stn-1*(*ok292*) I, LS587: *dys-1*(*cx18*) I; *hlh-1*(*cc561ts*) II, LS706: *dys-1*(*cx18*) I; *egl-19*(*ad695gf*) IV, NL2099: *rrf-3*(*pk1426*) II, PD4605: *hlh-1(cc561ts)* II, *clp-1*(*tm690*) III, *clp-2* (*pk323*) III, *clp-4* (*ok2808*) III, *clp*-10 [W05G11.*4*] (*ok2713*) III, JR667: *unc-119* (*e2498*::Tc1) III, VC591: *okIs53 snf-6*(*ok720*) III, *dpy-20(e1282)* IV, DA695: *egl-19*(*ad695gf*) IV, *clp-6* (*ok1779*) IV, *clp-7* (*ok2750*) IV, *itr-1(sy290) dpy-20(e1282)* IV, *unc-24*(*e138*) IV, NM1968: *slo-1*(*js379*) V, EG1285: *lin-15*(*n765*); *oxIs12* [*unc-47::gfp*+*lin-15*(+)] X, IM19: *urIs13* [*unc-119::gfp* (IM#175; *rol-6*(*su1006*)], NK358: *unc-119*(*ed4*) III; *qyIs43*, UG756: *bgIs312* (*pes-6::gfp*), *wIs51* [*scm::gfp* (seam cell)+*unc-119*(+)]. The *clp-1*(*tm690*) III allele (kindly provided by Shohei Mitani) was sequenced using primers GGATGAGCTCTTCTATCGTG and GTCTGACCATGGTCCATTCC to confirm the presence of a 624 bp deletion, which is predicted to create a frame-shift at A(460), and produce a truncated protein of 493 amino acids.

*egl-19* encodes an L-type voltage gated Ca^2+^ channel; an *egl-19*(*ad695gf*) gain-of-function mutation delays the inactivation of the EGL-19 L-type voltage gated Ca^2+^ channel, and hence leads to increased [Ca^2+^]_i_ [102]. The *itr-1* gene encodes an inositol 1,4,5-triphosphate receptor (IP3R) homolog that resides on the endoplasmic reticulum (ER) and releases Ca^2+^ into the cytoplasm in response to IP3 signalling [103]; the *itr-1(sy290gf)* allele is a gain-of-function mutation that increases [Ca^2+^]_i_. Because the *itr-1(sy290gf)* mutant carries a point mutation and does not display an obvious phenotype, the *dpy-20*(*e1282*) mutation, which is closely linked to the *itr-1* gene, was included to facilitate the identification of *itr-1* homozygotes. To account for potential marker effects, paralysis was also scored in a *dpy-20*(*e1282*); *crIs4* genetic background. *slo-1*(*js379*) is a loss-of-function allele of a gene that encodes a Ca^2+^ activated potassium BK channel [104]. The SLO-1 channel is normally activated by a rise in [Ca^2+^]_i_, which leads to plasma membrane hyperpolarization. In turn, [Ca^2+^]_i_ is reduced by the inactivation of L-type Ca^2+^ channels, such as EGL-19 [65], [104], [105]. *unc-24*(*e138*) is a loss-of-function allele of a gene encoding a protein containing stomatin-like and lipid transfer domains that indirectly regulates Na^+^ channels, and hence, similar to *slo-1*, *unc-24* affects [Ca^2+^]_i_ through plasma membrane hyperpolarisation [106]–[109].

### Molecular biology and gene cloning

Primer sequences are available in Protocol S2. Protocol S3 provides details about gene cloning and reporter construction.

RNAi was performed as described by cloning PCR amplified products into the L4440 RNAi feeding vector [110]. RNAi constructs for *asp-1* to *asp-6* were obtained from a library [111].

### qPCR

Animals were harvested and RNA was extracted as described by Hope (1999). cDNA was amplified from 1.5 µg of DNAse treated mRNA using Taqman Reverse Transcription Reagents (Applied Biosystems), as directed by the manufacturer. qPCR was performed in triplicate in 20 µl reactions using Fast SYBR Green Master Mix (Applied Biosystems) and analysed using Fast System SDS software (Applied Biosystems); PCR products were verified by agarose gel electrophoresis. Gene expression data were analysed using the ΔΔCT method and normalized using *ama-1* as an endogenous reference gene relative to N2 wildtype animals [112]. Primers were designed to span exon-exon boundaries and when applicable, mRNA was amplified upstream of mutant deletion/insertion sites. The primers used are listed as follows: *ama-1* (PK1084/PK1085); *clp-1* (PK1062/PK1063); *clp-2* (PK1113/PK1114); *clp-3* (PK1066/PK1067); *clp-4* (clp-4_f2/clp-4_r2); *clp-6* (clp-6_f2/clp-6_r2); *clp-7* (PK1074/PK1087); *clp-8* (clp-8_f2/clp-8_r2); *clp-9* (clp-9_f2/clp-9_r2); *clp-10* (clp-10_f2/clp-10_r2); *clpr-1* (clpr1_f2/clpr1_r2); *asp-1* (PK1095/PK1096); *asp-2* (PK1097/PK1098); *asp-3* (PK1099/PK1100); *asp-4* (PK1101/PK1102); *asp-5* (PK1103/PK1104); *asp-6* (PK1105/PK1106).

### Generation of transgenic animals

Worms were transformed by germ line microinjection [113]. Microinjection solutions were composed of the plasmid of interest at 10 µg/ml and a co-transformation marker, either 80 µg/ml of pRF4 *rol-6* (*su1006*) or 50 µg/ml of TG96 *sur-5p*::*gfp*. At least three independent transgenic strains were generated and examined for each construct, but only those displayed in this manuscript are listed, as they are representative of the patterns observed for a given construct. The following extrachromosomal arrays were generated with the pRF4 co-transformation marker: *crEx65* (*clp-1p::nls::mrfp*), *crEx70* (*clp-2p::nls::mrfp)*, *crEx72* (*clp-3p::nls::mrfp*), *crEx74* (*clp-4::nls::mrfp*), *crEx78* (*tra-3p::nls::mrfp*), *crEx83* (*clp-6p::nls::mrfp*), *crEx79* (*clp-7::nls::mrfp*), *crEx202* (*clp-1p::gfp*), *crEx110* (*hsp-16.64p::clp-1*), *crEx86* (*hsp-16.64p::clp-2*), *crEx88* (*hsp-16.64p::clp-4*), *crEx8* (*hsp-16.64p::tra-3*), *crEx96* (*hsp-16.64p::clp-7*) and *crEx333* (*unc-54p::clp-1::mrfp*). The following extrachromosomal arrays were generated with the TG96 co-transformation marker: *crEx325* (*unc-54p::clp-1*), *crEx141* (*unc-54p::clp-2*), *crEx147* (*unc-54p::clp-4*), *crEx263* (*unc-54p::tra-3*), *crEx258* (*unc-54p::clp-7*), *crEx241* (*unc-47p::clp-1*), *crEx190* (*unc-119p::clp-1*), *crEx319* (*unc-54p::clp-1(C371A)*), *crEx335* (*unc-54p::clp-1::mrfp*) and *crEx336* (*unc-54p::clp-1(C371A)::mrfp*).

The *unc-54p::clp-1*(*crEx325*) extrachromosomal array, *crEx325* was chromosomally integrated by gamma irradiation using a ^137^Cs source (RX30/50, Gravatom Industries). The integrated strain, *crIs4* (*unc-54p::clp-1*) was outcrossed five times with N2 prior to study.

### Phenotypic analyses

Tissue-specific GFP reporters used to identify tissues expressing calpain reporters include: *unc-119::gfp*, a pan-neuronal marker [114]; *unc-47::gfp*, which is specifically expressed in GABAergic neurons of the ventral nerve cord [115]; *myo-3::gfp::nls*, which is expressed in all muscle cells except those of the pharynx [116]; *scm::gfp*, a seam cell marker [117]; and *pes-6::gfp*, a excretory cell GFP reporter.

Brood size and embryonic lethality was scored by placing individual L4 staged hermaphrodites on NGM plates and transferring them daily to a fresh plate until egg laying had ceased. The number of dead eggs (embryonic lethality) and live animals (brood size) were scored two days after transfer of the mother.

The percentage of animals displaying paralysis was scored on day 2 of adulthood after first obtaining synchronized populations of animals of the specified genotype by bleaching gravid adults with alkaline hypochlorite [118]. Phenotypic scoring was performed by gently prodding animals with a platinum pick and registering their response: Unc animals retained the ability to move, but with impaired mobility; paralyzed animals failed to migrate.

### Phalloidin staining

Animals were stained with Alexa Fluor 594 phalloidin (Invitrogen). Briefly, day 2 adults displaying wildtype, Unc or paralyzed phenotypes were lyophilized in an Automatic Environmental Speedvac (Savant) prior to fixation in ice-cold acetone. Animals were resuspended in 20 µl S-Mix (0.2 M Na phosphate (pH 7.5), 1 mM MgCl_2_, 0.004% (w/v) SDS) containing 2 U Alexa Fluor 594 phalloidin, incubated for 1 hour in the dark and washed twice with PBS-Tween 20 (0.5%) (Sigma) before viewing.

### Western blotting

Eighty day 2 adult animals were placed in 20 µl of SDS protein sample buffer, electrophoresed on 10% or 4–12% gradient SDS-polyacrylamide gels and transferred to nitrocellulose membranes. Blots were incubated with primary antibodies at the following concentrations: anti-mRFP/GFP primary antibody (Invitrogen), 1∶1000; anti-myc 9E10, 1∶1000; anti-α tubulin (Abcam), 1∶4000; anti-actin (Sigma), 1∶1000. Protein was visualized using anti-mouse or anti-rabbit horseradish peroxidase (HRP) linked antibodies (Amersham) at 1∶5000 dilution and Western Lightning™ chemiluminescent substrate (Perkin Elmer). Nitrocellulose membranes were stripped using Restore Plus stripping buffer (Thermo Scientific). Protein levels were quantified using ChemiDoc-It imaging system (UVP).

### Nemadipine-A treatment

Worms were grown in wells of 24-well plates containing 1 ml of MYOB agar [119], including 5 µM nemadipine-A (kindly provided by Peter Roy) or 0.01% DMSO (control), as described [72]. Plates were incubated at 20°C, except those carrying *hlh-1*(*cc561ts*) mutants, which were raised at 15°C. Phenotypic scoring is described above.

### Microscopy

Animals were immobilized using 10 mM sodium azide. Differential interference contrast (DIC) and fluorescent images were captured with a Zeiss Axioskop 2 fitted with an ORCA-ER (Hamamatsu) digital camera driven by Openlab 4 software (Improvision), or a Zeiss LSM 710 confocal driven by Zeiss Zen software.

### Statistical analysis

Statistical analysis was performed using Student's T-test.

## Supporting Information

Figure S1 The modular arrangement of Ca^2+^-activated calpain proteases. Atypical and typical calpains share a conserved catalytic DII, which is further separated into Ca^2+^ binding sites, IIa and IIb. Residues of the catalytic triad are highlighted with arrows; missing catalytic residues are marked with an X. Many calpains have a short non-conserved DI sequence, which are subject to autolysis, and a DIII that carries a C2 Ca^2+^-binding domain. DIV is exclusive to typical calpains and is distinguished by the presence of a penta EF-hand domain; the fifth EF-hand motif mediates heterodimerization with a small regulatory subunit CAPNS1 (not shown). A number of additional domains and motifs are also associated with calpain proteins: G and G E, regions rich in glycine or glycine and glutamate, respectively; SEEL, a potential C-terminal ER target sequence; PBH, PalB homology domain with some domain III homology; T, C2 domain originally identified in TRA-3 [33]; Zn^2+^, zinc finger motif-containing; SolH, small optic lobes (SOL) homology domain; and MIT 1, microtubule interacting and transport domain.
(TIF)

Figure S2 Sequence alignments of calpain proteins. Protein sequence alignment of fourteen *C. elegans* calpains (CLP-1, CLP-2, CLP-3, CLP-4, TRA-3/CLP-5, CLP-6, CLP-7, CLP-8, CLP-9, CLP-10, CLPR-1, CLPR-2, CLPR-3, CLPR-4) and human calpains (CAPN1 and CAPN2). Domains are highlighted with coloured lines as follows: DI (green), DII (red) and DIII (purple). The catalytic residues C, H and N are highlighted with red boxes. Conserved residues involved in coordinating Ca^2+^ within DII are highlighted yellow, and residues R and E which coordinate the Ca^2+^ induced conformational change between domain IIa and IIb in human CAPN2 are highlighted yellow and indicated with arrows [8]. Conserved residues from the RanBP2 zinc finger type signature for CLP-9 are highlighted in light blue. Proteins were aligned using ClustalW version 1.83 and shaded using GeneDoc version 2.6.0.2. Black boxes highlight greater than 95% similarity, dark grey boxes greater than 80% similarity, and light grey boxes greater than 60% similarity.
(DOC)

Figure S3 Phylogenetic relationships between calpain proteins. Cladogram of calpain and calpain-like proteins from worm, fly and human, which is rooted using the prokaryote calpain-related protein, TPR-1, from *Porphyromonas gingivalis* (Pg). Prefixes used to identify species include: *C. elegans* (Cel), *C. briggsae* (Cbr), *D. melanogaster* (Dm) and *H. sapiens* (Hs). GenBank accession numbers are provided in the Methods section.
(TIF)

Figure S4 Identification of proteolytically inactive calpain-like proteins. (A) Inactive calpain proteins (344) that are missing key residues of the catalytic triad were identified across all eukaryotic kingdoms. (B) Inactive calpain proteins (100) were found distributed across animal phyla, ranging from ancient Placozoa and Cnidaria to Chordata.
(TIF)

Figure S5 Calpain deletion mutants do not display defects in body wall muscle. Representative images of phalloidin stained body wall muscle from (A) *clp-1* (*tm690*), (B) *clp-4 (ok2808)*, (C) *clp-6 (ok1779)*, and (D) *clp-7 (ok2750)* deletion mutants at day 3 of adulthood. Following phalloidin staining, the 20 most central muscle cells from each of the two most visible body wall muscle quadrants were scored (40 cells per animal), as described in Gieseler et al. (2000). Abnormal body wall muscle cells were not seen in any of the four *clp* mutants from 3 independent experiments involving at least 30 animals per experiment. Scale bar is 20 µm.
(TIF)

Figure S6 Expression patterns of five *C. elegans* atypical calpain transcriptional reporters. (A) Basic construction of *nls::mrfp* expression reporters driven from calpain promoters. (B–F) Nuclear localized expression patterns of calpain reporters in adult hermaphrodites. (B) *clp-1p::nls::mrfp* (*crEx65*) (C) *clp-2p::nls::mrfp* (*crEx70*) (D) *clp-4p::nls::mrfp* (*crEx74*) (E) *tra-3p::nls::mrfp* (*crEx78*) (F) *clp-7p::nls::mrfp* (*crEx79*). Each micrograph is typical of the pattern observed with at least two other independent transgenic strains generated with the same reporter construct. Nomarski DIC micrograph, left; mRFP fluorescence micrograph, right. A montage of overlapping images captured in the same focal plane was created to show the entire worm. Scale bar, 50 µM.
(TIF)

Figure S7 Larval stage expression patterns of five *C. elegans* atypical calpain transcriptional reporters. (A–E) Nuclear localized expression patterns of calpain reporters at different larval stages. (A) L2 larvae expressing *clp-1p::nls::mrfp* (*crEx65*), (B) L3 larvae expressing *clp-2p::nls::mrfp* (*crEx70*), (C) L4 larvae expressing *clp-4p::nls::mrfp* (*crEx74*), (D) L2 larvae expressing *tra-3p::nls::mrfp* (*crEx78*), (E) L1 larvae expressing *clp-7p::nls::mrfp* (*crEx79*). Nomarski DIC micrograph, left; mRFP fluorescent micrograph, right. Scale bar, 10 µM.
(TIF)

Figure S8 Co-localization of calpain *nls::mrfp* transcriptional reporters and the GABA-ergic *unc-47::gfp* reporter. (A) *clp-1p::nls::mrfp* (*crEx65*) (B) *clp-4p::nls::mrfp* (*crEx74*), and (C) *clp-7p::nls::mrfp* (*crEx79*) co-localize with the *unc-47::gfp* reporter. *unc-47::gfp* reporter, left (green); calpain promoter driven *nls::mrfp* expression, middle (red); and co-localization, right (yellow). The *tra-3/clp-5::mrfp* reporter fails to co-localize with *unc-47::gfp* (data not shown). Each micrograph is typical of the pattern observed with at least two other independent transgenic strains generated with the same reporter construct. Scale bar, 10 µM.
(TIF)

Figure S9 Atypical calpain expression is associated with the intestine, vulva and hypodermis. Intestinal expression, indicated with arrows, is detected in (A) *clp-2p::nls::mrfp* (*crEx70*), (B) *tra-3p::nls::mrfp* (*crEx78*) and (C) *clp-7p::nls::mrfp* (*crEx79*). *tra-3p::nls::mrfp* (*crEx78*) is expressed in (D) the vulva and (E) the hypodermis. *clp-7p::nls::mrfp* (*crEx79*) is expressed in (F) the vulva and (G) the hypodermis. Vulval and hypodermal expression are indicated with a (v) and arrowheads, respectively. Nomarski DIC micrographs were overlaid with false colored mRFP fluorescence micrographs, and the entire image was converted to greyscale. Each micrograph is typical of the pattern observed with at least two other independent transgenic strains generated with the same reporter construct. Scale bar, 10 µm.
(TIF)

Figure S10 Test to discriminate between phenotypically Unc and paralyzed adult animals expressing *unc-54p::clp-1*. Animals were mechanically prodded (arrowhead) at t = 0 and examined for their ability to move away from a stimulus over a 10 s interval. Unc animals (top panel) are able to change body position, but have impaired mobility. Paralyzed animals (middle panel) show marginal movement of head and/or tail and an absence of mobility. The behavior of a wildtype adult (bottom panel) is shown by comparison. The time elapsed after the application of mechanical stimulus is indicated. Scale bars, 500 µm.
(TIF)

Figure S11 Detection and comparison of transgenic CLP-1::mRFP protein levels. (A) CLP-1::myc levels in N2 wildtype, *unc-54p::clp-1::myc* (*crEx325*) and *hsp16-41p::clp-1::myc* (*crEx329*) animals without (−HS) and four hours after heat shock (+HS). α-tubulin (TBA) provides a protein loading reference and CLP-1::myc expression levels are presented as a percentage of *unc-54p::clp-1::myc* protein levels and normalized to TBA. (B) CLP-1::myc expression levels in N2 wildtype and in *hsp16-41p::clp-1::myc* (*crEx329*) animals taken at intervals following heat shock. TBA provides a protein loading reference. CLP-1::myc expression levels are presented as a percentage of *hsp16-41p::clp-1::myc* protein levels 8 hours post heat shock and normalized to TBA. (C) CLP-1::mRFP protein expression levels in wildtype *unc-54p::clp-1*::*mrfp* (*crEx335*) and catalytically inactive *unc-54p::clp-1(C371A)::mrfp* (*crEx336*) animals. Actin was used as a protein loading reference. CLP-1::mRFP expression levels are presented as a percentage of *unc-54p::clp-1*::mRFP protein levels and normalized to actin. Values represent the average from two independent experiments.
(TIF)

Figure S12 Translational expression pattern of *clp-1::gfp*. (A) *clp-1::gfp* (*crEx202*) is expressed throughout the animal. Scale bar, 50 µM. (B–F) *clp-1::gfp* (*crEx202*) is expressed in many somatic tissues, including: (B) the head – pharyngeal muscles and the nerve ring, (C) body wall muscle, (D) the ventral nerve cord and muscle arms (white arrows), (E) vulva, (F) intestine. Each micrograph is typical of the pattern observed with at least two other independent transgenic *clp-1::gfp* strains. Scale bar, 10 µM.
(TIF)

Protocol S1 Protein blast and phylogenetic analyses.
(DOC)

Protocol S2 Oligonucleotide sequences.
(DOC)

Protocol S3 Molecular biology and gene cloning methods.
(DOC)

Table S1 Eukaryotic catalytically inactive calpains.
(DOC)

Table S2 Effects of calpain mutants and feeding RNAi on brood size and embryonic lethality.
(DOC)

Table S3 Effects of ectopic *clp-1* expression on brood size and embryonic lethality.
(DOC)

Table S4 qPCR analysis of *asp-1* to *asp-6* (*RNAi*).
(DOC)

Text S1 Profile hidden Markov models detecting EF hand motifs.
(TXT)
