# Supplementary figures and images for: The Atypical Calpains: Evolutionary Analyses and Roles in Caenorhabditis elegans Cellular Degeneration
Source: PLoS Genet. 2012 Mar 29;8(3):e1002602. doi: 10.1371/journal.pgen.1002602 (PMC3315469; doi:10.1371/journal.pgen.1002602)

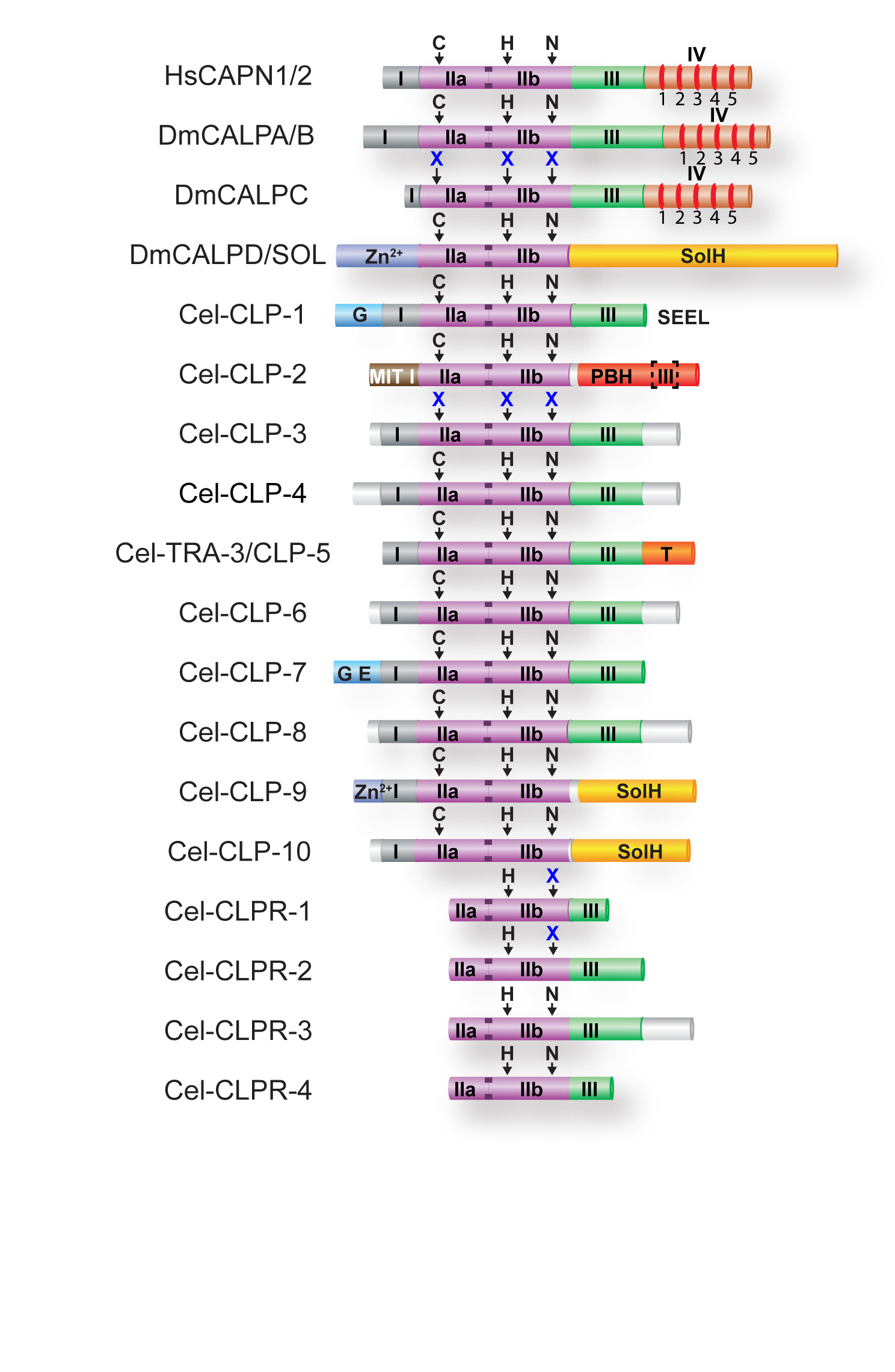

Supplement: Figure S1 — The modular arrangement of Ca2+-activated calpain proteases. Atypical and typical calpains share a conserved catalytic DII, which is further separated into Ca2+ binding sites, IIa and IIb. Residues of the catalytic triad are highlighted with arrows; missing catalytic residues are marked with an X. Many calpains have a short non-conserved DI sequence, which are subject to autolysis, and a DIII that carries a C2 Ca2+-binding domain. DIV is exclusive to typical calpains and is distinguished by the presence of a penta EF-hand domain; the fifth EF-hand motif mediates heterodimerization with a small regulatory subunit CAPNS1 (not shown). A number of additional domains and motifs are also associated with calpain proteins: G and G E, regions rich in glycine or glycine and glutamate, respectively; SEEL, a potential C-terminal ER target sequence; PBH, PalB homology domain with some domain III homology; T, C2 domain originally identified in TRA-3 [33]; Zn2+, zinc finger motif-containing; SolH, small optic lobes (SOL) homology domain; and MIT 1, microtubule interacting and transport domain. (TIF) [file pgen.1002602.s001.tif]

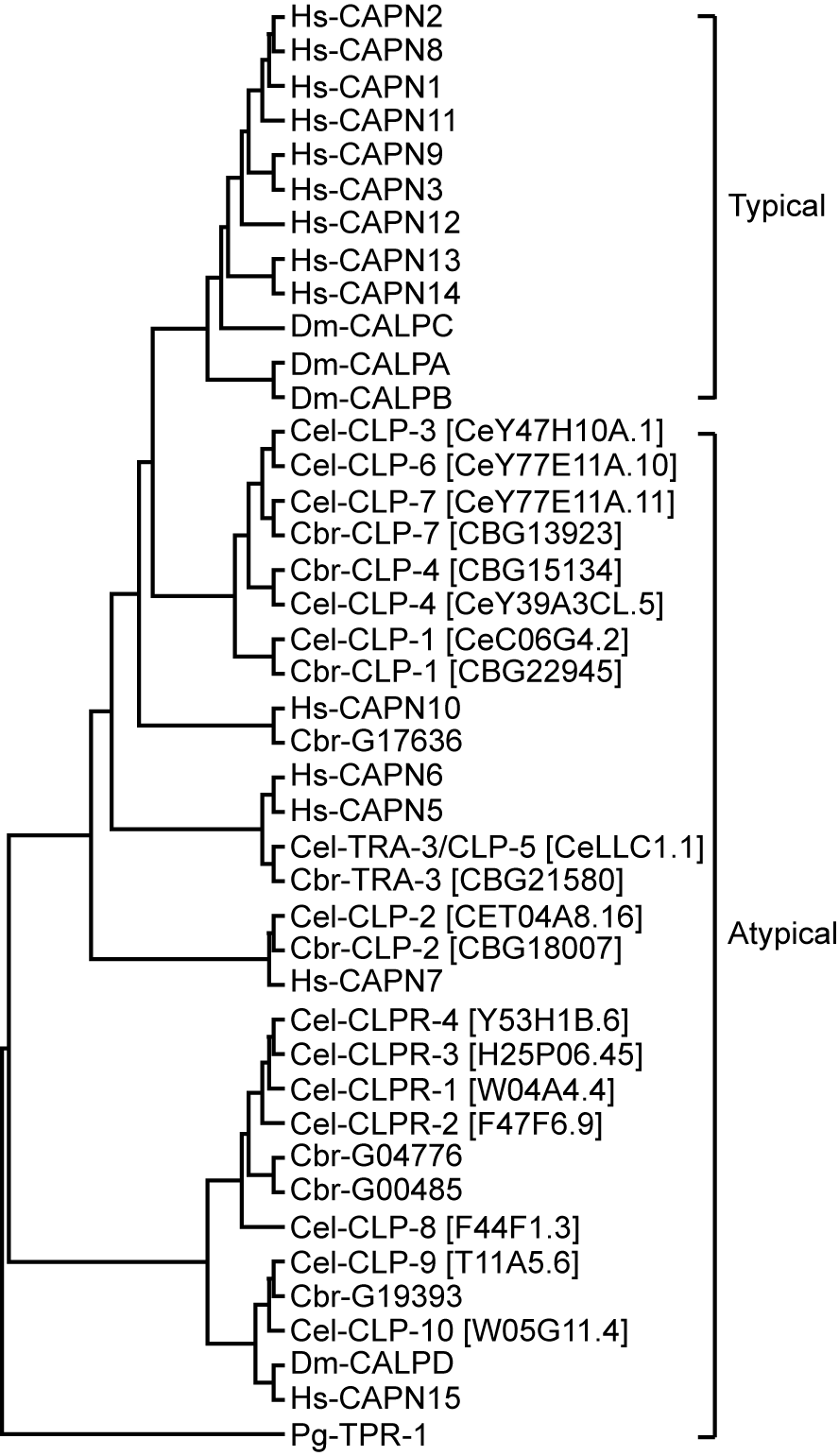

Supplement: Figure S3 — Phylogenetic relationships between calpain proteins. Cladogram of calpain and calpain-like proteins from worm, fly and human, which is rooted using the prokaryote calpain-related protein, TPR-1, from Porphyromonas gingivalis (Pg). Prefixes used to identify species include: C. elegans (Cel), C. briggsae (Cbr), D. melanogaster (Dm) and H. sapiens (Hs). GenBank accession numbers are provided in the Methods section. (TIF) [file pgen.1002602.s003.tif]

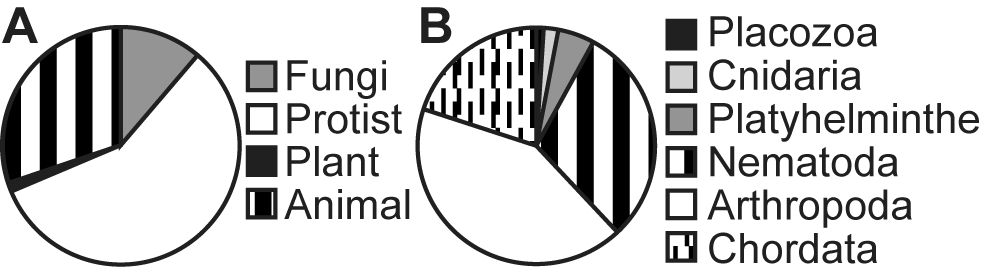

Supplement: Figure S4 — Identification of proteolytically inactive calpain-like proteins. (A) Inactive calpain proteins (344) that are missing key residues of the catalytic triad were identified across all eukaryotic kingdoms. (B) Inactive calpain proteins (100) were found distributed across animal phyla, ranging from ancient Placozoa and Cnidaria to Chordata. (TIF) [file pgen.1002602.s004.tif]

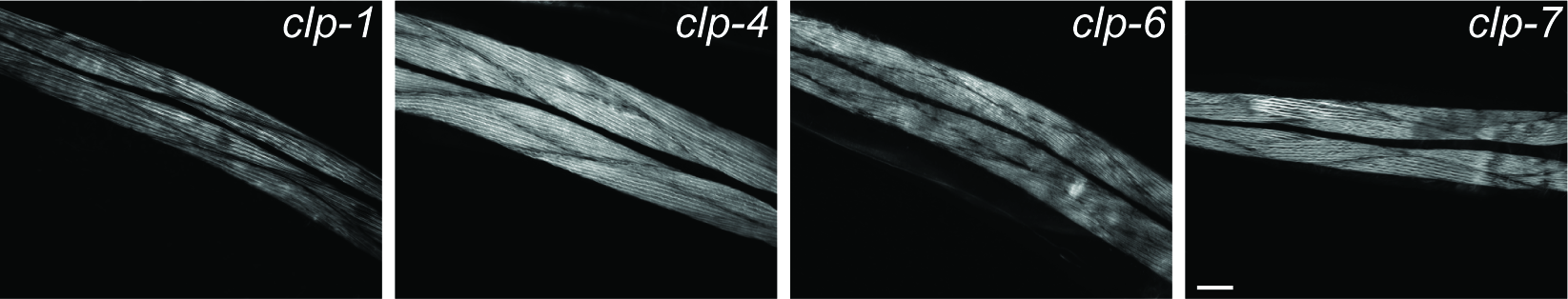

Supplement: Figure S5 — Calpain deletion mutants do not display defects in body wall muscle. Representative images of phalloidin stained body wall muscle from (A) clp-1 (tm690), (B) clp-4 (ok2808), (C) clp-6 (ok1779), and (D) clp-7 (ok2750) deletion mutants at day 3 of adulthood. Following phalloidin staining, the 20 most central muscle cells from each of the two most visible body wall muscle quadrants were scored (40 cells per animal), as described in Gieseler et al. (2000). Abnormal body wall muscle cells were not seen in any of the four clp mutants from 3 independent experiments involving at least 30 animals per experiment. Scale bar is 20 µm. (TIF) [file pgen.1002602.s005.tif]

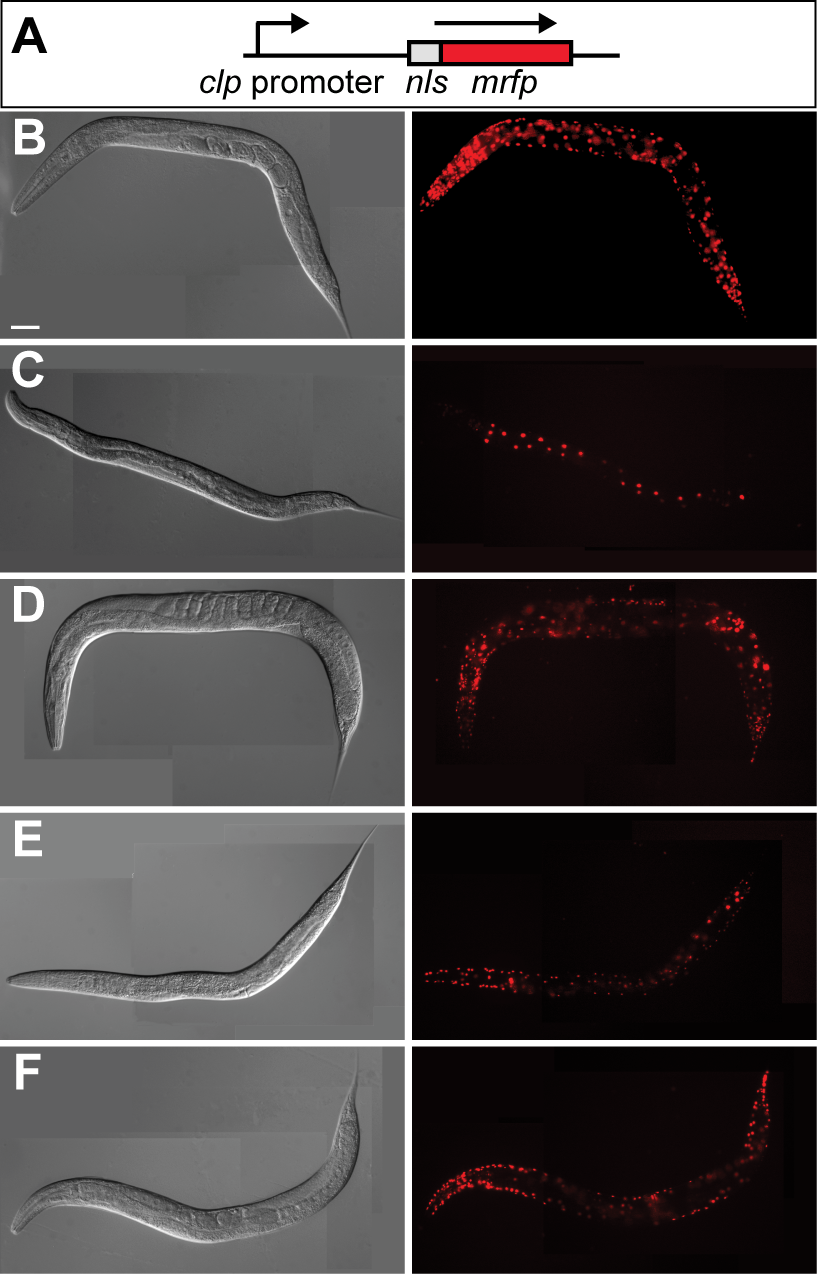

Supplement: Figure S6 — Expression patterns of five C. elegans atypical calpain transcriptional reporters. (A) Basic construction of nls::mrfp expression reporters driven from calpain promoters. (B–F) Nuclear localized expression patterns of calpain reporters in adult hermaphrodites. (B) clp-1p::nls::mrfp (crEx65) (C) clp-2p::nls::mrfp (crEx70) (D) clp-4p::nls::mrfp (crEx74) (E) tra-3p::nls::mrfp (crEx78) (F) clp-7p::nls::mrfp (crEx79). Each micrograph is typical of the pattern observed with at least two other independent transgenic strains generated with the same reporter construct. Nomarski DIC micrograph, left; mRFP fluorescence micrograph, right. A montage of overlapping images captured in the same focal plane was created to show the entire worm. Scale bar, 50 µM. (TIF) [file pgen.1002602.s006.tif]

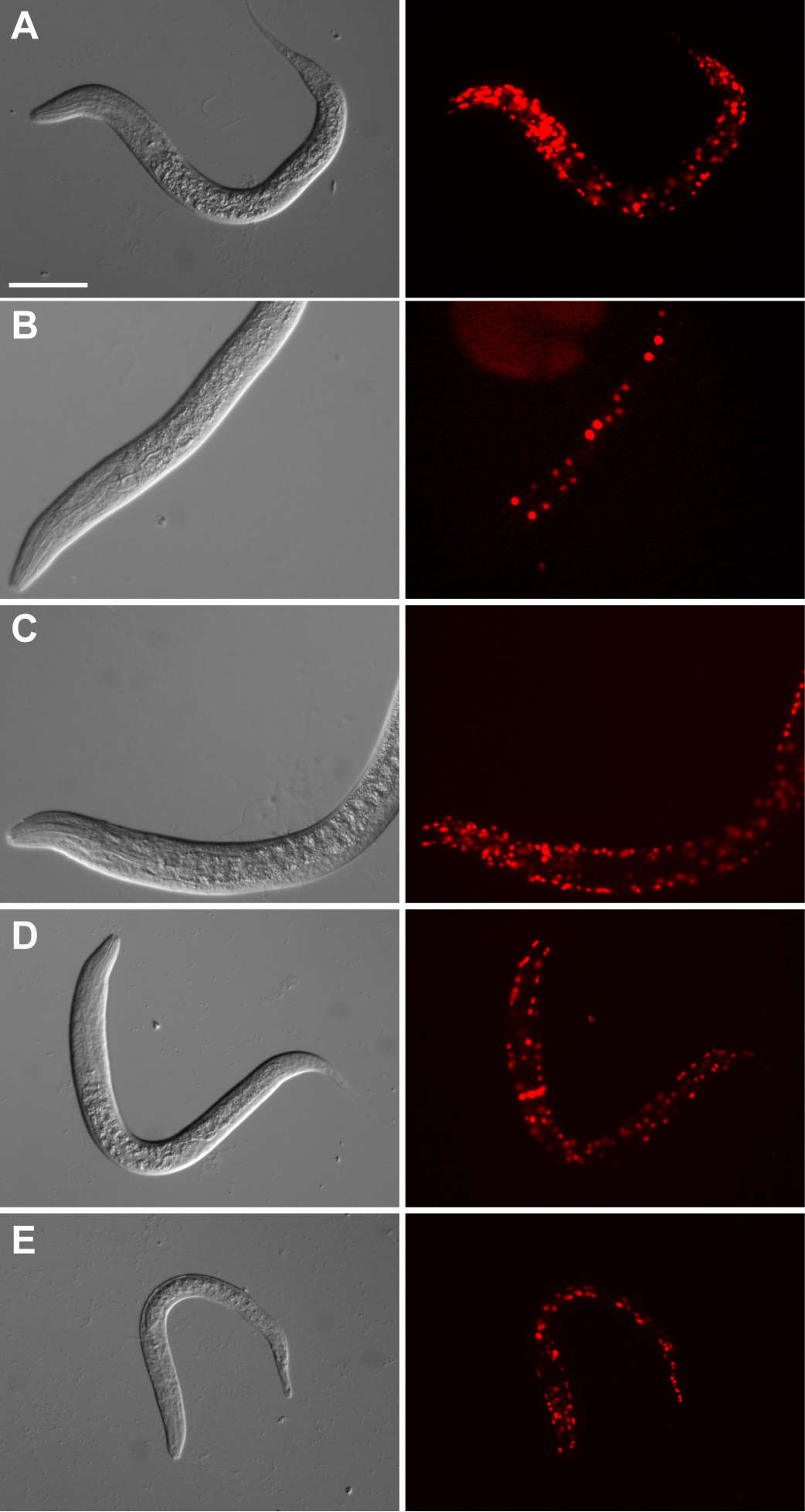

Supplement: Figure S7 — Larval stage expression patterns of five C. elegans atypical calpain transcriptional reporters. (A–E) Nuclear localized expression patterns of calpain reporters at different larval stages. (A) L2 larvae expressing clp-1p::nls::mrfp (crEx65), (B) L3 larvae expressing clp-2p::nls::mrfp (crEx70), (C) L4 larvae expressing clp-4p::nls::mrfp (crEx74), (D) L2 larvae expressing tra-3p::nls::mrfp (crEx78), (E) L1 larvae expressing clp-7p::nls::mrfp (crEx79). Nomarski DIC micrograph, left; mRFP fluorescent micrograph, right. Scale bar, 10 µM. (TIF) [file pgen.1002602.s007.tif]

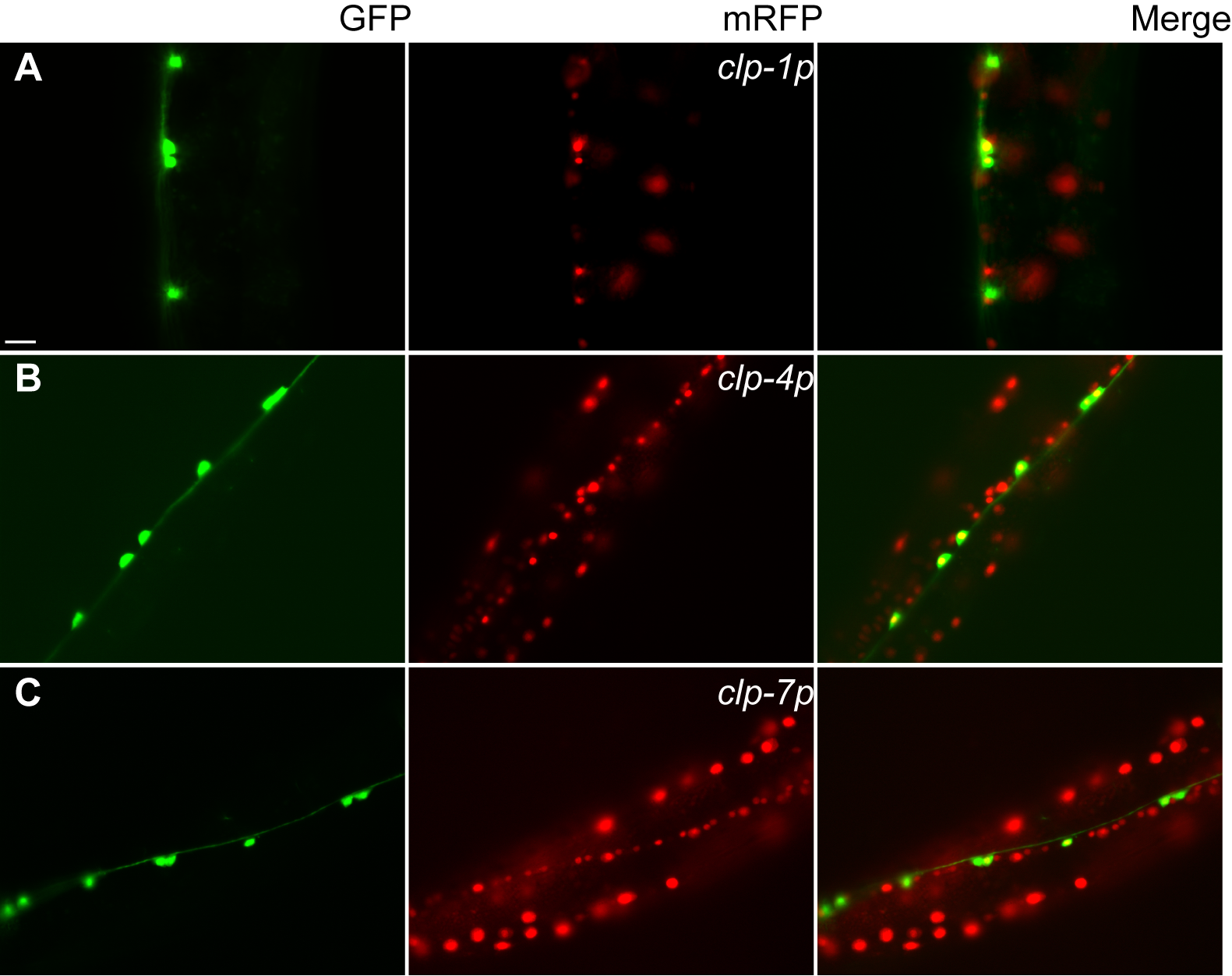

Supplement: Figure S8 — Co-localization of calpain nls::mrfp transcriptional reporters and the GABA-ergic unc-47::gfp reporter. (A) clp-1p::nls::mrfp (crEx65) (B) clp-4p::nls::mrfp (crEx74), and (C) clp-7p::nls::mrfp (crEx79) co-localize with the unc-47::gfp reporter. unc-47::gfp reporter, left (green); calpain promoter driven nls::mrfp expression, middle (red); and co-localization, right (yellow). The tra-3/clp-5::mrfp reporter fails to co-localize with unc-47::gfp (data not shown). Each micrograph is typical of the pattern observed with at least two other independent transgenic strains generated with the same reporter construct. Scale bar, 10 µM. (TIF) [file pgen.1002602.s008.tif]

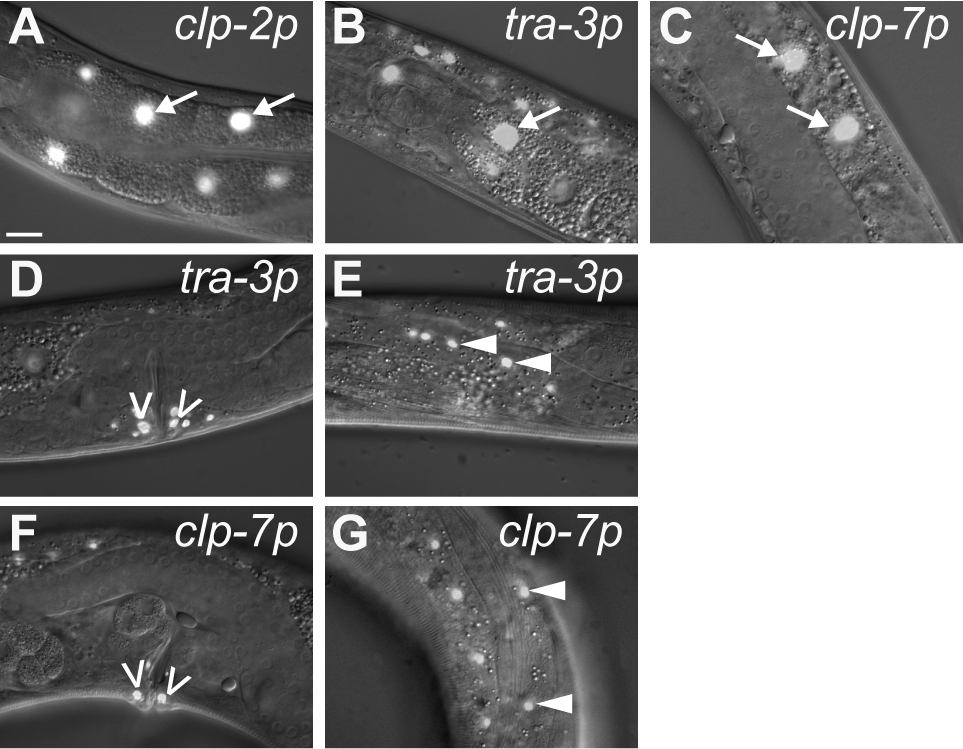

Supplement: Figure S9 — Atypical calpain expression is associated with the intestine, vulva and hypodermis. Intestinal expression, indicated with arrows, is detected in (A) clp-2p::nls::mrfp (crEx70), (B) tra-3p::nls::mrfp (crEx78) and (C) clp-7p::nls::mrfp (crEx79). tra-3p::nls::mrfp (crEx78) is expressed in (D) the vulva and (E) the hypodermis. clp-7p::nls::mrfp (crEx79) is expressed in (F) the vulva and (G) the hypodermis. Vulval and hypodermal expression are indicated with a (v) and arrowheads, respectively. Nomarski DIC micrographs were overlaid with false colored mRFP fluorescence micrographs, and the entire image was converted to greyscale. Each micrograph is typical of the pattern observed with at least two other independent transgenic strains generated with the same reporter construct. Scale bar, 10 µm. (TIF) [file pgen.1002602.s009.tif]

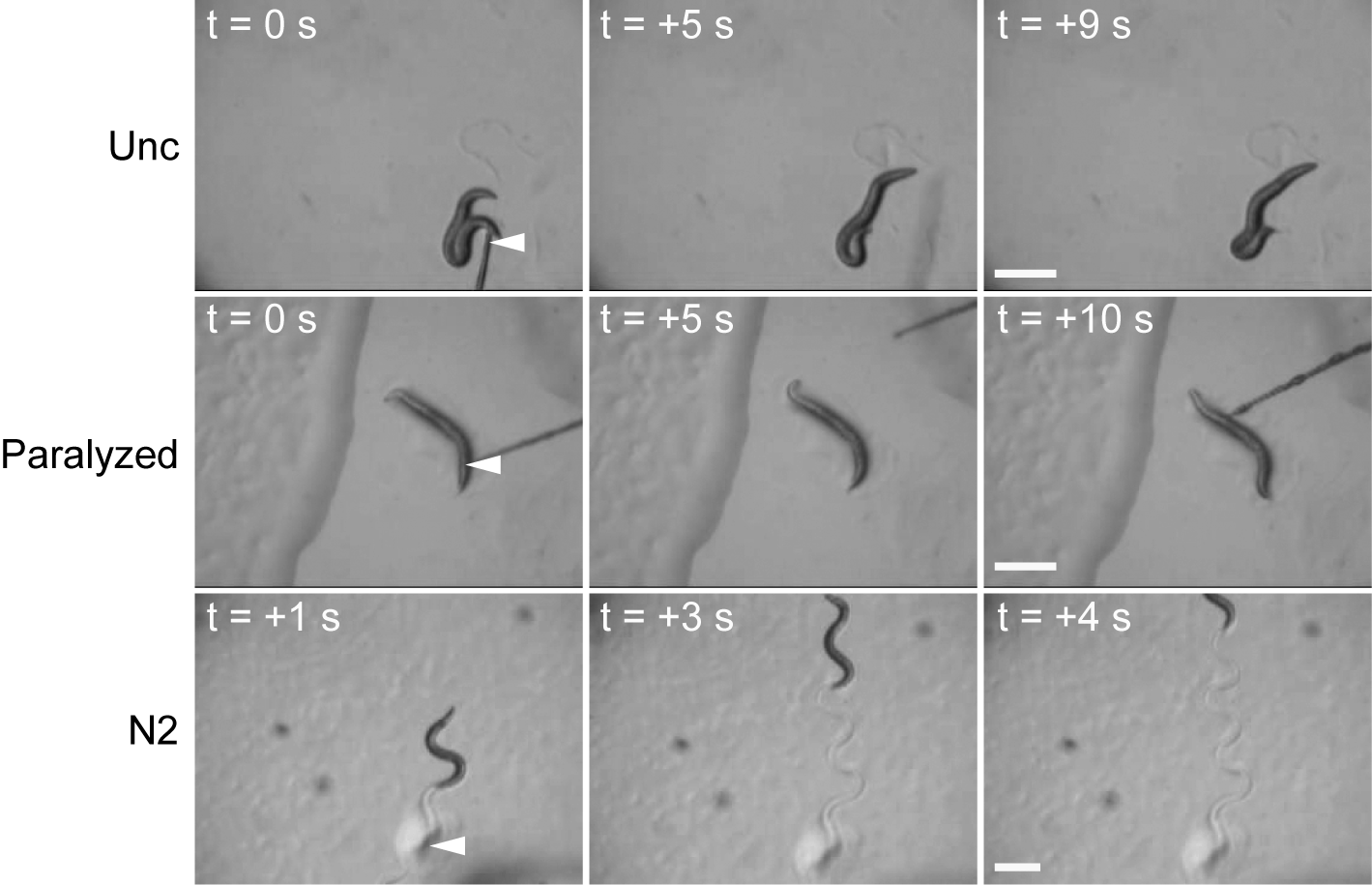

Supplement: Figure S10 — Test to discriminate between phenotypically Unc and paralyzed adult animals expressing unc-54p::clp-1. Animals were mechanically prodded (arrowhead) at t = 0 and examined for their ability to move away from a stimulus over a 10 s interval. Unc animals (top panel) are able to change body position, but have impaired mobility. Paralyzed animals (middle panel) show marginal movement of head and/or tail and an absence of mobility. The behavior of a wildtype adult (bottom panel) is shown by comparison. The time elapsed after the application of mechanical stimulus is indicated. Scale bars, 500 µm. (TIF) [file pgen.1002602.s010.tif]

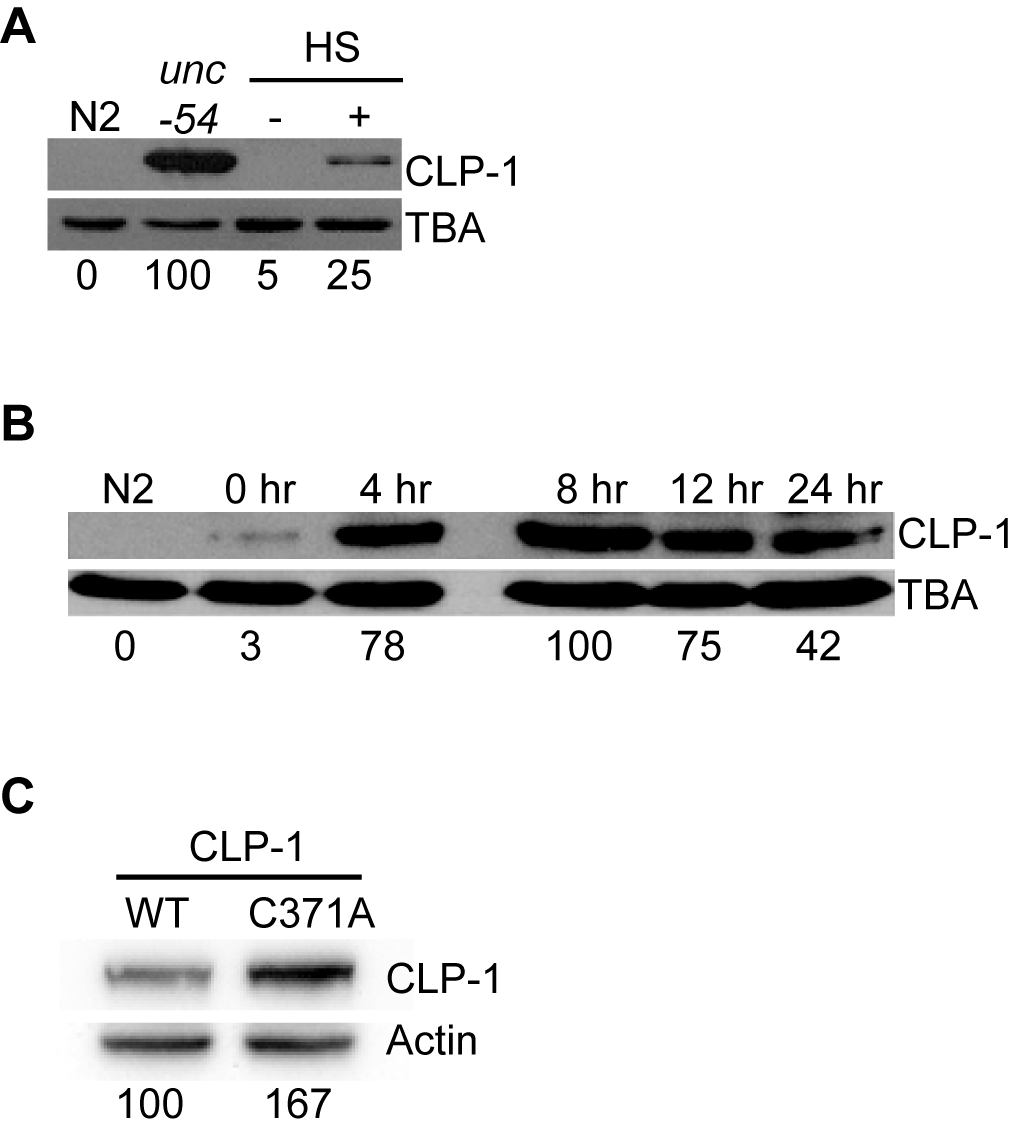

Supplement: Figure S11 — Detection and comparison of transgenic CLP-1::mRFP protein levels. (A) CLP-1::myc levels in N2 wildtype, unc-54p::clp-1::myc (crEx325) and hsp16-41p::clp-1::myc (crEx329) animals without (−HS) and four hours after heat shock (+HS). α-tubulin (TBA) provides a protein loading reference and CLP-1::myc expression levels are presented as a percentage of unc-54p::clp-1::myc protein levels and normalized to TBA. (B) CLP-1::myc expression levels in N2 wildtype and in hsp16-41p::clp-1::myc (crEx329) animals taken at intervals following heat shock. TBA provides a protein loading reference. CLP-1::myc expression levels are presented as a percentage of hsp16-41p::clp-1::myc protein levels 8 hours post heat shock and normalized to TBA. (C) CLP-1::mRFP protein expression levels in wildtype unc-54p::clp-1::mrfp (crEx335) and catalytically inactive unc-54p::clp-1(C371A)::mrfp (crEx336) animals. Actin was used as a protein loading reference. CLP-1::mRFP expression levels are presented as a percentage of unc-54p::clp-1::mRFP protein levels and normalized to actin. Values represent the average from two independent experiments. (TIF) [file pgen.1002602.s011.tif]

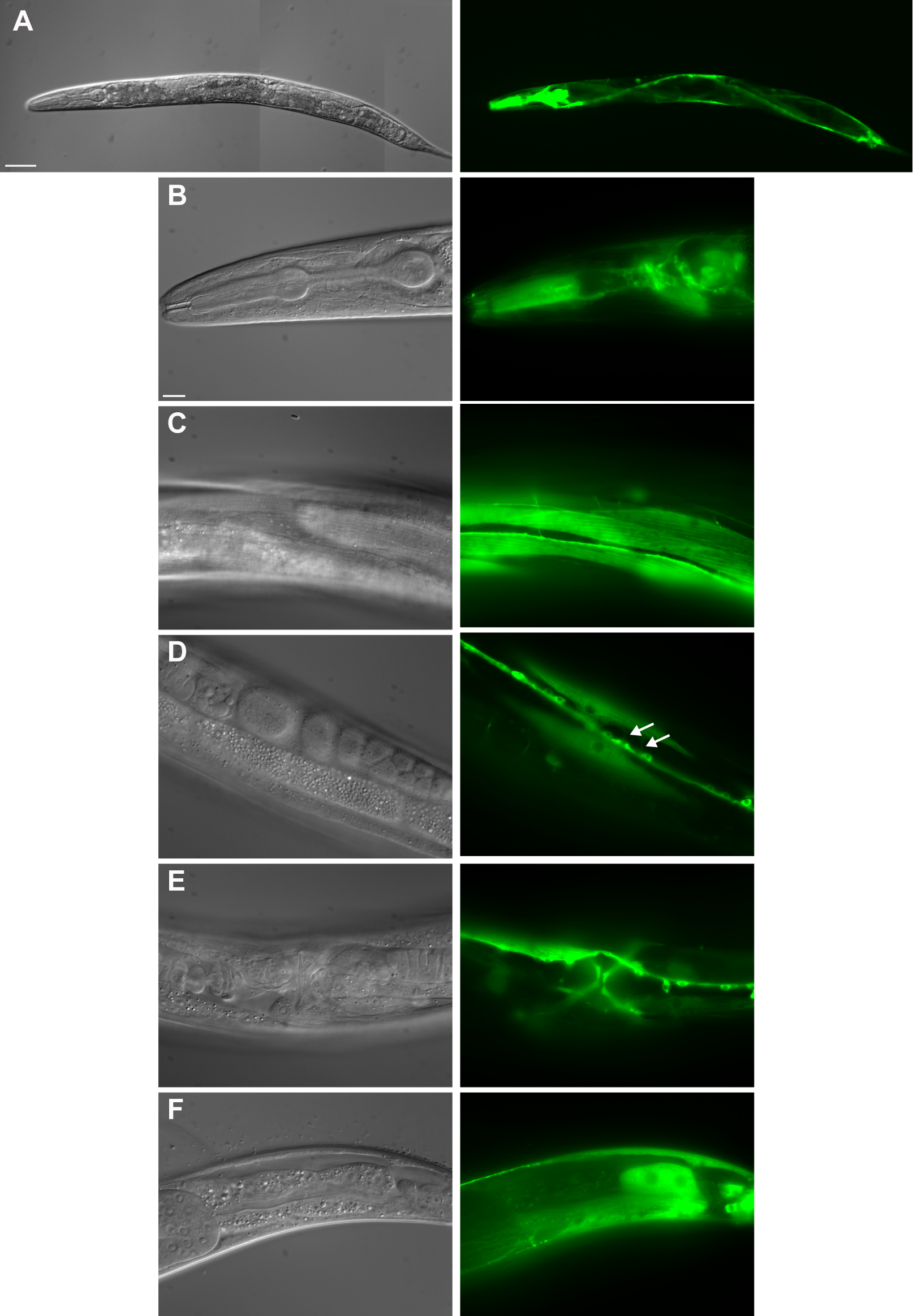

Supplement: Figure S12 — Translational expression pattern of clp-1::gfp. (A) clp-1::gfp (crEx202) is expressed throughout the animal. Scale bar, 50 µM. (B–F) clp-1::gfp (crEx202) is expressed in many somatic tissues, including: (B) the head – pharyngeal muscles and the nerve ring, (C) body wall muscle, (D) the ventral nerve cord and muscle arms (white arrows), (E) vulva, (F) intestine. Each micrograph is typical of the pattern observed with at least two other independent transgenic clp-1::gfp strains. Scale bar, 10 µM. (TIF) [file pgen.1002602.s012.tif]
