# Supplementary material for: The Atypical Calpains: Evolutionary Analyses and Roles in Caenorhabditis elegans Cellular Degeneration
Source: PLoS Genet. 2012 Mar 29;8(3):e1002602. doi: 10.1371/journal.pgen.1002602 (PMC3315469; doi:10.1371/journal.pgen.1002602)
Supplement: Figure S2 — Sequence alignments of calpain proteins. Protein sequence alignment of fourteen C. elegans calpains (CLP-1, CLP-2, CLP-3, CLP-4, TRA-3/CLP-5, CLP-6, CLP-7, CLP-8, CLP-9, CLP-10, CLPR-1, CLPR-2, CLPR-3, CLPR-4) and human calpains (CAPN1 and CAPN2). Domains are highlighted with coloured lines as follows: DI (green), DII (red) and DIII (purple). The catalytic residues C, H and N are highlighted with red boxes. Conserved residues involved in coordinating Ca2+ within DII are highlighted yellow, and residues R and E which coordinate the Ca2+ induced conformational change between domain IIa and IIb in human CAPN2 are highlighted yellow and indicated with arrows [8]. Conserved residues from the RanBP2 zinc finger type signature for CLP-9 are highlighted in light blue. Proteins were aligned using ClustalW version 1.83 and shaded using GeneDoc version 2.6.0.2. Black boxes highlight greater than 95% similarity, dark grey boxes greater than 80% similarity, and light grey boxes greater than 60% similarity. (DOC) [file pgen.1002602.s002.doc]

CAPN1 : ---------------------------------------------------------------------------------------------------- : -
CAPN2 : ---------------------------------------------------------------------------------------------------- : -
CLP-4 : -----------------------------------------------------------------MAN-HAKLVDQEIYRSP------------------ : 16
CLP-7 : -MSDEEEYYEEDGGNDQGGEDYNDDYGG---GGGGGGGGYDDQNQFQEQDYGNEQNYGNQDYGQGYGN-QQDYGNQQDYQQQNYGGGGYDDEYQQQDYQG : 95
CLP-1 : MADDEEEIIQKVEVKPDEFNGLIGSIAGNLIRDKVGGAGGDILGGLASNFFGGGGGGGGGGGGGGFGGGNGGFGGGSNYDQGGNGNSGD----------- : 89
CLP-3 : ---------------------------------------------MATDDYNFPSITTASSEVDSPEEEYEDDKETDAYNFS------------------ : 37
CLP-6 : ---------------------------------------------------------------------------------------------------- : -
TRA-3 : ---------------------------------------------------------------------------------------------------- : -
CLP-8 : ---------------------------------------------------------------------------------------------------- : -
CLP-2 : ---------------------------------------------------------------------------------------------------- : -
CLPR-1 : ---------------------------------------------------------------------------------------------------- : -
CLPR-2 : ---------------------------------------------------------------------------------------------------- : -
CLPR-3 : ---------------------------------------------------------------------------------------------------- : -
CLPR-4 : ---------------------------------------------------------------------------------------------------- : -
CLP-9 : ---------------------------------------------------------------------------------------------------- : -
CLP-10 : ---------------------------------------------------------------------------------------------------- : -

CAPN1 : ---------------------------------------------------------------------------------------------------- : -
CAPN2 : ---------------------------------------------------------------------------------------------------- : -
CLP-4 : ------------------------VDDFELNYQEYLNDDDDD---KQEAPVAVSKAPKGKGSNHGLGCRMK--------GLFDGFGGTDLKTIMRHLRRR : 81
CLP-7 : GYDDSEQYEEQEEAEPEPEEQEDYSNDVEHSEEEEEEDDDDDGYTQAAANTNYGENDGGNNENHGMGSLIK--------GAMDGFGGGGFDGVVGQIGSL : 187
CLP-1 : --------QQKRKRDMAKDLIGGIFDNVVNRKGKKEQDNYGGGGNYGGGGGNQGGGGGGGFNFNDIGGLINSMGGGGGGGQRQGGGGGGFGDILGGIGSL : 181
CLP-3 : ----------------------TITNESSEVARQREEENKDEKEIARTSFSSISEHDEKIHAYN-FSEILK--------EFVGDDCGDVAGKLFDQLSSM : 106
CLP-6 : -----------------------MSDE--------EVLNNED------------EQEENINNNN-LPSIVK--------EFVSDYTGSIWDELLRQLTAL : 48
TRA-3 : ---------------------------------------------------------------------------------------------------- : -
CLP-8 : ---------------------------------------------------------------------------------------------------- : -
CLP-2 : ---------MDEKATQSAFELSQKAVYYDQGKKYEEAIFCYTEAGNQLLRLVQEKKCLPIFRKNVMECINRAEFLKSNIQNLQQQYPPTTDDIAHNVEYL : 91

CLPR-1 : ---------------------------------------------------------------------------------------------------- : -
CLPR-2 : ---------------------------------------------------------------------------------------------------- : -
CLPR-3 : ---------------------------------------------------------------------------------------------------- : -
CLPR-4 : ---------------------------------------------------------------------------------------------------- : -
CLP-9 : -------------------------------------------------------MEMTAWNCLYCTLINDPAIFICQGCGSQKPLTKKPAKLTGSFPKI : 45
CLP-10 : --------------------------------------------------------------------------------------------MHSSFYLV : 8

CAPN1 : -----------------------------MSEEIITPVYCTGVSAQVQKQRARELG-----LGRHENAIKYLG--------------------------- : 39
CAPN2 : ---------------------------------------MAGIAAKLAKDREAAEG-----LGSHERAIKYLN--------------------------- : 29
CLP-4 : RPKSGQ-----ALTNFMLS---GNMEKVVDQHVKDFVHHFGRINPENGRIMGALRGNDFFNFGGLHNNLGNTGKVYLDNLMKGKCGKKRKVHKFKPIVVE : 173
CLP-7 : ISQGGGGDSSGGLTNVFSS---GGMESIVGNLISSATHQFFGINPATGAIIGAIAGNIIFQMGGQNNSLSSIGKVVLDNIISGKF--KRDVQPFTPG--- : 279
CLP-1 : IGGGGGGQYNGGGGNVNPNNLNGGMVNVIGNLIGEAAHRFLGVDPGTGRIIGAVAGNVIMGLGGKDNSLGNIGKVILDNIISGKF--RRDVDPFVRPGPD : 279
CLP-3 : IPRDSVGR---GLTNIFSS---RELESLAGDLISKNSHDFVAVNSETGGINGALAGNEMFNMGGETNSLSSIGKIVLDNIVSGKF--KRECHVYVPLS-- : 196
CLP-6 : IPRDSSGR---GLTNIFSS---GALESLVGNLISNASHEFFGVNSENGEIIGALAGNAMFNLGGENNSLSSIGKVVLDNIVSGKF--KRECHSYVPLK-- : 138
TRA-3 : --------------------------------------------------------------MTRSEKTRHFGN-------------------------- : 12
CLP-8 : -------------------------------MNSPQEEELLELIEKYNEDKYCTNMNWTIFLGDHENEKEAN---------------------------- : 41
CLP-2 : MVKAAVFQNYENQVDEARQVYENVVEQCLGASRNRTLPQETLKRLRSTAENALKCIEDLVALKKKVTEVELLFPEVPVDDLSNLNFNNQPSPSTPSNQPF : 191
CLPR-1 : ---------------------------------------------------------------------------------------------------- : -
CLPR-2 : ---------------------------------------------------------------------------------------------------- : -
CLPR-3 : ---------------------------------------------------------------------------------------------------- : -
CLPR-4 : ---------------------------------------------------------------------------------------------------- : -
CLP-9 : ITDTVRDVSNAISDLVNNRDRSPAHNFVRATHFRTPLPNGPFVDGQSNDPRDTHQLSMCSKITTTLKQEAEKKSDVIYSNIISYC--------------- : 130
CLP-10 : FANLSPEHDRAIAQFKCRKN---AYKFPKNTIFLKSIYS---------NKKDT----MFLQITPSLKQAQDN-SDVIYDEIIYFS--------------- : 76

CAPN1 : ---------------------------------------------------QDYEQLRVRCLQSGTLFRDEAFPPV--------PQSLGYKDLGPNSSKT : 80
CAPN2 : ---------------------------------------------------QDYEALRNECLEAGTLFQDPSFPAI--------PSALGFKELGPYSSKT : 70
CLP-4 : ELDFGAPKPAVPATPKAPVAPVAPVIPAPAVTPQKPKVDEPTYADSVSDFGLDFETEREKCLRNKTLFEDPEFPAT--------AASLYYRTPPRDR--- : 262
CLP-7 : ----GGGGPGIGFQQQFQT--------------------------------INFQQERQRCLDQRCLFEDPQFPAN--------DSSLYYKTRPDEP--- : 332
CLP-1 : PDRGGGGSGPSPISPRPTTEP------------------------------QDFYELRDQCLESKRLFEDPQFLAN--------DSSLFFSKRPPKR--- : 338
CLP-3 : ------------CQP------------------------------------IYFHEERQKCLDEKRLFEDPQFPAN--------NSSIYVKVRPKDH--- : 237
CLP-6 : ------------CQP------------------------------------LYFYEERQKCLDENRLFEDPQFPAN--------NSSIYYKKPPKSH--- : 179
TRA-3 : ---------------------------------------------------QNYEKLRKICIKKKQPFVDTLFPPT--------NQSLFLEQRQSSD--- : 50
CLP-8 : ---------------------------------------------------EKFKSILEDCQRSKSAFIDKEFPHEPKSYMTFDEQSKVYGKYESWKTWL : 90
CLP-2 : KSPTRNFPPSPSNSQ------------------------------------KFTKEELAVLFTTSNINNKLYVPFHNYDKVNEFKGQQGKFTDPEGKISL : 255
CLPR-1 : ---------------------------------------------------------------------------------------------------- : -
CLPR-2 : ---------------------------------------------------------------------------------------------------- : -
CLPR-3 : ---------------------------------------------------------------------------------------------------- : -
CLPR-4 : ---------------------------------------------------------------------------------------------------- : -
CLP-9 : --------------------------------------------------KSSNSPFIDDSFLHNKKSIGSLQFTK--------NGVKR-HEEPDFLNWL : 171
CLP-10 : --------------------------------------------------TLFNSPFIDPCFRHNLKSIGTFPSTT--------NGVQCNNSIIDSLHWL : 118

**C**

CAPN1 : YGIKWKRPTELLSNPQFIVDGA-----TRTDICQGALGDCWLLAAIASLTLNDTLLHRVVPHGQ--------SFQNGYAGIFHFQLWQFGEWVDVVVDDL : 167
CAPN2 : RGIEWKRPTEICADPQFIIGGA-----TRTDICQGALGDCWLLAAIASLTLNEEILARVVPLNQ--------SFQENYAGIFHFQFWQYGEWVEVVVDDR : 157
CLP-4 : --IIWKRPGEIIANPQLITQGE-----SRFDVKQGALGDCWFLAALANITLYDALFYRIVPPNQ--------SFTENYAGIFHFQFWHYGKWVDVVVDDR : 347
CLP-7 : --IIWKRPGEIYENPQLIVGEK-----SRFDVKQGALGDCWLLAAVANLTLRDELFYRVVPPDQ--------SFTENYAGIFHFQFWRYGQWVDVVIDDR : 417
CLP-1 : --VEWLRPGEITREPQLITEGH-----SRFDVIQGELGDCWLLAAAANLTLKDELFYRVVPPDQ--------SFTENYAGIFHFQFWQYGKWVDVVIDDR : 423
CLP-3 : --IVWKRPGELFANPRLIGSDK-----THFDVKLGRLENRWFLSGAANLSLRDELFYRVVPPDQ--------SFTEKYAGIFHFQFWRYGEWVDVVIDDR : 322
CLP-6 : --IVWLRPAKIFANPRLIVNGK-----SRFDVRQGSLGNCWFLAAAATLAQRDELFYRVVPPDQ--------SFTENYAGIFHFQFWRYGEWVDVVIDDR : 264
TRA-3 : --IVWKRPGELHPDPHLFVEGA-----SPNDVTQGILGNCWFVSACSALTHNFKLLAQVIPDADDQ----EWSTKHAYAGIFRFRFWRFGKWVEVVIDDL : 139
CLP-8 : FPKIWRRITPPSTDVSSSLSVYNPLTFTAYEVFQRKVGDCGLVAALSAISTKPEVIMNIFDSLQ-----------LSKYGVYKVKLFVDGQWKTIIIDDY : 179
CLP-2 : TQKQRMKLKCWKRVSELFENPTIIFSIDCHTIKQTVISDCSFISSLSIAALYEKRFKKQLVTSIIFPQDANGKPIYNPAGKYMFKFHLNGAWRKVLIDDY : 355
CLPR-1 : -------------------------------------------------------------------------MSH---GIVQIRLLINGEWKVLKIDFH : 24
CLPR-2 : -------------------------------------------------------------------------MKH---GIAQIRLLINGEWKVIKIDFH : 24
CLPR-3 : ---------------------------------------------------------------------------------------------------- : -
CLPR-4 : ---------------------------------------------------------------------------------------------------- : -
CLP-9 : RPSQMFTKDGRSSPWSVLNNPR-----PSDIEQGTLVGDCWLMSAMALIAERPDVLDDIIPKKQ--------YSHY---GVYQIKLCVEGKWEVIIVDDF : 255
CLP-10 : RPSQIRTKDGTSCAWSVFNNPQ-----PSDIEQGNLVGDCWLLSAMALIAEKPEVLDAIIPKKQ--------YSKH---GVYQVKVCVEGKWEVIIVDDF : 202

CAPN1 : LPIKDGK-LVFVHSAEGNEFWSALLEKAYAKVNGSYEALSGGSTSEGF----EDFTGGVTEWYELR-------KAPS-DLYQIILKALERGSLLGCSIDI : 254
CAPN2 : LPTKDGE-LLFVHSAEGSEFWSALLEKAYAKINGCYEALSGGATTEGF----EDFTGGIAEWYELK-------KPPP-NLFKIIQKALQKGSLLGCSIDI : 244
CLP-4 : LPTVNNQ-LYYLHSADNTEFWSALVEKAYAKLHGGYENLDGGTTAEAL----EDFTGGLTEYFDLR-------KSEKAAVLAALVKGMEMGSLFGCSIDA : 435
CLP-7 : LPTVDGR-LCYMRSQENNEFWSALLEKAYAKLYGGYENLDGGSTAEAL----EDFTGGLTEFFDIS-------KGDKSTTLAMLVRGMQMGSLFGCSIDA : 505
CLP-1 : LPTSNGE-LLYMHSASNNEFWSALLEKAYAKLFGSYEALKGGTTSEAL----EDMTGGLTEFIDLK-------NPPR-NLMQMMMRGFEMGSLFGCSIEA : 510
CLP-3 : LPTVNGT-LCYMSSPGGSEFWGPLLEKAYAKLLGTYEHLNDPYTEQALWQASASFTGGLTQWFDIF-------MADEMAVLAMIMRGVQMGSLIVC-LNI : 413
CLP-6 : LPTVNGK-LIYMSSQDGDECWGPLLEKAYAKLYGTYEHLDGGTTTEAL----EDFTGGLTEFYDLT-------STDKTMILAMIMKGMQMGSLFACSIDP : 352
TRA-3 : LPTRDGK-LLFARSKTPNEFWSALLEKAFAKLYGCYENLVGGHLSDALQ----DVSGGVAETLHVRKFLKDDPNDTELKLFNDLKTAFDKGALVVAAIAA : 234
CLP-8 : FPYTTDG--IRIGATSGYQIWAALIEKALVKECGNYKGIHGFQSLSAFS----ALTGSPVLLIPVAK-----MFNNLRKYWKTLMEFRNNQYPMACGTLN : 268
CLP-2 : FPVDENNRMMCSQTENKGELWVSLLEKAYMKVMGGYDFPGSNSNIDLN-----ALTGWIPERIELSDTS----KADPDEVFRKLFDRFHRGDCLITLATG : 446
CLPR-1 : LPQKSNS-FERYAYMVKKQIWVAFIEKGFAKIRKSYEKLSGGVAGIALQ----QLTGAMTFSVFME-----KFNNDENRVWEFIQENRNSKFILTVSTPT : 114
CLPR-2 : VPSSSAS-YEIFTPMVRKQAWAALIQKAFAKLGGSYAKLHGGFADIAFL----QLTGSFTSTYYLN-----KLSSD-NDIWDFILSMQKSKFLVTACSTY : 113
CLPR-3 : --MNKQS-IEEYAWMIGKQTWAAFIEKGFAKLFGSYKSLSAKPIDVAFR----KLTGAFSKNYELR-----SFKNC-DAVWDMIVEAHLAGFLLCTSKLS : 87
CLPR-4 : --MNKQS-IEEYAWMIGKQTWAAFIEKGFAKLFGSYKSLSAKPIDVAFR----KLTGAFSKNYELR-----SFKNC-DAVWDMIVEAHLAGFLLCTSKLS : 87
CLP-9 : FPCYSKTNSIAMAVGRRNQLWVPLIEKAMAKVLGSYSKLHGASLAQGLS----MLTGASCVNYNCPPIP--SSVDDVDTFWAQLVSSKESGFLMCCHCGA : 349
CLP-10 : FPCYRNTNSIAIAVGRRNQLWVPLIEKAMAKVLGSYSKLHGASLARGLS----MLTGASCVRYKCPQTL--KIDEEVDTFWAQLVSSQESGFLTCCHCGP : 296

**H N**

CAPN1 : -SSVLDMEAITFKKLVKGHAYSVTGAKQVNYRGQ-------------VVSLIRMRNPWG-EVEWTGAWSDS---SSEWNNVDPYERDQLRVKME-----D : 331
CAPN2 : -TSAADSEAITFQKLVKGHAYSVTGAEEVESNGS-------------LQKLIRIRNPWG-EVEWTGRWNDN---CPSWNTIDPEERERLTRRHE-----D : 321
CLP-4 : -DANI-KEAQLRNGLVCGHAYSITAIHSITYYGE-------------DTTLLRLRNPWGNEKEWNGAWSDG---SSEWSKIDEATKKQIDVQFAR----D : 513
CLP-7 : -DENV-KEAQLTNGLVRGHAYSITAIQTVNTYGG-------------QVPLLRIRNPWGNSKEWNGAWSDG---SSEWSQVDPQQREQMGVQFAK----D : 583
CLP-1 : -DPNV-WEAKMSNGLVKGHAYSITGCRIVDGPNG-------------QTCILRIRNPWGNEQEWNGPWSDN---SREWRSVPDSVKQDMGLKFDH----D : 588
CLP-3 : -DVNE-KGVRQKNGLIKGYGYCITGVNLMETEWE-------------KAPLIRIRSPWG-KVKWKGDFCYR---SHRWFGLDKEKRE--SFRIKE----D : 488
CLP-6 : -DPRE-KEAQLANGLVRGHAYSVTGVHTVETDKE-------------KVALLRIRNPWG-DTEWNGDWSDK---SSLWEQVDQEQREKMEFRIKE----D : 429
TRA-3 : -RTKEEIEESLDCGLVKGHAYAVSAVCTIDVTNPNERSFTFIMGSKRKQNLIRLQNPWG-EKEWNGAWSDD---SPEWQNVSASQLSTMGVQPANSDSDD : 330
CLP-8 : -REVN--------GILPQHAYTIMDIVERDG-----------------HKLLLLRNPSG-GSVWTRNWSKE------WEWWPENMKDLLEGMIRG----- : 330
CLP-2 : -KMTEDMQK--RSGLVETHAYAVIDIRCVETKR-----------------LLKVKNPWT-HSRWKGNFSDKD--KVNWTAKMKNALAFDPEVAAEK--DD : 521
CLPR-1 : EEESEKKQLLEEYGIRDCHEYSVLDAQVYMG-----------------HRLILLAGSGP-FGKPKSVRRWG---HLPSYKEIREDWCAVDLGFSE----F : 190
CLPR-2 : EEGSEEWDIFLKNQISPNHGHGYTEMGSFTE-----------------IRWQVPIGKNI-LINYDSEK------CLCGN--------IFDYVLST----T : 178
CLPR-3 : LDEESAEYIFNSTGIKQNHAYAILNSVVFEN-----------------HRLVQIGNTHA-CDKWKELHKEWNSLHYFYNKFSSKLPRRPYISHLD----D : 165
CLPR-4 : LDEESAEYIFNSTGIKQNHAYAILNSVVFEN-----------------HRLVQIGNTHA-CDKWKELHKEWNSLHYFYNKFSSKLPRRPYISHLD----D : 165
CLP-9 : FENVVAEAEFKAMGLLTNHAYSILDVIYEQG-----------------YRLLRIRNPWG-QFVWNGKWSDG-----WPGWPIGMKQKFLNQRKDE----T : 422
CLP-10 : FESEEAEAEFKALGLVTSHAYSILDVVHEQG-----------------HRLLRIRNPWG-QFVWNGKWSDG-----WLEWPIDMKRALLDKRSDE----T : 369

CAPN1 : GEFWMSFRDFMREFTRLEICNLTPDALKSRTIR--------------KWNTTLYEGTWRRGS-TAGGCRNYPATFWVNPQFKIRLDETDDPDDYGDRESG : 416
CAPN2 : GEFWMSFSDFLRHYSRLEICNLTPDTLTSDTYK--------------KWKLTKMDGNWRRGS-TAGGCRNYPNTFWMNPQYLIKLEEEDEDEEDG--ESG : 404
CLP-4 : GEFWMSFEDFFSNFTQMEVCNLTAEIFDEIAEMTGVNRATETVEEEHQWHEIMEDGEWSSKKGTAGGCNNNPSTYPKNPQFSTFFTAPQSSIEADG---N : 610
CLP-7 : GEFWMSFDDFMTNFTQMECCNLSADVMDEISEMTGVEVHDK---QKHQWVEKSEDGEWNSRQGTAGGCQNN-DTYCNNPQYGTYFQVPMDSVEHDG---K : 676
CLP-1 : GEFWMSFDDFMRNFEKMEICNLGPDVMDEVYQMTGVKAAGM------VWAANTHDGAWVRNQ-TAGGCRNYINTFANNPQFRVQLTDS-DPDDDDE---L : 677
CLP-3 : GEFWMSLKDFMVEFTDVYCCNLSADTMHEVEKMTEVKVMEH---QSQQWIQASFEGEWSSRIGTAGGCDDHD-TFCTNLQYEIHFRATDSYDSNH----K : 580
CLP-6 : GEFWMCLDDFMAQFANLDCCNLSADVMHEITEMTEIEVMEK---QSKQWIQKSADGEWCSRKGTAGGCSNNENTFCTNPQYETYFRAT-SPSSND----K : 521
TRA-3 : GDFWMPWESFVHYFTDISLCQLFNTSVFSFSRSYDEQIVFS--------EWTTNGKKSGAPDDRAGGCHNFKATFCNNPQYIFDIPSP-----------N : 411
CLP-8 : -SFWISWDDFLNVFCSIYVCRHR---------------------------SNWFAYYAKLVLKYPEDDAFPAIDIKVTEKCMVCISAVDDWISREVLLKT : 402
CLP-2 : GIFWIDYESVRHFFDVIYVNWNAD--------------------------LFPFKSVYHATWTQDTGPIRDVYTVGENPQYTLTVNLNQK---------- : 585
CLPR-1 : GTFWIDMSELFQYFEYVTVCQYR---------------------------EKWKEIRIRRN-VVANTKNTE----------------------------- : 233
CLPR-2 : SIHWVDLHILSRFFSCFEICHYR---------------------------EGWKEIRFRKT-LVPKNGKTEILKLFLKTRCELVIEVIRR---------- : 240
CLPR-3 : MSFWMDIDQYCEHFSSLTVCEYR---------------------------KDWKEIRFRQTNDLKQSRETEALRMTVDRRCELVVEVTDR---------- : 228
CLPR-4 : MSFWMDIDQYCEHFSSLTVCEYR---------------------------KDWKEIRFRQTNDLKQSRETEALRMTVDRRCELVVEVTDR---------- : 228
CLP-9 : GAFWMDLEDFVARFASVTVCKLR---------------------------MDWSELRVTHK--VGGHSDTALQIVITDT-CEVSVTAFQKGAFNKKDNLN : 492
CLP-10 : GAFWMDLQDFVKRFASVTVCKLR---------------------------MDWSELRATQE--VGRHADSALQIVITEQLCEVSVTAFQKGSFSKEDNLT : 440

CAPN1 : CSFVLALMQKHRRRERRFGRDMETIGFAVYEVPPELVGQPAVHLKRDFFLANASRARSEQFINLREVSTRFRLPPGEYVVVPSTFEPNKEGDFVLRFFSE : 516
CAPN2 : CTFLVGLIQKHRRRQRKMGEDMHTIGFGIYEVPEELSGQTNIHLSKNFFLTNRARERSDTFINLREVLNRFKLPPGEYILVPSTFEPNKDGDFCIRVFSE : 504
CLP-4 : VTVIVAVLQKYRRELRSKGKDVLPIGVSIYSLGAEGT--ARSPLTAQFFSQNRPIARTTVFVNTREVTVRFRVPPGQYVIVPCTFDAYDDAEFLLRVYAN : 708
CLP-7 : CTVIGAVLQKYRRELRTKGLDNLPIGFSVYKADGSGQ--AIHDVSGQ-----KPIARTKVFINMREVTVRFRVPPGQYVIVPCTFDAHDDASFLLRIFSN : 769
CLP-1 : CTVIFAVLQKYRRNLKQDGLDNVPIGFAVYDAGNN-----RGRLSKQFFAANKSAMRSAAFINLREMTGRFRVPPGNYVVVPSTFEPNEEAEFMLRVYTN : 772
CLP-3 : CTIIAALFQKNRDHCVYKGLELFLIGLLVYEMPGP-----NEKVTPEMVKSQTPIADSKLFKDSREANIRFTVPLGHYVIVPSTYDPDQDGEFLLRIFTN : 675
CLP-6 : CTVIAAVFQKYRRNQLHRGLDMLQIGLSVYKMSGP-----NEKVTAEMMKTHAPIASTKLFVDYREAVVRFTVPPGYYVIVPCTFEPNHDAEFLLRTFSN : 616
TRA-3 : CSVMFALIQNDPSEGLKKREPFVTIGMHVMKVENNRQ--------YRVHTAMHPIAISDYASGRSVYLHLQSLPRGRYLLIPTTFAPKEQTLFMLRVYSD : 503
CLP-8 : VEFQMPKYVQRYSWIAVHKINGIFSDNKKVVACEIINDSTQDIDLEPGIYTITVIYLNDWSLDKRDISIHSSRPISVNEGFSRTRKDSVHIKYIVDKFGQ : 502
CLP-2 : AAAVWILLTRHITAIDDFAVNKEFITLIVYETGQKIYIPSNPQPISDGVRINSPLYLCQLSNK--------KPGITKYTLVVAQYEKTNTINYSLRVFST : 677
CLPR-1 : ---------------------------------------------------------------------------------------------------- : -
CLPR-2 : -KEALRLEAEQLRNRCILFCIVYSATADNKCDKILMI---THAYDLNLELTPGTLDPGTYLISFVILEDYDELELDWVIRR------------------- : 317
CLPR-3 : -VLAKIWVTRNRDTETTCSTNQDKIGLISAVEKNWGS---NPDKPNQKTLKRSTISDNSCEPVAKKPEFDFKTFMQELERYLACDDEVTPPYEFWTDPLN : 324
CLPR-4 : -VLAKIWVTRNRDTEVSFVKQDVK---------------------------------------------------------------------------- : -
CLP-9 : DLMVCVHRISGDRRIGELIEMSSRISDNHFTIDEFFL---APGEYQIVCHSQSSLLTNKKGVVNMVVHTRYPIFGEYIPMSPRTRMESLHHVIIKEGDIV : 589
CLP-10 : DLMVCVHKISEDGRIGELIKMSYEIADNHFTIDEFFL---PRGVYQVVCHSPHSLVTNTTGVVNIIVHTRFPIFGESVPMSPLTRLESLHRVIIEEGVVQ : 537

CAPN1 : KSAGTVELDDQIQANLPDEQVLSEEEIDENFKALFRQLAGEDMEISVKELRTILNRIISKHKDLRTKGFSLESCRSMVNLMDRDGNGKLGLVEFNILWNR : 616
CAPN2 : KKADYQAVDDEIEANL-EEFDISEDDIDDGFRRLFAQLAGEDAEISAFELQTILRRVLAKRQDIKSDGFSIETCKIMVDMLDSDGSGKLGLKEFYILWTK : 603
CLP-4 : GTLKSSLL-------------------------------------------------------------------------------------------- : -
CLP-7 : AEFQTTRLR------------------------------------------------------------------------------------------- : -
CLP-1 : GFIESEEL-------------------------------------------------------------------------------------------- : -
CLP-3 : VDFDKT------------------------------------------------------------HALSNG---------------------------I : 688
CLP-6 : VEFDKTDMHFIWKSAVFLCALLAINFPLTKSSIDLHSHEKVQISINNTSGPRKQVRIRFPEFIISGHIFCNGAPKRWLHPQLSTPKNPDEYFNIVTTDAA : 716
TRA-3 : EHIHFSPLTKHAPKLGLLKCKSAQSVTRLTIHGVDFNSASTGTHNVYAILKDSRKSFRTKTLSGVKSIQWDEQFLFHKSKNRQQYKIEVWEDRKMARDHL : 603
CLP-8 : EIVKEQKDKMSIKKYTDNNFTFIAVVAWNFTYDQFLHAHLRYSITDEQFVSRSLEDKQTVDVIPPRRHQVLVVINLEAMPEIVDFPIDIDYKLSKDVDAT : 602
CLP-2 : TDVKLEPVQLPYSISKTTRGNWDGSDKYPIMKLTLHSKSDEIALFMELKAPKQFCVALEMKQNSSDRTVFLETKSSGAYRPGYTVLTLEKVPAGKYYVKI : 777
CLPR-1 : ---------------------------------------------------------------------------------------------------- : -
CLPR-2 : ---------------------------------------------------------------------------------------------------- : -
CLPR-3 : KSACPRLAKFAAQFFICPPGSAEVERFFSGAGQILSKYRKSLSPERFNMLCFLSKNIPLMNKRYRKRVNTEKKEHDAKKKGEDGYEDAEGNSDSDDDFLF : 424
CLPR-4 : ---------------------------------------------------------------------------------------------------- : -
CLP-9 : KNTNDGVVIRTLTRKFRGMIIMADNCLEKKYLHVGVDCSQSMNIQSSRGFLQVVDVVPPLCRQVLLVLSTIDDSAQYRVSNSLKTLVHRSKCLLPEMWYE : 689
CLP-10 : NIKLDGVVIRTLNQKFRGSITMVDNYMEQKYLHVKVDNSKSMNVQSSRGSLLIADVVPPRSRQVIAVLSTIDDCAEYKTANSYWLRTNKSTSLLPDW--- : 634

CAPN1 : IRNYLSIFRKFDLDKSGSMSAYEMRMAIESAGFKLNKKLYELIITRYSEPDLAVDFDNFVCCLVRLETMFRFFKTLDTDLDGVVTFDLFKWLQLTMFA : 714
CAPN2 : IQKYQKIYREIDVDRSGTMNSYEMRKALEEAGFKMPCQLHQVIVARFADDQLIIDFDNFVRCLVRLETLFKIFKQLDPENTGTIELDLISWLCFSVL- : 700
CLP-4 : -------------------------------------------------------------------------------------------------- : -
CLP-7 : -------------------------------------------------------------------------------------------------- : -
CLP-1 : -------------------------------------------------------------------------------------------------- : -
CLP-3 : GLICKSDG--------------------HLLTVP---------------------------------------------------------------- : -
CLP-6 : GMYFLTTGNHFDLDRSVIITVLHQCEMKHLHPEPCALPYYKTVIPFNSTSSSTTYIRRDLELSKMEAFSSTHCL------------------------ : 790
TRA-3 : LAQSVIIALIDNENRDTTLQLTDPRGTVIGTVSVTVSAFDDPMYL----------------------------------------------------- : -
CLP-8 : INGIKRGCHLPEISSSRFLEYVHREEIISLDDFIPEIEVLSKLN------------------------------------------------------ : -
CLP-2 : STYTAGDKGPFILRIDSTCKFDLEPIKL---------------------------------------------------------------------- : -
CLPR-1 : -------------------------------------------------------------------------------------------------- : -
CLPR-2 : -------------------------------------------------------------------------------------------------- : -
CLPR-3 : G------------------------------------------------------------------------------------------------- : -
CLPR-4 : -------------------------------------------------------------------------------------------------- : -
CLP-9 : AAISAPNAQHYPLLNTSSFDPIHSTVSVF--------------------------------------------------------------------- : -
CLP-10 : -------------------------------------------------------------------------------------------------- : -
