# Supplementary material for: The Atypical Calpains: Evolutionary Analyses and Roles in Caenorhabditis elegans Cellular Degeneration
Source: PLoS Genet. 2012 Mar 29;8(3):e1002602. doi: 10.1371/journal.pgen.1002602 (PMC3315469; doi:10.1371/journal.pgen.1002602)
Supplement: Protocol S1 — Protein blast and phylogenetic analyses. (DOC) [file pgen.1002602.s013.doc]

**Blast analyses and protein phylogeny**

Sequence similarity searches were performed using BLASTP and alignments were generated using ClustalW version 2.1 (<http://www.ebi.ac.uk/Tools/msa/clustalw2/>) or FSA. A maximum likelihood phylogenetic tree was generated using Phylip version 3.67 with 1000 iterations and results were displayed using TreeView. *C. elegans* and *C. briggsae* sequences used for these analyses can be obtained from Wormbase ([http://www.wormbase.org/](http://genome.cshlp.org/content/15/10/1402.long#ref-64), release WS225), and non-nematode sequences used in Figure 1 can be obtained from GenBank using the following accession numbers: HsCAPN1 (AAH75862.1), HsCAPN2 (NP_001739.2), HsCAPN3/p94 (AAI46650.1), HsCAPN5 (EAW75015.1), HsCAPN6 (NP_055104.2), HsCAPN7 (NP_055111.1), HsCAPN8 (NP_001137434.1), HsCAPN9 (NP_006606.1), HsCAPN10 (NP_075571.1), HsCAPN11 (EAX04252.1), HsCAPN12 (NP_653292.2), HsCAPN13 (NP_653176.2), HsCAPN14 (NP_001138594.1), HsCAPN15 (NP_005623.1), DmCALPA (NP_001097378.1), DmCALPB (NP_524016.4), DmCALPC (AAF48591.2), DmCALPD (AAF50826.4), and PgTPR1 (AAA25652.1).

Figure 2 was generated by analysing the following sequences, which are listed with their GenBank accession numbers; *P. pacificus* sequences were obtained from WormBase.org: *S. Mansoni*: AAA29858.1, XP_002577797.1, XP_002579968.1, XP_002579967.1, XP_002571860.1, XP_002574256.1, XP_002578116.1, XP_002579451.1, XP_002574979.1, XP_002573024.1; *N. vectensis*: XP_001640696.1, XP_001632256.1, XP_001640599.1, XP_001626960.1, XP_001630054.1, XP_001637860.1, XP_001636091.1; *H. magnipapillata*: XP_002167620.1, XP_002154752.1, XP_002154722.1, XP_002163326.1, XP_002168617.1, XP_002164418.1; *T. Adhaerens*: XP_002108316.1, XP_002109313.1, XP_002107604.1, XP_002112715.1, XP_002111255.1, XP_002112373.1; *A. mellifera*: NP_001153877.1, XP_001120458.2, XP_624008.1, XP_001121978.2; *C. intestinalis*: XP_002120374.1, XP_002124635.1, XP_002124387.1, XP_002120263.1, XP_002121253.1, XP_002124575.1; *D. rerio*: NP_001138267.1, NP_998624.1, NP_001013519.1, CAN88624.1, CAM56363.1, NP_001003485.1, NP_001003501.1, NP_001004571.1, XP_001345114.2, XP_688575.3, XP_002663893.2, NP_001128580.1, XP_698873.3; *P. pacificus*: PP36964, PP39813, PP40628, PP45092, PP44265, PP44428; *A. queenslandica*: XP_003388253.1, XP_003388061.1, XP_003383174.1, XP_003384068.1.

Proteolytically inactive calpains were identified by obtaining 1234 protein sequences containing the calpain catalytic domain (signature SAAS022684_004_001783) from the UniProt database and removing protein fragments and splice variants from this list. The remaining proteins were aligned using FSA and proteins lacking one or more catalytic C, H or N residues were identified.
