# Supplementary material for: The Atypical Calpains: Evolutionary Analyses and Roles in Caenorhabditis elegans Cellular Degeneration
Source: PLoS Genet. 2012 Mar 29;8(3):e1002602. doi: 10.1371/journal.pgen.1002602 (PMC3315469; doi:10.1371/journal.pgen.1002602)
Supplement: Protocol S2 — Oligonucleotide sequences. (DOC) [file pgen.1002602.s014.doc]

**Table S6. Oligonucleotide sequences.**

| **Primer** | **Sequence (5’ – 3’)** |
| --- | --- |
| PK315 | CAGTTTCATACACAATAAGAGTG |
| PK316 | CGCTTACTCAAAGTGAAGAAC |
| PK330 | GATCCCTATTCGGTTGCTCC |
| PK331 | CTGCATCCTCCAGCTGTTTG |
| PK332 | GAGCATCAGAGCCAGCAATG |
| PK333 | GATTCTCAACAGAAACTCGCC |
| PK334 | CATTTGCGTCGAAGACGTCC |
| PK335 | CTCCAAAGTCGAGCTCTTCC |
| PK336 | CTCGTGAGATGCTCTTCATTC |
| PK337 | GCTATACCTTATCCGTCTCC |
| PK338 | GTCGTCGGACAAATCGGATC |
| PK339 | GACATCTCTGACGTTCCTGC |
| PK340 | CAGAGGAATGATCATAATGGC |
| PK341 | GAAGACGTGTTCAACAGAGG |
| PK342 | CTCCAAGTTTATCCTGACGG |
| PK343 | GAGCTCACTCATATCAATCC |
| PK344 | GTTGATGATTTCTTCCCCTGC |
| PK345 | GTCCACAATGGCAACACGTC |
| PK346 | CTCTTACTGGATCACCTGTG |
| PK347 | GATCCTCGAATCATTCCTTCC |
| PK503 | AAGCTTCGTTCATTGCTATGTTTTCGTTG |
| PK474 | GGATCCTTTGATTCCGTCGGCTTTTGC |
| PK527 | GCTAGCGTGTGTGAAGTTCAAATACAG |
| PK528 | CCCGGGACGCTGAAAACAGGAAATACTATTTC |
| PK477 | GTCGACCATATACACGCATTCACTGC |
| PK529 | TCTAGATTAGAAATTATTTCGTTTTTTACTGTTAAG |
| PK530 | AAGCTTCTCGTGTTCGTGCTTTGATTG |
| PK531 | GGATCCTGGTCTGAAAAATGTTGAAAATTTG |
| PK481 | AAGCTTCATTAACATGCAAGCTGTGG |
| PK482 | GGATCCTTAAAATGGATGATGGGAAGATTTG |
| PK553 | AAGCTTGGCCAGCATAGTCACTTCAG |
| PK584 | AAGCTTTTCGTCCTCCCGACTACACTC |
| PK485 | AAGCTTGTATTATCCTCCGTTTTGGTG |
| PK486 | GGATCCTGTTCAGCAGATTTCACAAG |
| PK487 | GCTAGCATGACACGGTCCGAAAAGACCC |
| PK488 | GATATCTTAAAGGTACATAGGATCATC |
| PK504 | GAATTCGCTAGCTTAGGCGCCGGTGGAGTGGCGGC |
| PK505 | ACCGGTAGAAAAAATGGCCTCCTCCGAGGACGTC |
| PK541 | GCTAGCATGGCTGACGATGAGGAAG |
| PK542 | GCTAGCAAGCTTTTACAGTTCCTCCGACTCG |
| PK547 | GCTAGCATGGCCAACCATGCAAAACTC |
| PK548 | GATATCAAGCTTTTACAGCAGGCTAGATTTCAAAG |
| PK551 | GCTAGCATGTCCGACGAGGAGGAGTAC |
| PK552 | GGTACCAAGCTTTTAGCGAAGACGGGTCGTCTG |
| PK728 | TGAAGCTTATGGCTGACGATGAGGAAGAAA |
| PK768 | AACTCGGAGATGCTTGGCTCTTGGC |
| PK769 | GCCAAGAGCCAAGCATCTCCGAGTT |
| PK781 | TCTAGACAGTTCCTCCGACTCGATAAAT |
| PK775 | AAGCTTAGTAAAAGAAGTAGAATTTTATAG |
| PK776 | GCTAGCATATGCTGTTGTAGCTGAAAATTTTG |
| PK792 | ATCAAGCTTCGGAACAGTCGAAAGTCGG |
| PK793 | ATCGCTAGCCTGTAATGAAATAAATGTGACGC |
| PK820 | CACTCGAGATTTGATGTTATTC |
| PK821 | CGTCCTCGGAGGAGGCCATACCCAGTTCCTCCGACTCGATAAAT |
| PK822 | ATTTATCGAGTCGGAGGAACTGGGTATGGCCTCCTCCGAGGACG |
| PK823 | TCGCTAGCTTAGGCGCCGG |
| PK1062 | CAATGTCTTGAGAGCAAACGAC |
| PK1063 | GGTGGACGTTTCGAGAAGAA |
| PK1066 | TCACGTGTATGTGCCTTTGAG |
| PK1067 | GAGTTGTTGGCTGGAAACTG |
| PK1074 | ATTTCCAGCAGGAACGTCAG |
| PK1084 | GGTCGCAGGTGGATGCGGTC |
| PK1085 | CTCGTTCGGCGGTCAGCATG |
| PK1087 | GGAGTCGTTGGCCGGAAATTG |
| PK1095 | CAAGCAGAAGTTCGACACCA |
| PK1096 | GGATCCGTATTGGATCGAGA |
| PK1097 | GCTTGCCGTGGAATTATTTG |
| PK1098 | AGGAAGATTTGGAAGCGTCA |
| PK1099 | TGCCATCCAGAGAATCAAGC |
| PK1100 | CTGGGACGTACTTGGCCTTA |
| PK1101 | GGTTCTCGGAGTTCAACCAG |
| PK1102 | ACGGTTGAGCCAAAATGAGA |
| PK1103 | TTGGAGCTACTTACCACAGCAA |
| PK1104 | TTCCTCCGATGGTAATGTCC |
| PK1105 | ACCAACCTGCAAGACCAACT |
| PK1106 | CCTGATCCGTATTGGATGGT |
| PK1107 | TTGTTTCGCCAGAACAAGC |
| PK1108 | CGGCTTCCTGAGACTTTTTG |
| PK1109 | AGCGATCATAGAGGTGAACGA |
| PK1110 | TCTTGCAGACATCGGAACTG |
| PK1113 | TATACCAAGCAACCCGCAAC |
| PK1114  PK1117  PK1132 | ACTGAGCAACAACCAACGTG  cagggatcactggagccac  CGAAAACTTCTGTAACTCAGG |
| clp-4_f2 | CGAAGTCCGGTTGATGATTT |
| clp-4_r2 | GGAGCCTCTTGTTTGTCGTC |
| clp-6_f2 | ACTCTTCTGGACGTGGCCTA |
| clp-6_r2 | CCCGACAAGAGATTCGAGAG |
| clp-8_f2 | TCGGTGATCACGAGAATGAA |
| clp-8_r2 | CAGATTTGGATCTTTGGCAAT |
| clp-9_f2 | TCCTGCAATCTTCATTTGTCA |
| clp-9_r2 | TCGTTAAAGGCTTTTGAGAACC |
| clp-10_f2 | TCTTTGAAACAGGCACAGGA |
| clp-10_r2 | GGAGAATTGAATAACGTGCTGA |
| clpr1_f2 | ATGTCGTACACACCGCTTCC |
| clpr1_r2 | TCTCCATTAATCAAAAGCTTCAA |
