# Supplementary material for: The Atypical Calpains: Evolutionary Analyses and Roles in Caenorhabditis elegans Cellular Degeneration
Source: PLoS Genet. 2012 Mar 29;8(3):e1002602. doi: 10.1371/journal.pgen.1002602 (PMC3315469; doi:10.1371/journal.pgen.1002602)
Supplement: Protocol S3 — Molecular biology and gene cloning methods. (DOC) [file pgen.1002602.s015.doc]

**Molecular biology and gene cloning**

RNAi constructs were generated by PCR amplifying 250 – 500 bp exon-rich DNA fragments from N2 genomic DNA corresponding to *clp-1* to *clp-10* and *clpr-1*. PCR was performed with the following primer pairs: PK330/PK331 (*clp-1*), PK315/PK316 (*clp-2*), PK332/PK333 (*clp-3*), PK334/PK335 (*clp-4*), PK336/PK337 (*clp-6*), PK338/PK339 (*clp-7*), PK346/PK347 (*clp-8*). PK340/PK341 (*clp-9*), PK344/PK345 (*clp-10*) and PK342/PK343 (*clpr-1*). All constructs generated by PCR were verified by DNA sequencing.The *tra-3*/*clp-5* RNAi construct was generated by cloning a 420 bp *tra-3* cDNA fragment into L4440.

Transcriptional expression reporters were constructed using the promoterless host vector pPJ-1 (*nls::mrfp)*, which was generated by excising the GFP(S65C) fragment from vector pPD112.56 (kindly provided by Andy Fire) and replacing it with an mRFP sequence amplified with primers PK504 and PK505 using the pmRFP_C1 vector. Subsequently, promoter sequences consisting of ~3 kb of sequence upstream from calpain translational start sites were PCR amplified from N2 genomic DNA and inserted in pPJ-1. PCR was performed with the following primer pairs: PK503/PK474 (*clp-1*), PK527/PK528 (*clp-2*), PK477/PK529 (*clp-3*), PK530/PK531 (*clp-4*), PK481/PK482 (*tra-3/clp-5*), PK553/PK584 (*clp-6*) and PK485/PK486 (*clp-7*).

The *clp-1p::gfp* translational fusion construct (pPJ-83) was created by amplifying a 2349 bp *clp-1* cDNA fragment from the clone yk1317a01, which carries *clp-1* splice variant C06G4.2a.1 (kindly provided by Yuji Kohara), using primers PK728 and PK781; the resulting fragment was cloned upstream of the GFP(S65C) insert in vector pPD95.77 (kindly provided by Andy Fire). The *clp-1* promoter was then PCR amplified from *clp-1p::nls::mrfp* (pPJ-2) and cloned upstream of the *clp-1* cDNA insert in pPD95.77.

Reporters driven from the *hsp-16.64* heat shock promoter were generated by inserting calpain cDNA sequences into the vector pPD49.83 (kindly provided by Andy Fire). cDNAs were obtained, as follows: *clp-1* cDNA was PCR amplified from yk1317a01 using primers PK541 and PK542 (pPJ-106); *clp-2* cDNA was released from pPK264 (originally from yk3b10) by digestion with NheI and EcoRV (pPJ-11); *clp-4* cDNA was amplified by RT-PCR from N2 mRNA using primers PK547 and PK548 (pPJ-12); *hsp-16.64p::tra-3/clp-5*, pPK247 was made in a previous study; *clp-7* cDNA was released from yk1733g09 (kindly provided by Yuji Kohara) by digestion with XbaI and HpaI (pPJ-13). Heat shock driven reporters were activated by incubating transgenic strains daily at 33°C for an hour beginning at the L1 larval stage. Day 2 adults were scored for potential phenotypes.

*unc-54* promoter driven cDNA expression constructs were generated by cloning cDNA sequences in the vector pPD30.38 (kindly provide by Andy Fire), which carries the *unc-54* promoter. cDNAs were obtained, as follows, to create the indicated plasmids: *clp-1* cDNA was released from pPJ-106 by digestion with NheI (pPJ-107); *clp-2* cDNA released from pPJ-11 by digestion with NheI and EcoRV (pPJ-47); *clp-4* cDNA was released from pPJ-12 by digestion with NheI and EcoRV (pPJ-48); *tra-3* cDNA was amplified from pPK247 using primers PK487 and PK488 (pPJ-97); and *clp-7* cDNA was amplified from pPJ-13 using primers PK551 and PK754 (pPJ-92). Proteolytically inactive *unc-54p::clp-1*(C371A) (pPJ-108) was created from pPJ-107 using primers PK768 and PK769 with the QuickChange site directed mutagenesis kit (Stratagene), as directed by the manufacturer.

The *unc-119p* expression construct (pPK675) was created by amplifying an *unc-119* promoter sequence from N2 genomic DNA using primers PK775 and PK776. *unc-119p::clp-1* (pPJ-74) was created by releasing *clp-1* cDNA from pPJ-109 by digestion with NheI and ligation into pPK675.

The *unc-47p* expression vector (pPJ-91) was created by amplifying a 1194 bp *unc-47* promoter from N2 genomic DNA using primers PK792 and PK793. *unc-47p::clp-1* (pPJ-92) was created by releasing *clp-1* cDNA from pPJ-109 by digestion with NheI and ligation into pPJ-91.

The *unc-54p::clp-1::mrfp* construct (pMC-7) was created using insertional PCR. Primers PK820 and PK821 amplified *unc-54p::clp-1* from pPJ-107 and primers PK822 and PK823 amplified *mrfp* from pPJ-1. PCR products were mixed and full length *unc-54p::clp-1::mrfp* was amplified using PK820 and PK823, subcloned into pBSKSII(+), digested with XhoI and SpeI and ligated into pPJ-107 to create pMC-7. The *unc-54p::clp-1(C371A)::mrfp* construct was created by releasing a *clp-1* cDNA fragment from pPJ-108 by digestion with MluI and NsiI and ligation into pMC-7.
