# Supplementary material for: The Atypical Calpains: Evolutionary Analyses and Roles in Caenorhabditis elegans Cellular Degeneration
Source: PLoS Genet. 2012 Mar 29;8(3):e1002602. doi: 10.1371/journal.pgen.1002602 (PMC3315469; doi:10.1371/journal.pgen.1002602)
Supplement: Table S1 — Eukaryotic catalytically inactive calpains. (DOC) [file pgen.1002602.s016.doc]

**Table S1: Eukaryotic catalytically inactive calpains.**

| **UniProtKB Protein ID** | **Gene Name** | **Species** | **Kingdom** | **Animal phylum^a^** |
| --- | --- | --- | --- | --- |
| F0VZJ4_9STRA | AlNc14C2G332 | Albugo laibachii Nc14 | Fungi | NA |
| F0W3B3_9STRA | AlNc14C12G1490 | Albugo laibachii Nc14 | Fungi | NA |
| F0WE76_9STRA | AlNc14C72G4932 | Albugo laibachii Nc14 | Fungi | NA |
| F0WU21_9STRA | AlNc14C263G9844 | Albugo laibachii Nc14 | Fungi | NA |
| Q5TUE4_ANOGA | AGAP010395 | Anopheles gambiae | Animal | Arthropoda |
| E3WKY3_ANODA | AND_01192 | Anopheles darlingi | Animal | Arthropoda |
| E3WXG5_ANODA | AND_08871 | Anopheles darlingi | Animal | Arthropoda |
| E3XCT9_ANODA | AND_20429 | Anopheles darlingi | Animal | Arthropoda |
| F1KXD5_ASCSU | Unknown | Ascaris lumbricoides | Animal | Nematoda |
| F1KY41_ASCSU | Unknown | Ascaris lumbricoides | Animal | Nematoda |
| A1CRW2_ASPCL | ACLA_031160 | Aspergillus clavatus | Fungi | NA |
| F0Y5U5_9STRA | AURANDRAFT_63204 | Aureococcus anophagefferens | Protist | NA |
| F4NTM9_BATDE | BATDEDRAFT_85291 | Batrachochytrium dendrobatidis JAM81 | Fungi | NA |
| F4P4B4_BATDE | BATDEDRAFT_35218 | Batrachochytrium dendrobatidis JAM81 | Fungi | NA |
| C5K406_AJEDS | BDBG_09548 | Blastomyces dermatitidis | Fungi | NA |
| E1BJ18_BOVIN | CAPN6 | Bos taurus | Animal | Chordata |
| A6SA00_BOTFB | BC1G_09169 | Botrytis cinerea | Fungi | NA |
| A5PMP0_DANRE | capn2b | Brachydanio rerio | Animal | Chordata |
| E9QE31_DANRE | si:dkey-12h9.9 | Brachydanio rerio | Animal | Chordata |
| F1QQ89_DANRE | si:dkey-170m15.2 | Brachydanio rerio | Animal | Chordata |
| F1R632_DANRE | si:dkey-12h9.9 | Brachydanio rerio | Animal | Chordata |
| C3Y6N4_BRAFL | BRAFLDRAFT_74933 | Branchiostoma floridae | Animal | Chordata |
| C3Y6N9_BRAFL | BRAFLDRAFT_74938 | Branchiostoma floridae | Animal | Chordata |
| C3Y6P2_BRAFL | BRAFLDRAFT_74941 | Branchiostoma floridae | Animal | Chordata |
| C3ZHX2_BRAFL | BRAFLDRAFT_88755 | Branchiostoma floridae | Animal | Chordata |
| C3ZZL6_BRAFL | BRAFLDRAFT_132644 | Branchiostoma floridae | Animal | Chordata |
| A8PU52_BRUMA | Bm1_34510 | Brugia malayi | Animal | Nematoda |
| A8WMS0_CAEBR | CBG00485 | Caenorhabditis briggsae | Animal | Nematoda |
| O18165_CAEEL | W04A4.4 | Caenorhabditis elegans | Animal | Nematoda |
| Q6F6K4_CAEEL | F47F6.9 | Caenorhabditis elegans | Animal | Nematoda |
| Q9U247_CAEEL | Y53H1B.6 | Caenorhabditis elegans | Animal | Nematoda |
| Q9U2B2_CAEEL | clp-3 | Caenorhabditis elegans | Animal | Nematoda |
| Q9XU14_CAEEL | H25P06.4 | Caenorhabditis elegans | Animal | Nematoda |
| E3LMG6_CAERE | CRE_28132 | Caenorhabditis remanei | Animal | Nematoda |
| E3M575_CAERE | CRE_10859 | Caenorhabditis remanei | Animal | Nematoda |
| E3MBF3_CAERE | CRE_16029 | Caenorhabditis remanei | Animal | Nematoda |
| E3MBK7_CAERE | CRE_16054 | Caenorhabditis remanei | Animal | Nematoda |
| E3MBK8_CAERE | CRE_16055 | Caenorhabditis remanei | Animal | Nematoda |
| E3MBK9_CAERE | CRE_16056 | Caenorhabditis remanei | Animal | Nematoda |
| E3MBL1_CAERE | CRE_16058 | Caenorhabditis remanei | Animal | Nematoda |
| E3MBL2_CAERE | CRE_15929 | Caenorhabditis remanei | Animal | Nematoda |
| E3MBL3_CAERE | CRE_16059 | Caenorhabditis remanei | Animal | Nematoda |
| E3MBL4_CAERE | CRE_16060 | Caenorhabditis remanei | Animal | Nematoda |
| E3MBL7_CAERE | CRE_16063 | Caenorhabditis remanei | Animal | Nematoda |
| E3MQN5_CAERE | CRE_10418 | Caenorhabditis remanei | Animal | Nematoda |
| E3MW78_CAERE | CRE_22983 | Caenorhabditis remanei | Animal | Nematoda |
| E3N2T3_CAERE | CRE_20454 | Caenorhabditis remanei | Animal | Nematoda |
| E3N747_CAERE | CRE_11407 | Caenorhabditis remanei | Animal | Nematoda |
| E3NGV9_CAERE | CRE_06153 | Caenorhabditis remanei | Animal | Nematoda |
| E3NL90_CAERE | CRE_16491 | Caenorhabditis remanei | Animal | Nematoda |
| E3NM44_CAERE | CRE_04401 | Caenorhabditis remanei | Animal | Nematoda |
| E3NM47_CAERE | CRE_04402 | Caenorhabditis remanei | Animal | Nematoda |
| E3NRK6_CAERE | CRE_12429 | Caenorhabditis remanei | Animal | Nematoda |
| E3NW07_CAERE | CRE_16588 | Caenorhabditis remanei | Animal | Nematoda |
| E2AP24_9HYME | EAG_03627 | Camponotus floridanus | Animal | Arthropoda |
| PALB_YARLI | RIM13 | Candida lipolytica | Fungi | NA |
| C4Y0U3_CLAL4 | CLUG_01825 | Candida lusitaniae | Fungi | NA |
| PALB_KLULA | RIM13 | Candida sphaerica | Fungi | NA |
| E2RSU8_CANFA | CAPN9 | Canis lupus familiaris | Animal | Chordata |
| E9C0T3_9EUKA | CAOG_01723 | Capsaspora owczarzaki ATCC 30864 | Protist | NA |
| A8J614_CHLRE | FAP42 | Chlamydomonas smithii | Protist | NA |
| A9UVD6_MONBE | 31789 | Choanoflagellate | Protist | NA |
| C6K3U5_9TRYP | CDFL12A17_07 | Crithidia sp. ATCC 30255 | Protist | NA |
| C6K3U6_9TRYP | CDFL12A17_06 | Crithidia sp. ATCC 30255 | Protist | NA |
| C6K3U8_9TRYP | CDFL12A17_04 | Crithidia sp. ATCC 30255 | Protist | NA |
| C6K3U9_9TRYP | CDFL12A17_03 | Crithidia sp. ATCC 30255 | Protist | NA |
| E6RE93_CRYGW | CGB_L3100W | Cryptococcus gattii | Fungi | NA |
| Q16WJ3_AEDAE | AAEL009196 | Culex aegypti | Animal | Arthropoda |
| Q174B8_AEDAE | AAEL006960 | Culex aegypti | Animal | Arthropoda |
| Q17E40_AEDAE | AAEL003974 | Culex aegypti | Animal | Arthropoda |
| Q17E42_AEDAE | AAEL003967 | Culex aegypti | Animal | Arthropoda |
| Q17FB6_AEDAE | AAEL003476 | Culex aegypti | Animal | Arthropoda |
| B0WDU6_CULQU | CpipJ_CPIJ005039 | Culex pungens | Animal | Arthropoda |
| B0XDG9_CULQU | CpipJ_CPIJ017492 | Culex pungens | Animal | Arthropoda |
| E9G357_DAPPU | DAPPUDRAFT_313447 | Daphnia pulex | Animal | Arthropoda |
| E9GFW8_DAPPU | DAPPUDRAFT_303364 | Daphnia pulex | Animal | Arthropoda |
| B3N0T1_DROAN | GF19104 | Drosophila ananassae | Animal | Arthropoda |
| B3N0T2_DROAN | GF19093 | Drosophila ananassae | Animal | Arthropoda |
| B3NX55_DROER | GG17954 | Drosophila erecta | Animal | Arthropoda |
| B4JJC7_DROGR | GH12281 | Drosophila grimshawi | Animal | Arthropoda |
| CANC_DROME | CalpC | Drosophila melanogaster | Animal | Arthropoda |
| B4L863_DROMO | GI10979 | Drosophila mojavensis | Animal | Arthropoda |
| B4H111_DROPE | GL15828 | Drosophila persimilis | Animal | Arthropoda |
| B4H9X3_DROPE | GL16011 | Drosophila persimilis | Animal | Arthropoda |
| B5DKM0_DROPS | GA22814 | Drosophila pseudoobscura | Animal | Arthropoda |
| Q29GJ4_DROPS | GA17616 | Drosophila pseudoobscura | Animal | Arthropoda |
| B4IF60_DROSE | GM13427 | Drosophila sechellia | Animal | Arthropoda |
| B4R5S7_DROSI | GD17276 | Drosophila simulans | Animal | Arthropoda |
| B4M289_DROVI | GJ19444 | Drosophila virilis | Animal | Arthropoda |
| B4NCM1_DROWI | GK25057 | Drosophila willistoni | Animal | Arthropoda |
| B4PXR7_DROYA | GE17262 | Drosophila yakuba | Animal | Arthropoda |
| D7FQ17_ECTSI | Esi_0002_0104 | Ectocarpus siliculosus | Protist | NA |
| D7FSU2_ECTSI | Esi_0240_0008 | Ectocarpus siliculosus | Protist | NA |
| D7FTW8_ECTSI | Esi_0260_0039 | Ectocarpus siliculosus | Protist | NA |
| D7G3H8_ECTSI | Esi_0051_0063 | Ectocarpus siliculosus | Protist | NA |
| D8LK54_ECTSI | Esi_0028_0161 | Ectocarpus siliculosus | Protist | NA |
| D8LKI3_ECTSI | Esi_0030_0088 | Ectocarpus siliculosus | Protist | NA |
| D8LU43_ECTSI | Esi_0095_0032 | Ectocarpus siliculosus | Protist | NA |
| D8LU44_ECTSI | Esi_0095_0034 | Ectocarpus siliculosus | Protist | NA |
| D8LU45_ECTSI | Esi_0095_0035 | Ectocarpus siliculosus | Protist | NA |
| PALB_CRYNB | RIM13 | Filobasidiella neoformans | Fungi | NA |
| C7ZBJ4_NECH7 | NECHADRAFT_88447 | Fusarium solani subsp. pisi | Fungi | NA |
| C7ZF18_NECH7 | NECHADRAFT_86428 | Fusarium solani subsp. pisi | Fungi | NA |
| Q5GCP6_GECLA | CalpM | Gecarcinus lateralis | Animal | Arthropoda |
| E3Q4I4_COLGM | GLRG_01143 | Glomerella graminicola | Fungi | NA |
| A6R587_AJECN | HCAG_04795 | Histoplasma capsulatum | Fungi | NA |
| C0NS99_AJECG | HCBG_06029 | Histoplasma capsulatum | Fungi | NA |
| F0UUB3_AJEC8 | HCEG_08705 | Histoplasma capsulatum | Fungi | NA |
| CAN6_HUMAN | CAPN6 | Homo sapiens | Animal | Chordata |
| A8N4X4_COPC7 | CC1G_04674 | Hormographiella aspergillata | Fungi | NA |
| A8NAE6_COPC7 | CC1G_05897 | Hormographiella aspergillata | Fungi | NA |
| B7PCL2_IXOSC | IscW_ISCW002386 | Ixodes scapularis | Animal | Arthropoda |
| B7PEU5_IXOSC | IscW_ISCW017819 | Ixodes scapularis | Animal | Arthropoda |
| B7QCA9_IXOSC | IscW_ISCW012923 | Ixodes scapularis | Animal | Arthropoda |
| B7P5B6_IXOSC | IscW_ISCW015854 | Ixodes scapularis | Animal | Arthropoda |
| B0DRD8_LACBS | LACBIDRAFT_308000 | Laccaria laccata var. bicolor | Fungi | NA |
| B0DXY2_LACBS | LACBIDRAFT_313271 | Laccaria laccata var. bicolor | Fungi | NA |
| A4H9L6_LEIBR | LBRM_18_1160 | Leishmania braziliensis | Protist | NA |
| A4HAE6_LEIBR | LBRM_20_0290 | Leishmania braziliensis | Protist | NA |
| A4HBK9_LEIBR | LBRM_21_0160 | Leishmania braziliensis | Protist | NA |
| A4HFH5_LEIBR | LBRM_27_0600 | Leishmania braziliensis | Protist | NA |
| A4HFH6_LEIBR | LBRM_27_0610 | Leishmania braziliensis | Protist | NA |
| A4HFX3_LEIBR | LBRM_27_2140 | Leishmania braziliensis | Protist | NA |
| A4HID5_LEIBR | LBRM_30_1980 | Leishmania braziliensis | Protist | NA |
| A4HJ14_LEIBR | LBRM_31_0510 | Leishmania braziliensis | Protist | NA |
| A4HJ15_LEIBR | LBRM_31_0520 | Leishmania braziliensis | Protist | NA |
| A4HJ21_LEIBR | LBRM_31_0580 | Leishmania braziliensis | Protist | NA |
| A4HJ22_LEIBR | LBRM_31_0590 | Leishmania braziliensis | Protist | NA |
| A4HJ23_LEIBR | LBRM_31_0600 | Leishmania braziliensis | Protist | NA |
| A4HK78_LEIBR | LBRM_32_1060 | Leishmania braziliensis | Protist | NA |
| A4HNT8_LEIBR | LBRM_35_0900 | Leishmania braziliensis | Protist | NA |
| E9AIH1_LEIBR | LBRM_20_5380 | Leishmania braziliensis | Protist | NA |
| E9AIH3_LEIBR | LBRM_20_5400 | Leishmania braziliensis | Protist | NA |
| E9AIH4_LEIBR | LBRM_20_5410 | Leishmania braziliensis | Protist | NA |
| E9AIH6_LEIBR | LBRM_20_5430 | Leishmania braziliensis | Protist | NA |
| E9BDT7_LEIDO | LDBPK_181070 | Leishmania donovani BPK282A1 | Protist | NA |
| E9BES2_LEIDO | LDBPK_201230 | Leishmania donovani BPK282A1 | Protist | NA |
| E9BES3_LEIDO | LDBPK_201240 | Leishmania donovani BPK282A1 | Protist | NA |
| E9BES4_LEIDO | LDBPK_201250 | Leishmania donovani BPK282A1 | Protist | NA |
| E9BIY5_LEIDO | LDBPK_270510 | Leishmania donovani BPK282A1 | Protist | NA |
| E9BLU7_LEIDO | LDBPK_302040 | Leishmania donovani BPK282A1 | Protist | NA |
| E9BMG5_LEIDO | LDBPK_310410 | Leishmania donovani BPK282A1 | Protist | NA |
| E9BMG6_LEIDO | LDBPK_310420 | Leishmania donovani BPK282A1 | Protist | NA |
| E9BMG7_LEIDO | LDBPK_310430 | Leishmania donovani BPK282A1 | Protist | NA |
| E9BMG8_LEIDO | LDBPK_310440 | Leishmania donovani BPK282A1 | Protist | NA |
| E9BMG9_LEIDO | LDBPK_310450 | Leishmania donovani BPK282A1 | Protist | NA |
| E9BMH1_LEIDO | LDBPK_310480 | Leishmania donovani BPK282A1 | Protist | NA |
| E9BNK5_LEIDO | LDBPK_321020 | Leishmania donovani BPK282A1 | Protist | NA |
| E9BQI4_LEIDO | LDBPK_340300 | Leishmania donovani BPK282A1 | Protist | NA |
| E9BTE3_LEIDO | LDBPK_360840 | Leishmania donovani BPK282A1 | Protist | NA |
| A4HXY0_LEIIN | LINJ_18_1070 | Leishmania infantum | Protist | NA |
| A4HYN0_LEIIN | LINJ_20_1220 | Leishmania infantum | Protist | NA |
| A4HYW1_LEIIN | LINJ_20_1210 | Leishmania infantum | Protist | NA |
| A4HYW2_LEIIN | LINJ_20_1230 | Leishmania infantum | Protist | NA |
| A4HYW3_LEIIN | LINJ_20_1240 | Leishmania infantum | Protist | NA |
| A4HYW4_LEIIN | LINJ_20_1250 | Leishmania infantum | Protist | NA |
| A4I2N5_LEIIN | LINJ_27_0500 | Leishmania infantum | Protist | NA |
| A4I2N6_LEIIN | LINJ_27_0510 | Leishmania infantum | Protist | NA |
| A4I5N1_LEIIN | LINJ_30_2040 | Leishmania infantum | Protist | NA |
| A4I6E4_LEIIN | LINJ_31_0450 | Leishmania infantum | Protist | NA |
| A4I6E6_LEIIN | LINJ_31_0480 | Leishmania infantum | Protist | NA |
| A4I6F0_LEIIN | LINJ_31_0440 | Leishmania infantum | Protist | NA |
| A4I6K4_LEIIN | LINJ_31_0410 | Leishmania infantum | Protist | NA |
| A4I6K5_LEIIN | LINJ_31_0420 | Leishmania infantum | Protist | NA |
| A4I6K6_LEIIN | LINJ_31_0430 | Leishmania infantum | Protist | NA |
| A4I7R3_LEIIN | LINJ_32_1020 | Leishmania infantum | Protist | NA |
| A4I9J8_LEIIN | LINJ_34_0300 | Leishmania infantum | Protist | NA |
| A4ICQ6_LEIIN | LINJ_36_0840 | Leishmania infantum | Protist | NA |
| E9AHC1_LEIIN | LINJ_27_2520 | Leishmania infantum | Protist | NA |
| E9AD26_LEIMA | LMJF_27_0490 | Leishmania major | Protist | NA |
| E9AD27_LEIMA | LMJF_27_0500 | Leishmania major | Protist | NA |
| E9AD28_LEIMA | LMJF_27_0510 | Leishmania major | Protist | NA |
| Q4Q1Z8_LEIMA | LMJF_36_0780 | Leishmania major | Protist | NA |
| Q4Q3I0_LEIMA | LMJF_34_0280 | Leishmania major | Protist | NA |
| Q4Q5I5_LEIMA | LMJF_32_0970 | Leishmania major | Protist | NA |
| Q4Q6L9_LEIMA | LMJF_31_0440 | Leishmania major | Protist | NA |
| Q4Q6M0_LEIMA | LMJF_31_0430 | Leishmania major | Protist | NA |
| Q4Q6M2_LEIMA | LMJF_31_0410 | Leishmania major | Protist | NA |
| Q4Q6M3_LEIMA | LMJF_31_0400 | Leishmania major | Protist | NA |
| Q4Q6M4_LEIMA | LMJF_31_0390 | Leishmania major | Protist | NA |
| Q4Q790_LEIMA | LMJF_30_2040 | Leishmania major | Protist | NA |
| Q4QCK4_LEIMA | LMJF_21_0120 | Leishmania major | Protist | NA |
| Q4QCS5_LEIMA | LMJF_20_1210 | Leishmania major | Protist | NA |
| Q4QCS6_LEIMA | LMJF_20_1200 | Leishmania major | Protist | NA |
| Q4QCS7_LEIMA | LMJF_20_1190 | Leishmania major | Protist | NA |
| Q4QCS8_LEIMA | LMJF_20_1185 | Leishmania major | Protist | NA |
| Q4QCS9_LEIMA | LMJF_20_1180 | Leishmania major | Protist | NA |
| Q4QDT6_LEIMA | LMJF_18_1060 | Leishmania major | Protist | NA |
| E8NHG6_LEIME | LmxM_30_0440_1 | Leishmania mexicana MHOM/GT/2001/U1103 | Protist | NA |
| E8NHG7_LEIME | LmxM_30_0430_1 | Leishmania mexicana MHOM/GT/2001/U1103 | Protist | NA |
| E8NHQ2_LEIME | LmxM_30_0440b_1 | Leishmania mexicana MHOM/GT/2001/U1103 | Protist | NA |
| E9ARP3_LEIME | LMXM_18_1060 | Leishmania mexicana MHOM/GT/2001/U1103 | Protist | NA |
| E9ASJ8_LEIME | LMXM_36_0780 | Leishmania mexicana MHOM/GT/2001/U1103 | Protist | NA |
| E9AUQ7_LEIME | LMXM_20_1180 | Leishmania mexicana MHOM/GT/2001/U1103 | Protist | NA |
| E9AUQ8_LEIME | LMXM_20_1185 | Leishmania mexicana MHOM/GT/2001/U1103 | Protist | NA |
| E9AUQ9_LEIME | LMXM_20_1190 | Leishmania mexicana MHOM/GT/2001/U1103 | Protist | NA |
| E9AUR0_LEIME | LMXM_20_1200 | Leishmania mexicana MHOM/GT/2001/U1103 | Protist | NA |
| E9AUR1_LEIME | LMXM_20_1210 | Leishmania mexicana MHOM/GT/2001/U1103 | Protist | NA |
| E9AUX8_LEIME | LMXM_21_0120 | Leishmania mexicana MHOM/GT/2001/U1103 | Protist | NA |
| E9AYV2_LEIME | LMXM_27_0510 | Leishmania mexicana MHOM/GT/2001/U1103 | Protist | NA |
| E9B0X6_LEIME | LMXM_29_2040 | Leishmania mexicana MHOM/GT/2001/U1103 | Protist | NA |
| E9B1J0_LEIME | LMXM_30_0390 | Leishmania mexicana MHOM/GT/2001/U1103 | Protist | NA |
| E9B1J1_LEIME | LMXM_30_0400 | Leishmania mexicana MHOM/GT/2001/U1103 | Protist | NA |
| E9B1J2_LEIME | LMXM_30_0410 | Leishmania mexicana MHOM/GT/2001/U1103 | Protist | NA |
| E9B2M1_LEIME | LMXM_31_0970 | Leishmania mexicana MHOM/GT/2001/U1103 | Protist | NA |
| E9B4J6_LEIME | LMXM_33_0280 | Leishmania mexicana MHOM/GT/2001/U1103 | Protist | NA |
| E1GDD7_LOALO | LOAG_11178 | Loa loa | Animal | Nematoda |
| E1GN38_LOALO | LOAG_14606 | Loa loa | Animal | Nematoda |
| E2L7L8_MONPE | MPER_01884 | Marasmius perniciosus | Fungi | NA |
| E9E9C4_METAQ | MAC_06472 | Metarhizium acridum (strain CQMa 102) | Fungi | NA |
| E9EUD4_METAR | MAA_03633 | Metarhizium anisopliae | Fungi | NA |
| CAN6_MOUSE | Capn6 | Mus musculus | Animal | Chordata |
| D2UYF2_NAEGR | NAEGRDRAFT_56516 | Naegleria gruberi | Protist | NA |
| D2VGR9_NAEGR | NAEGRDRAFT_49404 | Naegleria gruberi | Protist | NA |
| D2VGX6_NAEGR | NAEGRDRAFT_79947 | Naegleria gruberi | Protist | NA |
| D2VVA0_NAEGR | NAEGRDRAFT_72942 | Naegleria gruberi | Protist | NA |
| A7RJ84_NEMVE | v1g197920 | Nematostella vectensis | Animal | Cnidaria |
| A7RWT3_NEMVE | v1g241226 | Nematostella vectensis | Animal | Cnidaria |
| F0VPI3_NEOCA | NCLIV_060540 | Neospora caninum Liverpool | Protist | NA |
| Q7S7N5_NEUCR | NCU01151 | Neurospora crassa | Fungi | NA |
| E4XHF7_OIKDI | GSOID_T00010932001 | Oikopleura dioica | Animal | Chordata |
| E4XMI4_OIKDI | GSOID_T00015378001 | Oikopleura dioica | Animal | Chordata |
| E4YD44_OIKDI | GSOID_T00021372001 | Oikopleura dioica | Animal | Chordata |
| F4WBI5_9HYME | G5I_02897 | Acromyrmex echinatior | Animal | Arthropoda |
| C1G8P9_PARBD | PADG_03635 | Paracoccidioides brasiliensis (strain Pb18) | Fungi | NA |
| A0BM88_PARTE | GSPATT00030291001 | Paramecium tetraurelia | Protist | NA |
| A0BNI6_PARTE | GSPATT00030741001 | Paramecium tetraurelia | Protist | NA |
| A0BPF7_PARTE | GSPATT00005173001 | Paramecium tetraurelia | Protist | NA |
| A0CGL4_PARTE | GSPATT00007371001 | Paramecium tetraurelia | Protist | NA |
| A0CK52_PARTE | GSPATT00000882001 | Paramecium tetraurelia | Protist | NA |
| A0CN91_PARTE | GSPATT00008699001 | Paramecium tetraurelia | Protist | NA |
| A0D938_PARTE | GSPATT00014501001 | Paramecium tetraurelia | Protist | NA |
| A0DBL1_PARTE | GSPATT00015324001 | Paramecium tetraurelia | Protist | NA |
| A0DVI7_PARTE | GSPATT00020707001 | Paramecium tetraurelia | Protist | NA |
| A0E0J2_PARTE | GSPATT00021977001 | Paramecium tetraurelia | Protist | NA |
| E0V9U5_PEDHC | Phum_PHUM021780 | Pediculus humanus subsp. Corporis | Animal | Arthropoda |
| E0VKU5_PEDHC | Phum_PHUM269250 | Pediculus humanus subsp. Corporis | Animal | Arthropoda |
| E0VPQ6_PEDHC | Phum_PHUM363230 | Pediculus humanus subsp. Corporis | Animal | Arthropoda |
| E0VX93_PEDHC | Phum_PHUM497560 | Pediculus humanus subsp. Corporis | Animal | Arthropoda |
| C5K6D8_9ALVE | Pmar_PMAR006750 | Perkinsus marinus ATCC 50983 | Protist | NA |
| D0MZ44_PHYIT | PITG_02997 | Phytophthora infestans | Fungi | NA |
| D0ND88_PHYIT | PITG_08806 | Phytophthora infestans | Fungi | NA |
| D0NS69_PHYIT | PITG_15817 | Phytophthora infestans | Fungi | NA |
| C1E8N2_MICSR | MICPUN_108469 | Micromonas sp | Protist | NA |
| B9GLD7_POPTR | POPTRDRAFT_639619 | Populus balsamifera subsp. trichocarpa | Plant | NA |
| B9GY61_POPTR | POPTRDRAFT_647445 | Populus balsamifera subsp. trichocarpa | Plant | NA |
| B2W417_PYRTR | PTRG_05217 | Pyrenophora tritici-repentis | Fungi | NA |
| B2W960_PYRTR | PTRG_06518 | Pyrenophora tritici-repentis | Fungi | NA |
| CAN6_RAT | Capn6 | Rattus norvegicus | Animal | Chordata |
| RIM13_YEAST | RIM13 | Saccharomyces cerevisiae | Fungi | NA |
| F2TVF2_9EUKA | PTSG_00064 | Salpingoeca sp. ATCC 50818 | Protist | NA |
| F2TVF3_9EUKA | PTSG_00065 | Salpingoeca sp. ATCC 50818 | Protist | NA |
| Q5DDD0_SCHJA | Unknown | Schistosoma japonicum | Animal | Platyhelminthe |
| Q5DDE4_SCHJA | Unknown | Schistosoma japonicum | Animal | Platyhelminthe |
| C4Q4U9_SCHMA | Smp_137410 | Schistosoma mansoni | Animal | Platyhelminthe |
| C4Q6Y1_SCHMA | Smp_141640 | Schistosoma mansoni | Animal | Platyhelminthe |
| C4QFW8_SCHMA | Smp_159550 | Schistosoma mansoni | Animal | Platyhelminthe |
| Q0U7L9_PHANO | SNOG_12245 | Phaeosphaeria nodorum | Fungi | NA |
| D1ZIH9_SORMK | SMAC_06666 | Sordaria macrospora | Fungi | NA |
| F1RWU4_PIG | CAPN6 | Sus scrofa | Animal | Chordata |
| Q22UD8_TETTH | TTHERM_00259450 | Tetrahymena thermophila SB210 | Protist | NA |
| Q22V56_TETTH | TTHERM_00575440 | Tetrahymena thermophila SB210 | Protist | NA |
| Q236J8_TETTH | TTHERM_00086990 | Tetrahymena thermophila SB210 | Protist | NA |
| Q23JH1_TETTH | TTHERM_00979860 | Tetrahymena thermophila SB210 | Protist | NA |
| Q4SWM1_TETNG | GSTENG00011418001 | Tetraodon nigroviridis | Animal | Chordata |
| Q4T7L4_TETNG | GSTENG00005675001 | Tetraodon nigroviridis | Animal | Chordata |
| PALB_CANGA | RIM13 | Torulopsis glabrata | Fungi | NA |
| B6KVN2_TOXGO | TGME49_107780 | Toxoplasma gondii | Protist | NA |
| B9Q3H4_TOXGO | TGGT1_043020 | Toxoplasma gondii | Protist | NA |
| D6WGT0_TRICA | TcasGA2_TC002117 | Tribolium castaneum | Animal | Arthropoda |
| D6WGY6_TRICA | TcasGA2_TC030730 | Tribolium castaneum | Animal | Arthropoda |
| D6WUX3_TRICA | TcasGA2_TC005995 | Tribolium castaneum | Animal | Arthropoda |
| A2DDC2_TRIVA | TVAG_013660 | Trichomonas vaginalis | Protist | NA |
| A2DNH6_TRIVA | TVAG_113230 | Trichomonas vaginalis | Protist | NA |
| A2DTE8_TRIVA | TVAG_423090 | Trichomonas vaginalis | Protist | NA |
| A2E1X1_TRIVA | TVAG_164300 | Trichomonas vaginalis | Protist | NA |
| A2FAE4_TRIVA | TVAG_299900 | Trichomonas vaginalis | Protist | NA |
| A2FER3_TRIVA | TVAG_140890 | Trichomonas vaginalis | Protist | NA |
| B3RXT0_TRIAD | TRIADDRAFT_56318 | Trichoplax adhaerens | Animal | Placazoa |
| Q387D8_9TRYP | Tb11.v4.0001 | Trypanosoma brucei | Protist | NA |
| Q388U3_9TRYP | Tb10.389.0470 | Trypanosoma brucei | Protist | NA |
| Q38C53_9TRYP | Tb10.70.5950 | Trypanosoma brucei | Protist | NA |
| Q38EJ3_9TRYP | Tb09.160.5550 | Trypanosoma brucei | Protist | NA |
| Q57WJ7_9TRYP | Tb927.8.8330 | Trypanosoma brucei | Protist | NA |
| Q57XZ8_9TRYP | Tb04.30K5.70 | Trypanosoma brucei | Protist | NA |
| Q57XZ9_9TRYP | Tb04.30K5.110 | Trypanosoma brucei | Protist | NA |
| Q584X4_9TRYP | Tb927.6.3310 | Trypanosoma brucei | Protist | NA |
| Q9GPI8_9TRYP | AF321838.1 | Trypanosoma brucei | Protist | NA |
| Q9GPI9_9TRYP | CAP5.5 | Trypanosoma brucei | Protist | NA |
| D6XEF2_TRYB2 | Tb927.4.3940 | Trypanosoma brucei brucei (strain 927/4 GUTat10.1) | Protist | NA |
| D6XEF3_TRYB2 | Tb927.4.3950 | Trypanosoma brucei brucei (strain 927/4 GUTat10.1) | Protist | NA |
| D6XI64_TRYB2 | Tb927.6.3310 | Trypanosoma brucei brucei (strain 927/4 GUTat10.1) | Protist | NA |
| D6XML0_TRYB2 | Tb927.8.8330 | Trypanosoma brucei brucei (strain 927/4 GUTat10.1) | Protist | NA |
| Q4GZ09_TRYB2 | Tb927.1.2120 | Trypanosoma brucei brucei (strain 927/4 GUTat10.1) | Protist | NA |
| Q4GZ10_TRYB2 | Tb927.1.2110 | Trypanosoma brucei brucei (strain 927/4 GUTat10.1) | Protist | NA |
| Q4GZ11_TRYB2 | Tb927.1.2100 | Trypanosoma brucei brucei (strain 927/4 GUTat10.1) | Protist | NA |
| C9ZIE7_TRYB9 | TbgDal_I1230 | Trypanosoma brucei gambiense (strain MHOM/CI/86/DAL972) | Protist | NA |
| C9ZIE8_TRYB9 | TbgDal_I1240 | Trypanosoma brucei gambiense (strain MHOM/CI/86/DAL972) | Protist | NA |
| C9ZIE9_TRYB9 | TbgDal_I1250 | Trypanosoma brucei gambiense (strain MHOM/CI/86/DAL972) | Protist | NA |
| C9ZN52_TRYB9 | TbgDal_IV4050 | Trypanosoma brucei gambiense (strain MHOM/CI/86/DAL972) | Protist | NA |
| C9ZN53_TRYB9 | TbgDal_IV4060 | Trypanosoma brucei gambiense (strain MHOM/CI/86/DAL972) | Protist | NA |
| C9ZR02_TRYB9 | TbgDal_VI3100 | Trypanosoma brucei gambiense (strain MHOM/CI/86/DAL972) | Protist | NA |
| C9ZWY4_TRYB9 | TbgDal_VIII8670 | Trypanosoma brucei gambiense (strain MHOM/CI/86/DAL972) | Protist | NA |
| C9ZY36_TRYB9 | TbgDal_IX4110 | Trypanosoma brucei gambiense (strain MHOM/CI/86/DAL972) | Protist | NA |
| D0A1K3_TRYB9 | TbgDal_X2280 | Trypanosoma brucei gambiense (strain MHOM/CI/86/DAL972) | Protist | NA |
| D0A5C8_TRYB9 | TbgDal_X15720 | Trypanosoma brucei gambiense (strain MHOM/CI/86/DAL972) | Protist | NA |
| D0A5Q2_TRYB9 | TbgDal_XI1180 | Trypanosoma brucei gambiense (strain MHOM/CI/86/DAL972) | Protist | NA |
| D0A5Q3_TRYB9 | TbgDal_XI1190 | Trypanosoma brucei gambiense (strain MHOM/CI/86/DAL972) | Protist | NA |
| E7L8B7_TRYCR | TCSYLVIO_4314 | Trypanosoma cruzi | Protist | NA |
| E7L8B9_TRYCR | TCSYLVIO_4318 | Trypanosoma cruzi | Protist | NA |
| E7L8C0_TRYCR | TCSYLVIO_4319 | Trypanosoma cruzi | Protist | NA |
| E7L921_TRYCR | TCSYLVIO_4579 | Trypanosoma cruzi | Protist | NA |
| E7LJA1_TRYCR | TCSYLVIO_8244 | Trypanosoma cruzi | Protist | NA |
| E7LLG6_TRYCR | TCSYLVIO_9022 | Trypanosoma cruzi | Protist | NA |
| E7LMU7_TRYCR | TCSYLVIO_9507 | Trypanosoma cruzi | Protist | NA |
| E7LMU8_TRYCR | TCSYLVIO_9508 | Trypanosoma cruzi | Protist | NA |
| E7LMU9_TRYCR | TCSYLVIO_9509 | Trypanosoma cruzi | Protist | NA |
| E7LMV0_TRYCR | TCSYLVIO_9510 | Trypanosoma cruzi | Protist | NA |
| Q4CQB4_TRYCR | Tc00.1047053507829.10 | Trypanosoma cruzi | Protist | NA |
| Q4D4Z7_TRYCR | Tc00.1047053511269.70 | Trypanosoma cruzi | Protist | NA |
| Q4D608_TRYCR | Tc00.1047053508555.70 | Trypanosoma cruzi | Protist | NA |
| Q4D609_TRYCR | Tc00.1047053508555.60 | Trypanosoma cruzi | Protist | NA |
| Q4D610_TRYCR | Tc00.1047053508555.50 | Trypanosoma cruzi | Protist | NA |
| Q4D6J0_TRYCR | Tc00.1047053506721.30 | Trypanosoma cruzi | Protist | NA |
| Q4DB11_TRYCR | Tc00.1047053511507.70 | Trypanosoma cruzi | Protist | NA |
| Q4DCK2_TRYCR | Tc00.1047053503909.84 | Trypanosoma cruzi | Protist | NA |
| Q4DE63_TRYCR | Tc00.1047053506493.90 | Trypanosoma cruzi | Protist | NA |
| Q4DJS6_TRYCR | Tc00.1047053509237.140 | Trypanosoma cruzi | Protist | NA |
| Q4DJS7_TRYCR | Tc00.1047053509237.130 | Trypanosoma cruzi | Protist | NA |
| Q4DJS8_TRYCR | Tc00.1047053509237.120 | Trypanosoma cruzi | Protist | NA |
| Q4DQ12_TRYCR | Tc00.1047053510121.170 | Trypanosoma cruzi | Protist | NA |
| Q4DQN5_TRYCR | Tc00.1047053506563.210 | Trypanosoma cruzi | Protist | NA |
| Q4DQN6_TRYCR | Tc00.1047053506563.200 | Trypanosoma cruzi | Protist | NA |
| Q4DQN7_TRYCR | Tc00.1047053506563.190 | Trypanosoma cruzi | Protist | NA |
| Q4E0D6_TRYCR | Tc00.1047053508999.220 | Trypanosoma cruzi | Protist | NA |
| Q4E0D7_TRYCR | Tc00.1047053508999.200 | Trypanosoma cruzi | Protist | NA |
| Q4E0D8_TRYCR | Tc00.1047053508999.190 | Trypanosoma cruzi | Protist | NA |
| F2EA39_HORVD | Unknown | Hordeum vulgare var. distichum | Plant | NA |
| D8TN33_VOLCA | mot15 | Volvox carteri f. nagariensis | Protist | NA |
| D8TN36_VOLCA | VOLCADRAFT_103605 | Volvox carteri f. nagariensis | Protist | NA |
| D8TYQ1_VOLCA | VOLCADRAFT_105151 | Volvox carteri f. nagariensis | Protist | NA |
| D8UEL0_VOLCA | VOLCADRAFT_98154 | Volvox carteri f. nagariensis | Protist | NA |
| D8UHG2_VOLCA | adk7 | Volvox carteri f. nagariensis | Protist | NA |
| B4FFL6_MAIZE | Unknown | Zea mays (Maize) | Plant | NA |

^a^ NA = Not applicable
