# Supplementary material for: The Atypical Calpains: Evolutionary Analyses and Roles in Caenorhabditis elegans Cellular Degeneration
Source: PLoS Genet. 2012 Mar 29;8(3):e1002602. doi: 10.1371/journal.pgen.1002602 (PMC3315469; doi:10.1371/journal.pgen.1002602)
Supplement: Table S2 — Effects of calpain mutants and feeding RNAi on brood size and embryonic lethality. (DOC) [file pgen.1002602.s017.doc]

**Table S2. Effects of calpain mutants and feeding RNAi on brood size and embryonic lethality.**

| Gene | n^a^ | Brood size | Embryonic lethality (%) | Relative gene expression^c^ | Indel position^f^ | Catalytic triad disruption^g^ |
| --- | --- | --- | --- | --- | --- | --- |
| *clp-1(tm690)* | 4 | 271 ± 14 | 1.3 ± 0.9% | 0.3 ± 0.04 | 2800-3423 bp | Y |
| *clp-2(pk323)* | 4 | 232 ± 13 | 0.3 ± 0.3% | 0^d^ | 2481 bp | N |
| *clp-3(RNAi)* | 8 | 302 ± 10 | 0 ± 0% | 0^d^ | N/A | N/A |
| *clp-4(ok2808)* | 4 | 261± 17 | 3.5 ± 1.2% | 0.3 ± 0.04 | 3268-3853 bp | N |
| *clp-6(ok1779)* | 4 | 291 ± 17 | 2.8 ± 0.5% | 0.3 ± 0.03 | 1619-4005 bp | Y |
| *clp-7(ok2750)* | 4 | 262 ± 9 | 1.5 ± 0.6% | 0.3 ± 0.04 | 8442-8654 bp | N |
| *clp-8(ok1878)* | 4 | 247± 21 | 1 ± 0.6% | 0.2 ± 0.01 | 405-1999 bp | Y |
| *clp-9(ok1866)* | 4 | 252 ± 10 | 1.2 ± 0.4% | 0^d^ | 1056-2212 bp | Y |
| *clp-10(ok2713)* | 4 | 144 ± 8 | 2.3 ± 0.6% | 0^d^ | 1570-3335 bp | Y |
| *clpr-1(ok2601)* | 4 | 217 ± 7 | 4.8 ± 0.3% | 0^d+e^ | 164-1841 bp | N/A |
| N2^b^ | 4 | 287 ± 8 | 0 ± 0% | N/A | N/A | N/A |

^a^ n represents the number of broods counted.

^b^ N2 wildtype control animals were fed on empty L4440 vector in HT115 host bacteria.

^c^ Gene expression measured by qPCR was normalized to *ama-1* expression taken as a value of 1 (relative expression calculated using the ΔΔCT method).

^d^ No amplification of *clp* mRNA from *clp* mutant or *clp* (*RNAi*) animals after 40 cycles.

^e^ Only one exon is upstream of deletion site, thus amplified qPCR product overlaps with deletion position.

^f^ All of the mutants are deletions, except *clp-2(pk323)*, which contains a 1612 bp Tc1 insertion in an exon and *clp-3*. The coordinates of indels are based on the genomic sequence starting from the ATG.

^g^ Indels predicted to remove one or more catalytic residues are indicated with (Y) and those that retaining an intact triad with (N). N/A, not applicable. *clpr-1* cDNA naturally lacks two catalytic residues.

Error is ± standard error of the mean (SEM).
