# Supplementary material for: The Atypical Calpains: Evolutionary Analyses and Roles in Caenorhabditis elegans Cellular Degeneration
Source: PLoS Genet. 2012 Mar 29;8(3):e1002602. doi: 10.1371/journal.pgen.1002602 (PMC3315469; doi:10.1371/journal.pgen.1002602)
Supplement: Table S3 — Effects of ectopic clp-1 expression on brood size and embryonic lethality. (DOC) [file pgen.1002602.s018.doc]

**Table S3**. **Effects of ectopic *clp-1* expression on brood size and embryonic lethality.**

| **Genotype** | **Brood size^a^** | **Embryonic lethality (%)** |
| --- | --- | --- |
| *N2* | 229 ± 6.0 | 0 ± 0% |
| *crIs4* | 224 ± 5.0 | 0.9 ± 0.3% |
| *dys-1(cx18);egl-19(ad695gf);crIs4* | 73 ± 18.7 | 8.2 ± 4.8% |

^a^ Three broods were analysed per strain. Error is ± SEM
