# Supplementary material for: The Atypical Calpains: Evolutionary Analyses and Roles in Caenorhabditis elegans Cellular Degeneration
Source: PLoS Genet. 2012 Mar 29;8(3):e1002602. doi: 10.1371/journal.pgen.1002602 (PMC3315469; doi:10.1371/journal.pgen.1002602)
Supplement: Table S4 — qPCR analysis of asp-1 to asp-6 (RNAi). (DOC) [file pgen.1002602.s019.doc]

**Table S4: qPCR analysis of *asp-1* to *asp-6* (*RNAi*).**

| **Gene** | **Relative gene expression^a^** |
| --- | --- |
| *asp-1(RNAi)* | 0.5 ± 0.02 |
| *asp-2(RNAi)* | 0.3 ± 0.04 |
| *asp-3(RNAi)* | 0.08 ± 3E-3 |
| *asp-4(RNAi)* | 0.03 ± 6E-4 |
| *asp-5(RNAi)* | 0.3 ± 0.02 |
| *asp-6(RNAi)* | 0.5 ± 0.06 |

^a^ Gene expression measured by qPCR in each of the mutants is normalised to *ama-1* expression taken as a value of 1 (relative expression calculated using the ΔΔCT method). Error is ± SEM.
